# Synthesis and Biological Evaluation of Modified Peptide Derivatives Targeting the SARS-CoV‑2 Nsp3 Macrodomain (Mac1) Replication Domain

**DOI:** 10.1021/acsomega.6c02033

**Published:** 2026-04-24

**Authors:** Özge Özmen, Betül Oruçoğlu, Serap İpek Dingiş Birgül, Dilek Öztürk Civelek, Şeref Gül, Atilla Akdemir, Fatih Sönmez, Belma Zengin Kurt

**Affiliations:** † Bezmialem Vakif University, Faculty of Pharmacy, Istanbul 34093, Türkiye; ‡ Kartal Dr. Lutfi Kirdar City Hospital, Istanbul 34865, Türkiye; § Institute of Life Sciences and Biotechnology, Bezmialem Vakif University, Istanbul 34820, Türkiye; ∥ Department of Pharmaceutical Chemistry, Faculty of Pharmacy, 221265 Bezmialem Vakif University, Istanbul 34093, Türkiye; ⊥ Department of Pharmacology, Faculty of Pharmacy, Istanbul University -Cerrahpaşa, Istanbul 34500, Türkiye; # Department of Pharmacology, Faculty of Pharmacy, Bezmialem Vakif University, Istanbul 34093, Türkiye; ∇ Department of Pharmacology, Faculty of Pharmacy, 510311 Istanbul Kent University, Istanbul 34406, Türkiye; ○ Pamukova Vocational School, Sakarya University of Applied Sciences, Sakarya 54055, Türkiye

## Abstract

Modified peptide derivatives (**D1–D15**) containing hydrophobic (phenylalanine and tryptophan) and positively charged (histidine) amino acid residues, were designed and synthesized. The cytotoxicity of the compounds was evaluated in healthy (CCD1079Sk) cell lines, revealing no significant cytotoxic effects and indicating favorable safety profiles. Molecular modeling studies were performed for all compounds and indicated that compounds **D13**, **D14**, and **D15** form favorable binding interactions with the Nsp3 macrodomain 1 (Mac1) active site. The affinity of these compounds is expected to be moderate to high, although lower than that of ADP-ribose. Subsequent bioactivity assays, performed using a cell-based SARS-CoV-2 replicon system, revealed that compound **D15** displays the most potent inhibition of viral replication (IC_50_ = 22.2 μM (95% CI: 15.4–35.7 μM)), while compound **D14** shows the second most potent inhibitory activity (IC_50_ = 61.2 μM (95% CI: 39.2–143.9 μM)). Considering these results, it has been shown that compounds with a histidine side chain exhibit higher biological activity, suggesting a structure–activity relationship driven by positively charged residues.

## Graphical abstract

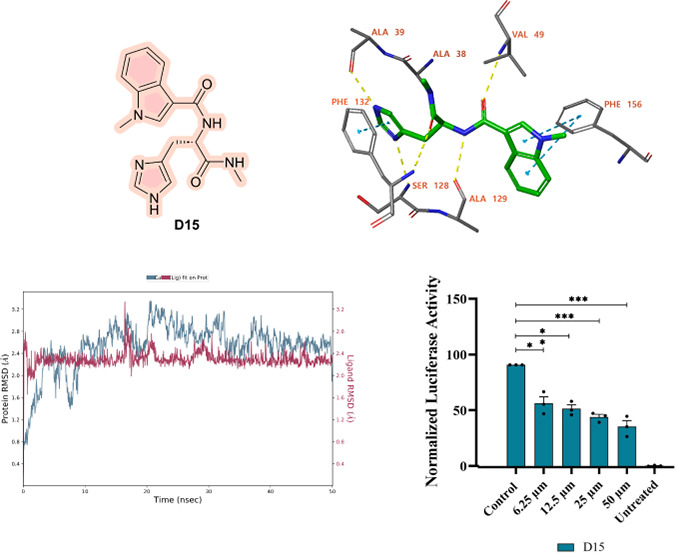

## 1 Introduction

Severe Acute Respiratory Syndrome Coronavirus 2 (SARS-CoV-2), the causative agent of the COVID-19 pandemic, is an enveloped, positive-sense, single-stranded RNA virus classified within the *Betacoronavirus* genus within the *Coronaviridae* family.^1,2^ Morphologically, the virus exhibits a roughly spherical to pleomorphic shape, approximately 60–140 nm in diameter. Its outer lipid envelope, derived from the host cell membrane, contains three main structural proteins: the spike (S), membrane (M), and envelope (E) proteins. These play essential roles in host cell recognition, membrane fusion, and viral assembly. Internally, the viral RNA genome is associated with the nucleocapsid (N) protein, forming a ribonucleoprotein complex critical for genome packaging and replication.^3,4^ The ∼30 kb genome of SARS-CoV-2 is among the largest known RNA viral genomes. It contains a 5′-cap and 3′-poly­(A) tail and is organized into several open reading frames (ORFs). Nsp1–11 are generated from pp1a encoded by ORF1a, while a −1 ribosomal frameshift extends translation into ORF1b, to produce Nsp1–16 within pp1ab.^5^

Nsp3 is the largest multidomain protein encoded by coronaviruses and exhibits variations in domain organization across different CoV genera.^6^ Bioinformatic analyses indicate that it comprises approximately 10–16 domains, of which eight domains and two transmembrane regions are conserved. Nsp3 is released from the pp1a/pp1ab polyproteins by its intrinsic papain-like protease domain(s). Functionally, it participates in multiple stages of the viral life cycle, acting as a scaffold for interactions with viral Nsps and host proteins, and playing a critical role in the formation of the replication/transcription complex (RTC).^5^ In SARS-CoV-2, Nsp3 contains three macrodomain folds, including Mac1 and two SUD-M-like domains (SUD-M-N and SUD-M-C). The Mac1 domain exhibits mono (ADP-ribosyl) hydrolase activity in vitro, enabling the removal of ADP-ribose modifications, such as those introduced by PARP14, from host proteins. Due to its role in modulating host immune responses and its association with viral virulence in related coronaviruses, the Mac1 domain of Nsp3 has been identified as a promising therapeutic target.^7–9^

A wide range of structures, predominantly peptidomimetic in nature, have been reported to exhibit inhibitory activity against SARS-CoV-2. These include statine-based scaffolds, tetrapeptidomimetic analogues, and clinically validated molecules such as nirmatrelvir—the active component of Paxlovid (Figure 1).^10–13^ In addition, numerous compounds targeting the Nsp3 (Mac1) domain have been reported in the literature. For example, Pfannenstiel et al. reported promising inhibitory activity for azoindole derivatives,^14,15^ while Joshi et al. performed in silico studies on pyrazoline-based compounds and predicted their potential efficacy.^16^ Furthermore, Gahbauer et al. employed crystallographic screening to identify potent Mac1 inhibitors^17^ and Schuller et al. conducted similar investigations to explore potential inhibitory compounds.^18^

**Figure 1:**
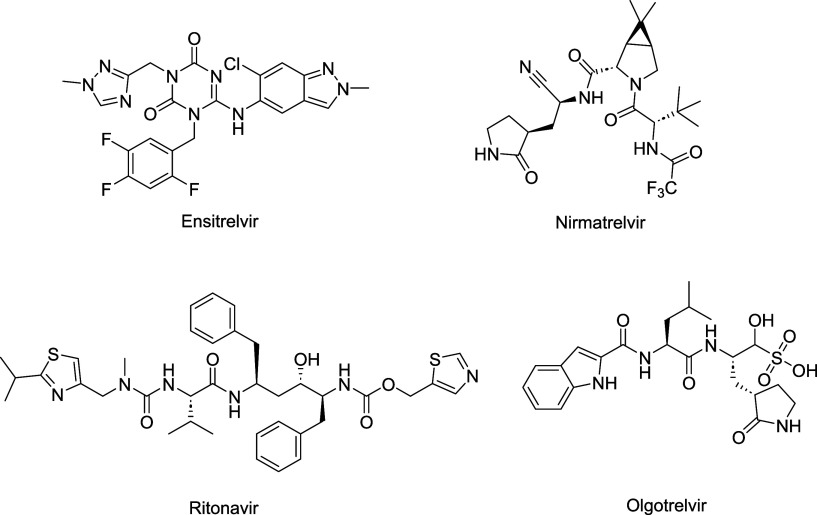
Chemical structures of some known inhibitors of SARS-CoV-2.

These studies indicate that a substantial portion of current research remains focused on the theoretical identification of potential compounds and chemical scaffolds, primarily through in silico approaches and screening methods. While these efforts provide valuable preliminary insights, the number of experimentally validated and highly potent inhibitors is still limited. Therefore, there remains a clear need for the design, synthesis, and biological evaluation of new compounds in this field.

In this study, we designed and synthesized modified peptide derivatives and subsequently investigated their cytotoxicity. Molecular modeling studies indicated reasonable to strong binding interactions of compounds **D13**, **D14** and **D15** with the Nsp3 (Mac1) active site. Cell -based bioactivity assays, performed using a SARS-CoV-2 replicon system that enables the assessment of viral RNA replication independently of infectious virus production, demonstrated that these peptides inhibit replicon replication at micromolar concentrations, thereby demonstrating their antiviral activity at the cellular level.

## 2 Results and Discussion

### 2.1 Chemistry

This study successfully synthesized a series of peptide compounds bearing hydrophobic (phenylalanine and tryptophan) and positively charged (histidine) amino acid side chains in two steps (Scheme 1). The first step involved converting amino acid esters into *N*-methyl amides (**A–C**), followed by coupling reactions between the amino groups of the amino acids and various carboxylic acid derivatives in the second step. Conversion to *N*-methyl amides (**A–C**) was achieved by treating the ester compounds with a 33% MeNH_2_ solution for 48 h, yielding 78%, 72%, and 68%, respectively, following the method described in the literature.^19^ Afterward, a coupling reaction was carried out between the obtained **A–C** derivatives and acid derivatives in the presence of DCC, DIPEA, and HOBt in DMF at room temperature to afford the **D1–D15** derivatives.^20^

**Scheme 1:**
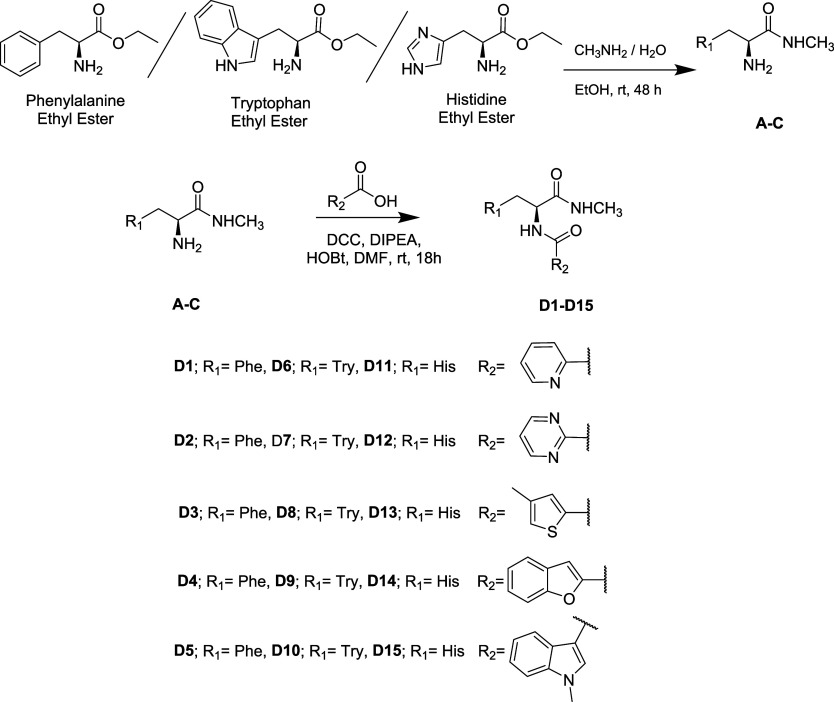
Synthesis of Peptidomimetic Derivatives

From the ^1^H NMR spectrum of compound **A–C**, the ester-to-amide conversion causes the multiple peaks belonging to the ester to disappear, while the amide-to-NH-CH_3_ signal is observed as a singlet or doublet around 2.72–2.77 ppm. The ^13^C NMR spectrum exhibited a characteristic resonance for the carbonyl carbon at around 175 ppm. Additional resonances corresponding to aromatic carbons were observed in the 110–138 ppm region, whereas aliphatic carbons appeared in the range of 26–56 ppm.

For compounds **D1–D15**, ^1^H NMR spectra revealed aromatic proton resonances within the range of 5.90–8.90 ppm and aliphatic proton resonances within the range of 2.10–4.75 ppm. NH protons produced signals in the region of 9.6–10.7 ppm. ^13^C NMR spectra showed aliphatic carbon signals between 15.6 and 56.5 ppm and aromatic carbon signals between 108 and 158 ppm. Signals corresponding to the amide carbon were recorded in the range of 161–172 ppm.

### 2.2 Molecular Modeling Studies

#### 2.2.1 Validation

A retrospective docking was performed in which the cocrystallized ligand ADP-ribose was docked into the binding pocket of SARS-CoV-2 Nsp3 (Mac1) structure (PDB ID: 7KQP)^21^ using the SP mode. The docked and cocrystallized pose of the ligand was very similar (RMSD: 1.42 Å; Figure 2), indicating the reliability of the pose generation.

**Figure 2:**
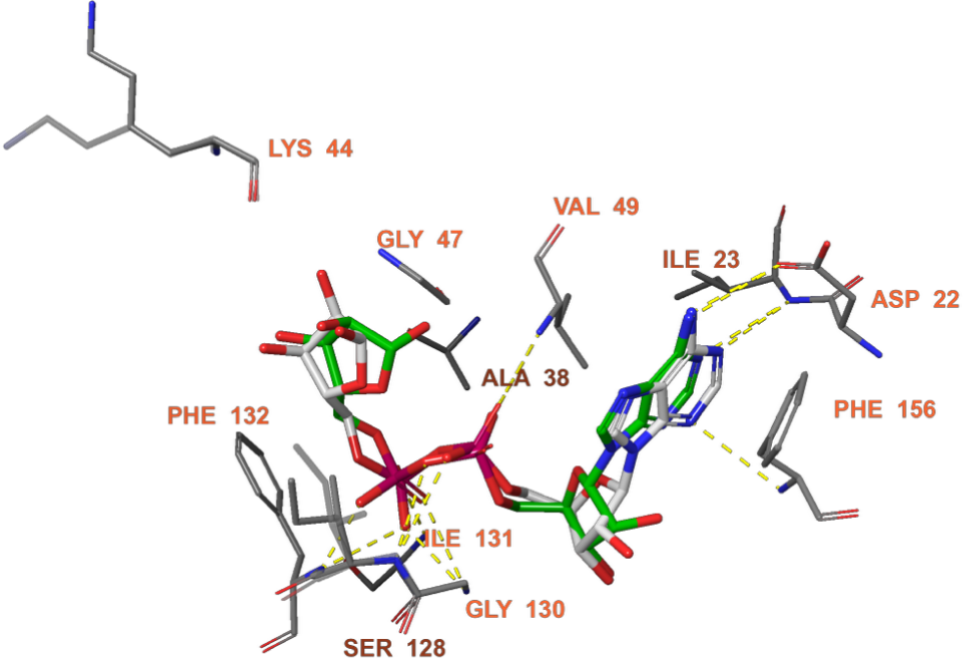
Docked pose (green) and cocrystallized pose (gray) of ADP-ribose.

Subsequently, the cocrystal structure was subjected to a 250 ns MD simulation (Figure 3). At the start of the MD simulation, the cocrystallized ligand forms hydrogen bonds with several key residues. The ribose moiety interacts with the backbone atoms of Gly47, while the phosphate groups of the ligand form hydrogen bonds with the backbone of Ser128 and Ile131. The adenosine portion of the ligand establishes hydrogen bonds with the Asp22 side chain and the backbone atoms of Ile23 and Phe156, collectively stabilizing the ligand within the binding pocket.

**Figure 3:**
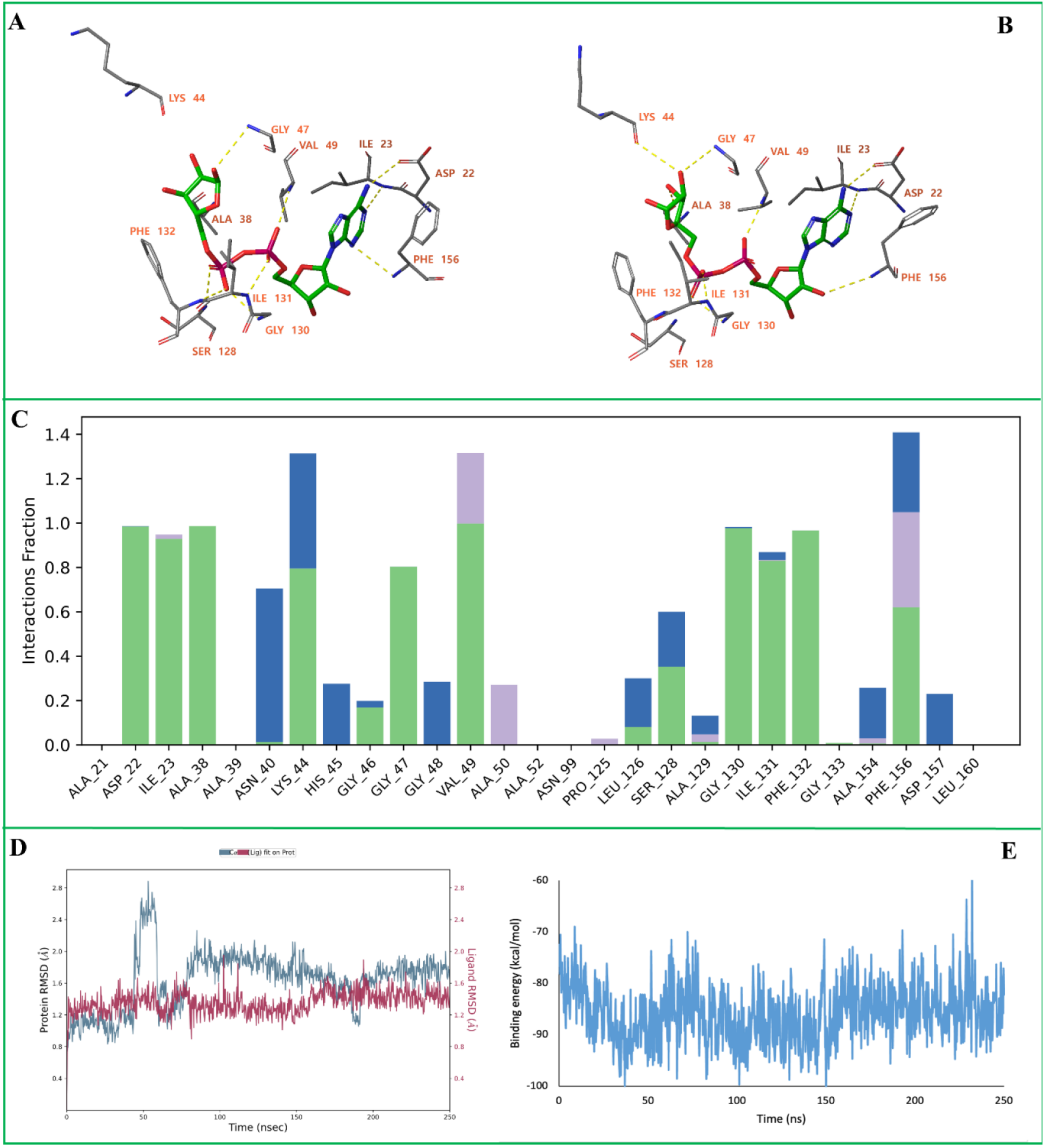
MD simulation of the SARS-CoV-2 Nsp3 (Mac1) in complex with the cocrystallized ligand. (A) Initial binding poses of the ligand. (B) Final binding poses of the ligand after 250 ns MD simulation. (C) Protein–ligand interaction histogram showing the frequency and type of interactions formed during the trajectory. (D) Root Mean Square Deviation (RMSD) plots of the protein backbone (Cα atoms, blue) and the ligand (red). (E) MM-GBSA binding free energy profile calculated over the 250 ns trajectory. In panels (A–B), yellow dashed lines represent hydrogen bonds, while blue dashed lines indicate π–π stacking interactions. In panel (C), green bars correspond to hydrogen bonds, blue bars represent water-mediated hydrogen bonds, and purple bars denote hydrophobic contacts.

During the simulation, the ligand maintains hydrogen bonds with the Asp22 side chain, the backbone atoms of Ile23, Gly47, Val49, and Ile131, and the backbone of Phe156. Additionally, hydrogen bonds were formed with the backbone atoms of Ala38 and Lys44, both interacting with the terminal ribose moiety of the ligand, further stabilizing its binding within the pocket. Analyses of the interaction histogram indicated that hydrogen bonds with Asp22, Ile23, Ala38, Val49, Gly130, Ile131, and Phe132 were persistently maintained throughout the simulation, suggesting stable and long-lasting interactions. The ligand RMSD remained consistently below 2 Å, demonstrating tight binding within the active site, whereas the protein RMSD stabilized around 1.5–2.0 Å indicating that the overall conformation of Nsp3 (Mac1) remained largely stable. In addition, MM-GBSA binding free energy calculations yielded an average ΔG bind of approximately −85 ± 6 kcal/mol, further supporting the high binding affinity of the ligand. Collectively, these results confirm that the cocrystallized ligand maintains a stable binding mode within the active site of SARS-CoV-2 Nsp3 (Mac1), in agreement with the experimental structure.

#### 2.2.2 Molecular Docking Studies

The binding interactions of compounds **D1–D15** with the SARS-CoV-2 Nsp3 (Mac1) active site were initially investigated with docking studies (Figure 4). The docked poses of compounds **D13**, **D14** and **D15** were selected for further molecular dynamics simulations due to their binding interactions and the score (Figures 5–7).

**Figure 4:**
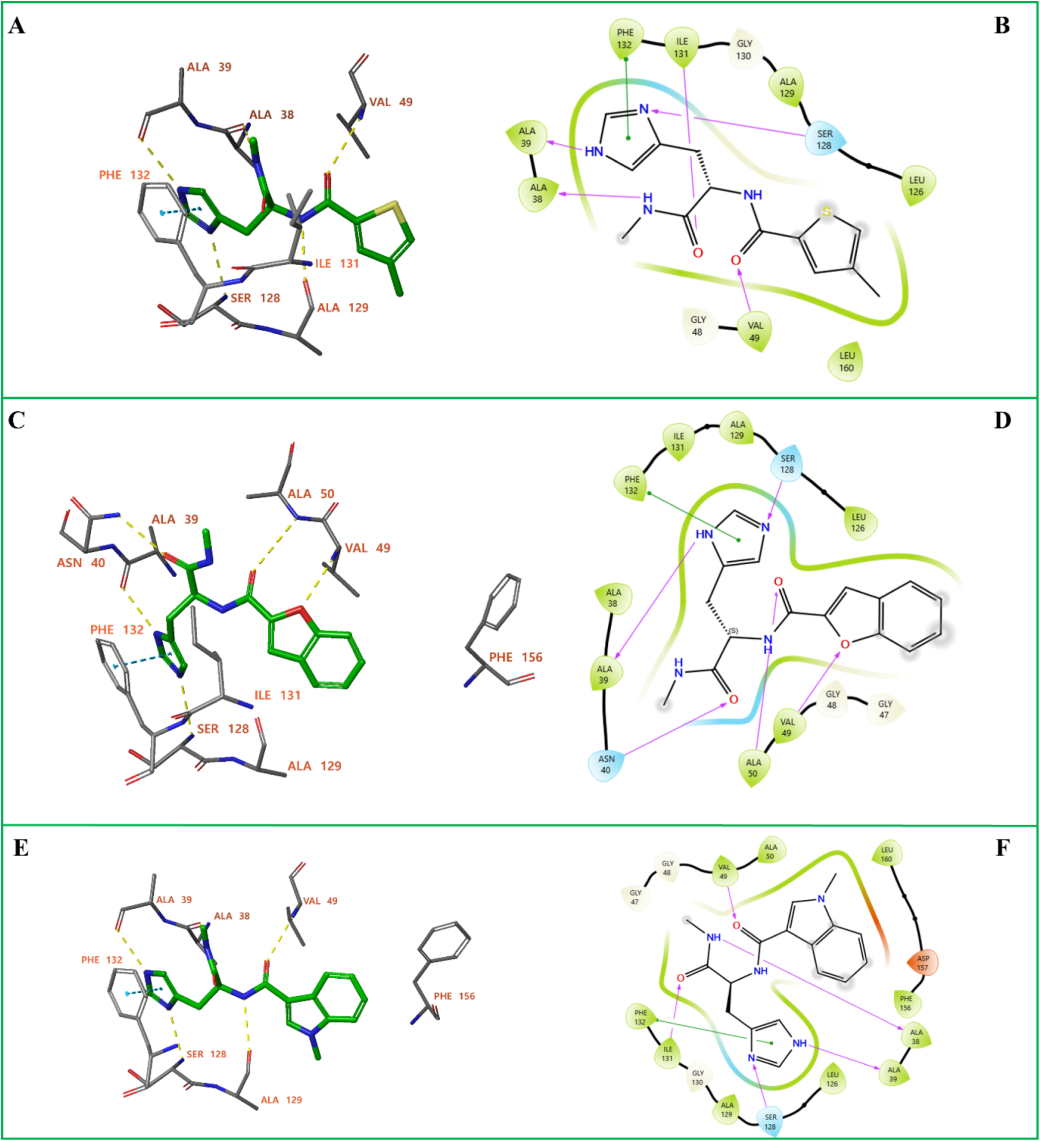
Docked poses and two-dimensional interaction analyses of compounds **D13**, **D14** and **D15** in the Nsp3 (Mac1) binding site. (A) Docking pose of **D13** in the binding site of the Nsp3 (Mac1). (B) 2D interaction diagram of **D13** in complex with Nsp3 (Mac1). (C) Docking pose of **D14** in the binding site of the Nsp3 (Mac1). (D) 2D interaction diagram of **D14** in complex with Nsp3 (Mac1). (E) Docking pose of **D15** in the binding site of the Nsp3 (Mac1). (F) 2D interaction diagram of **D15** in complex with Nsp3 (Mac1). In panels (A, C, E), yellow dashed lines represent hydrogen bonds, while blue dashed lines indicate π–π stacking interactions. In panels (B, D, F) π–π stacking interactions are shown in green, while hydrogen bonds are represented by purple arrows directed from the hydrogen bond donor toward the acceptor. Hydrophobic amino acids are shown in green, negatively charged amino acids in red, and polar amino acids in blue. Solvent exposure of ligand atoms is indicated by surrounding gray halos.

**Figure 5:**
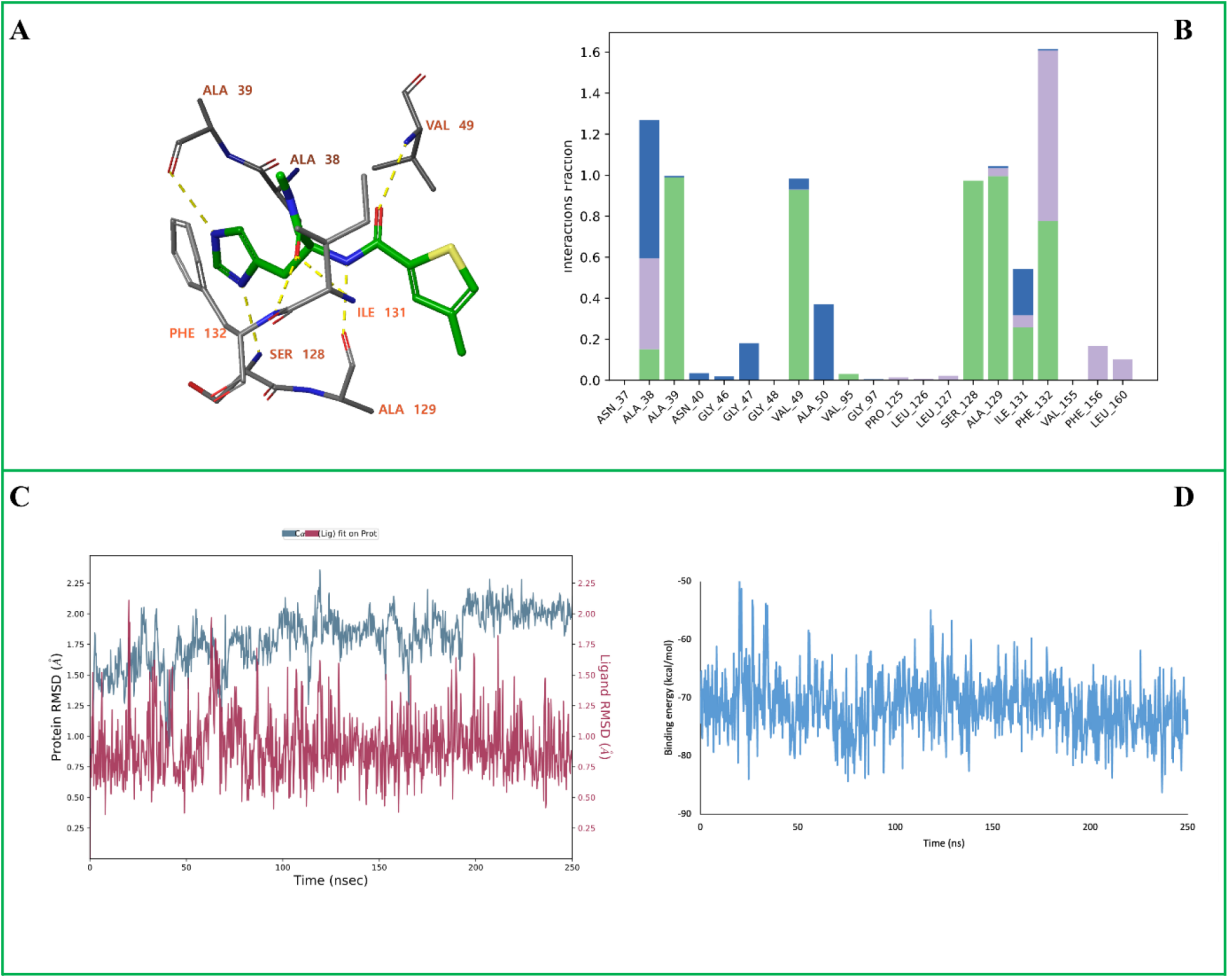
MD simulation analysis of the SARS-CoV-2 Nsp3 (Mac1) in complex with **D13**. (A) The ligand–protein binding interactions after 250 ns MD simulation. (B) Protein–ligand interaction histogram showing the frequency and type of interactions formed during the trajectory. (C) Root Mean Square Deviation (RMSD) plots of the protein backbone (Cα atoms, blue) and the ligand (red). (D) MM-GBSA binding free energy profile calculated over the 250 ns trajectory. In panel (A), yellow dashed lines represent hydrogen bonds, while blue dashed lines indicate π–π stacking interactions. In panel (B), green bars correspond to hydrogen bonds, blue bars represent water-mediated hydrogen bonds, and purple bars denote hydrophobic contacts.

**Figure 6:**
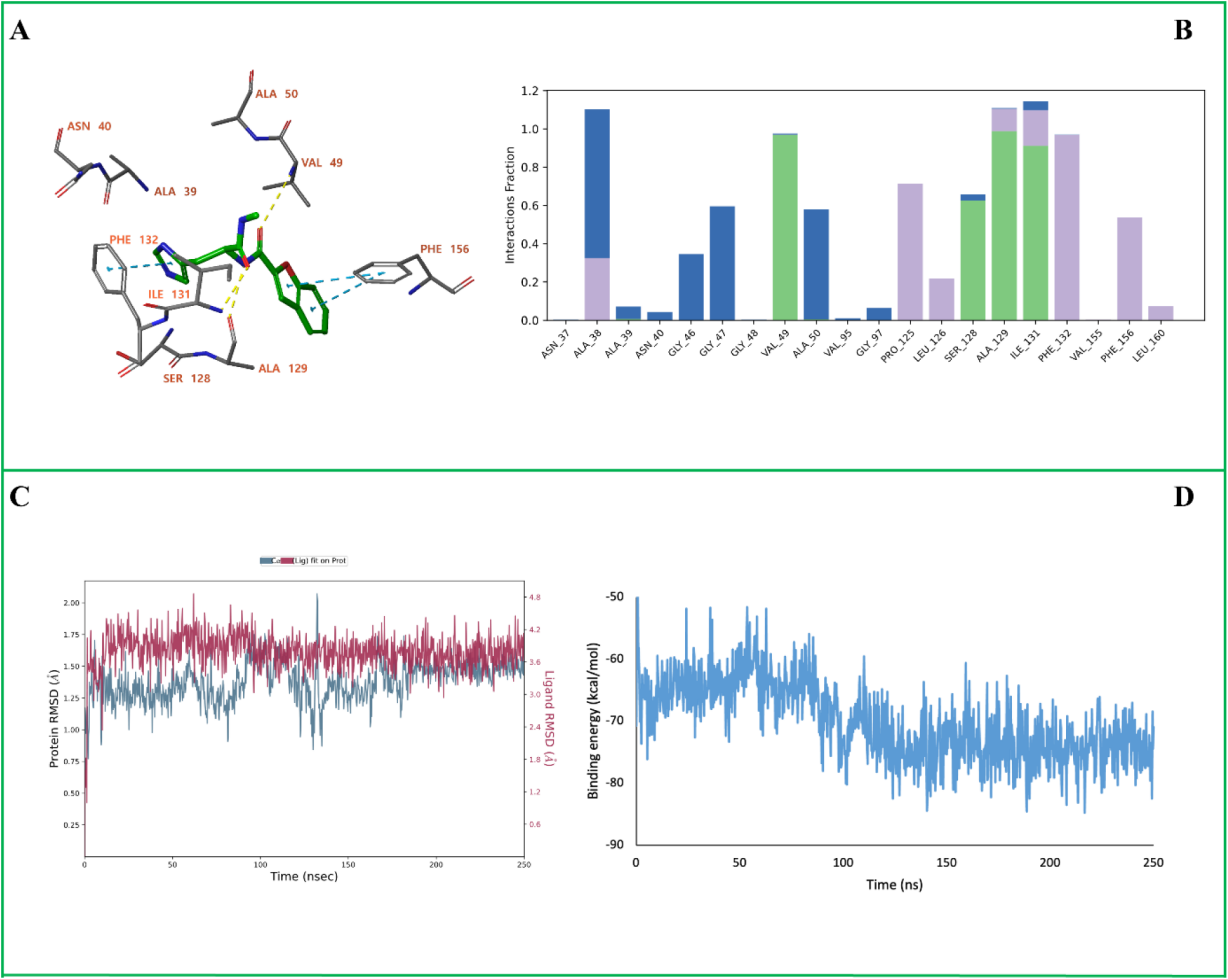
MD simulation analysis of the SARS-CoV-2 Nsp3 (Mac1) in complex with **D14**. (A) The ligand–protein binding interactions after 250 ns MD simulation. (B) Protein–ligand interaction histogram showing the frequency and type of interactions formed during the trajectory. (C) Root Mean Square Deviation (RMSD) plots of the protein backbone (Cα atoms, blue) and the ligand (red). (D) MM-GBSA binding free energy profile calculated over the 250 ns trajectory. In panels (A), yellow dashed lines represent hydrogen bonds, while blue dashed lines indicate π–π stacking interactions. In panel (B), green bars correspond to hydrogen bonds, blue bars represent water-mediated hydrogen bonds, and purple bars denote hydrophobic contacts.

**Figure 7:**
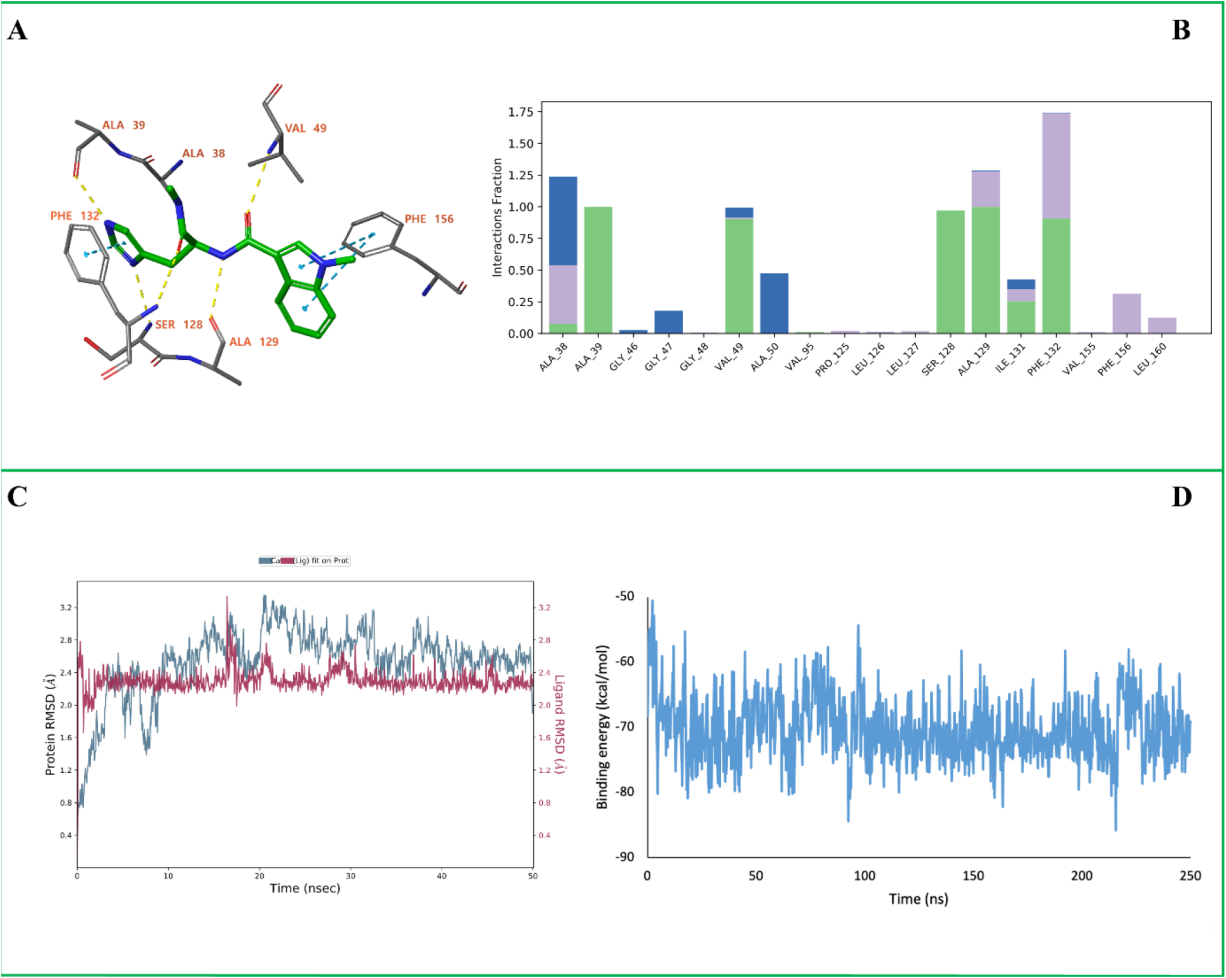
MD simulation analysis of the SARS-CoV-2 Nsp3 (Mac1) in complex with **D15**. (A) The ligand–protein binding interactions after 250 ns MD simulation. (B) Protein–ligand interaction histogram showing the frequency and type of interactions formed during the simulation. (C) Root Mean Square Deviation (RMSD) plots of the protein backbone (Cα atoms, blue) and the ligand (red). (D) MM-GBSA binding free energy profile calculated over the 250 ns trajectory. In panel (A), yellow dashed lines represent hydrogen bonds, while blue dashed lines indicate π–π stacking interactions. In panel (B), green bars correspond to hydrogen bonds, blue bars represent water-mediated hydrogen bonds, and purple bars denote hydrophobic contacts.

Compound **D13** binds to the Nsp3 (Mac1) active site with a docking score of −9.452 kcal/mol. The ligand forms hydrogen bonds with Ala38, Ala39, Val49, Ser128, and Ala129, and engages in π–π stacking with Phe132 via its histidine moiety (Figure 4A,B).

The docked pose of compound **D14** forms hydrogen bonds with the backbone atoms of Ala39, Val49, Ala50, and Ser128, and the side chain of Asn40, with a docking score of −10.693 kcal/mol (Figure 4C,D). In addition, it also participates in π–π stacking with Phe132 via its histidine moiety.

In the docked pose of compound **D15**, hydrogen bonding interactions are formed with the backbone of Ala39, Val49, Ser128, and Ala129, with a docking score of −10.064 kcal/mol (Figure 4E,F). Again, π–π stacking between the histidine moiety of the ligand and the with Phe132 side chain is observed.

#### 2.2.3 MD Simulations

To investigate the dynamic behavior and stability of the selected Nsp3 (Mac1)-ligand complexes, molecular dynamics (MD) simulations were performed.

The MD simulation of the Nsp3 (Mac1)-**D13** complex shows only minor changes in the binding pose. The ligand retained hydrogen bonds with Ala39, Val49, Ser128, and Ala129, while the hydrogen bond with Ala38 was lost and a new hydrogen bond formed with Ile131 (Figure 5A). Hydrogen bonds with Ala39, Val49, Ser128, Ala129, and Phe132 were maintained for the majority of the simulation, indicating persistent and stable interactions (Figure 5B). The ligand RMSD remained between 0.5 and 2.20 Å throughout the trajectory, indicating a tight fit within the active site, while the protein backbone RMSD fluctuated between 1.25 and 2.30 Å, reflecting overall structural stability (Figure 5C). MM-GBSA binding free energy analysis yielded an average ΔG bind of approximately −72 ± 5 kcal/mol, supporting a strong binding affinity of the ligand (Figure 5D).

During the simulation the **D14** ligand binding pose showed minor changes. The hydrogen bonding interactions with Ala39, Ala50, Asn40, and Ser128 were broken, while the interaction with the backbone of Val49 was maintained (Figure 6A). New hydrogen bonds were established with the backbone atoms of Ala129 and Ile131. The π–π stacking with Phe132 was preserved, and an additional π–π stacking interaction formed with Phe156. During the MD simulation, Val49, Ser128, Ala129, Ile131, and Phe132 contributed to stable binding interactions that persisted during most of the simulation (Figure 6B). The ligand RMSD fluctuates around 3.6–4.2 Å and is consistent with the minor change in binding pose of the ligand. The protein backbone conformation did not change significantly during the simulation as indicated the low RMSD value in the range of 1.25–1.50 Å for the Cα-atoms (Figure 6C). The calculated MM-GBSA free energy of binding has an average value of −70 ± 6 kcal/mol (Figure 6D).

Again, the initial binding interactions of **D15** were slightly changed during the MD simulation (Figure 7A). The ligand retained hydrogen bonds with Ala39, Val49, Ala129, and Ser128, and an additional hydrogen bond formed with the backbone of Ser128. The π–π stacking with Phe132 was preserved and a new π–π stacking interaction formed with Phe156. The interaction diagram highlighted the persistent contribution of Ala39, Val49, Ser128, Ala129 and Phe132 to ligand stabilization (Figure 7B). The ligand RMSD stabilized around 2.4–2.8 Å after an initial equilibration phase, which is consistent with the observed changes in the ligand’s binding mode (Figure 7C). The protein backbone does not show significant changes in conformation (RMSD Cα-atoms: 2.0–2.4 Å). The calculated MM-GBSA free energy of binding has an average value of −70 ± 8 kcal/mol (Figure 7D).

#### 2.2.4 Evaluation of Synthesized Compounds for Their Inhibitory Effects on SARS-CoV-2 Replicon Replication

First, the cytotoxicity of the synthesized compounds was evaluated on a healthy cell line (CCD-1079Sk). The IC_50_ values of **D1–D4**, **D6–D10** and **D12–D15** were obtained as >200 μM, while those of **D5** and **D11** were calculated as 89.99 ± 34.83 μM and 154.62 ± 32.49 μM, respectively. The findings revealed that the newly synthesized compounds exhibited no cytotoxic effects on healthy cell lines (Supplementary Figure S1). In brief, cells were cultured overnight, treated with the peptides at varying concentrations for 24 h, and subsequently subjected to MTT cytotoxicity assay.

Subsequently, cell culture-based in vitro analyses were performed using a subgenomic replicon of SARS-CoV-2 previously employed for the identification of SARS-CoV-2 inhibitors.^15,29^ In brief, SARS-CoV-2 subgenomic replicon particles were generated by cotransfecting Huh7 cells with the ΔS-Luc-GFP bacmid and a VSV-G expression plasmid. For antiviral assays, Caco-2 cells were seeded 24 h prior to infection, infected with replicon particles for 2–3 h, washed, and then treated with test compounds. After 16–18 h of incubation, luciferase activity was measured to assess antiviral effects. This experimental approach enabled the evaluation of potential inhibitory activities of the selected compounds (**D6**, **D7**, **D12**, **D14**, and **D15**) against viral replication.^22–24^ These compounds were selected based on molecular modeling results, considering both their high predicted affinity for the target and their favorable structural features. The human intestinal epithelial cell line Caco-2, known for its high susceptibility to SARS-CoV-2 infection, was employed as the model system in the experiments.^22,25^ Initially, MTT viability assays were performed at 50 μM and 100 μM to assess their cytotoxic effects on Caco-2 cells, confirming that these compounds did not adversely affect cell viability (Supplementary Figure S2), thus ensuring that the observed antiviral effects were not attributable to cytotoxicity.

The compounds confirmed noncytotoxic effects were further evaluated using a single-cycle infectious SARS-CoV-2 viral replicon particle (ΔS-VRP) system, which carries luciferase reporter and allows experiments to be conducted under BSL-2 conditions.^23^ As described in the Methods section, the SARS-CoV-2 subgenomic replicon was transfected into Caco-2 cells. 4 h post-transfection, wells were treated with compounds at a final concentration of 50 μM. Control wells received an equivalent amount of DMSO as in the compound-treated wells. Cells were harvested 18 h following compound treatment, and luciferase activity was measured. Untransfected cells served as negative controls. Luciferase readings were normalized to the DMSO control group and results were reported accordingly (Figure 8).

**Figure 8:**
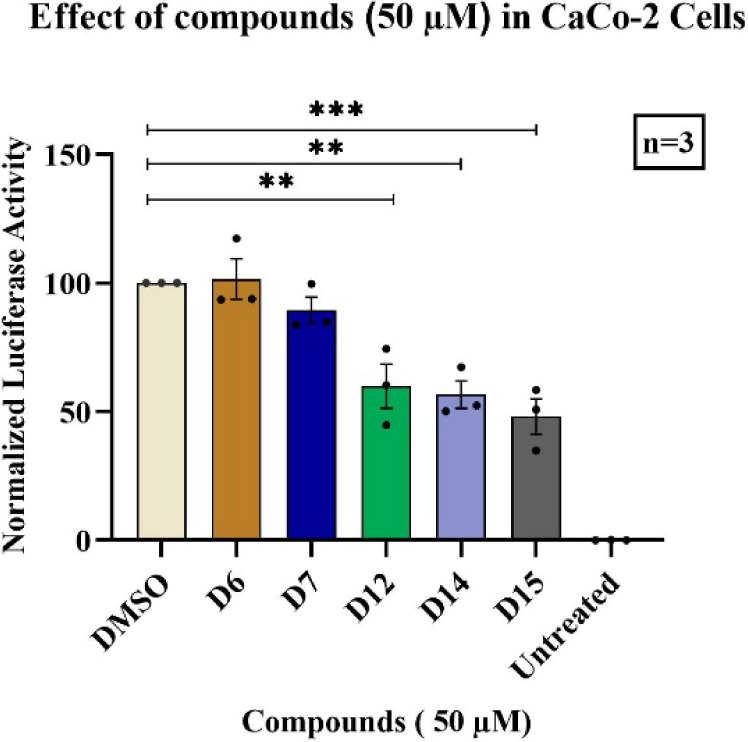
Inhibitory effects of the 5 compounds on the replication of SARS-CoV-2 subgenomic replicon in Caco-2 cells. Luciferase readings were normalized to the control group (DMSO) and set as 100. Cells that were not transfected with the SARS-CoV-2 replicon was named as Untreated group. Statistical significance was calculated for each condition compared to the control group using one-way ANOVA (nonparametric) test. Data represent mean and the standard error of the mean ± SEM, n = 4–5 (*p < 0.05; **p < 0.01; ***p < 0.0005; ****p < 0.0001.

Following the preliminary evaluation, serial dilution experiments were performed to determine the IC_50_ values for three compounds **(D12**, **D14**, and **D15**) that exhibited more than 50% inhibitory activity at 50 μM concentration. Luciferase activities were measured at concentrations of 50, 25, 12.5, and 6.25 μM for compounds **D14** and **D15**, whereas compound **D12** was tested at 100, 50, 25, and 12.5 μM concentrations (Figure 9A). The IC_50_ values of the compounds were determined by fitting the inhibitor and normalized response data to a Hill curve to the enzyme kinetic data (inhibitor vs normalized response) using GraphPad Prism software. All experiments were performed in at least three independent biological replicates, and statistical analyses were conducted. Based on the luciferase assay data, the calculated IC_50_ and 95% confidence interval (CI) values were 117.8 μM (95% CI: 86.5–188.8 μM) for **D12**, 61.2 μM (95% CI: 39.2–143.9 μM) for **D14**, and 22.2 μM (95% CI: 15.4–35.7 μM) for **D15**. These results demonstrate that compound **D15** exhibits the most potent antiviral activity in inhibiting SARS-CoV-2 replication (Figure 9B). Note that these viral replicon assays are not specific to Nsp3 (Mac1) activity, but rather assess overall viral replication. Therefore, these results do not exclude the possibility that these peptides may also bind to other viral protein targets.

**Figure 9:**
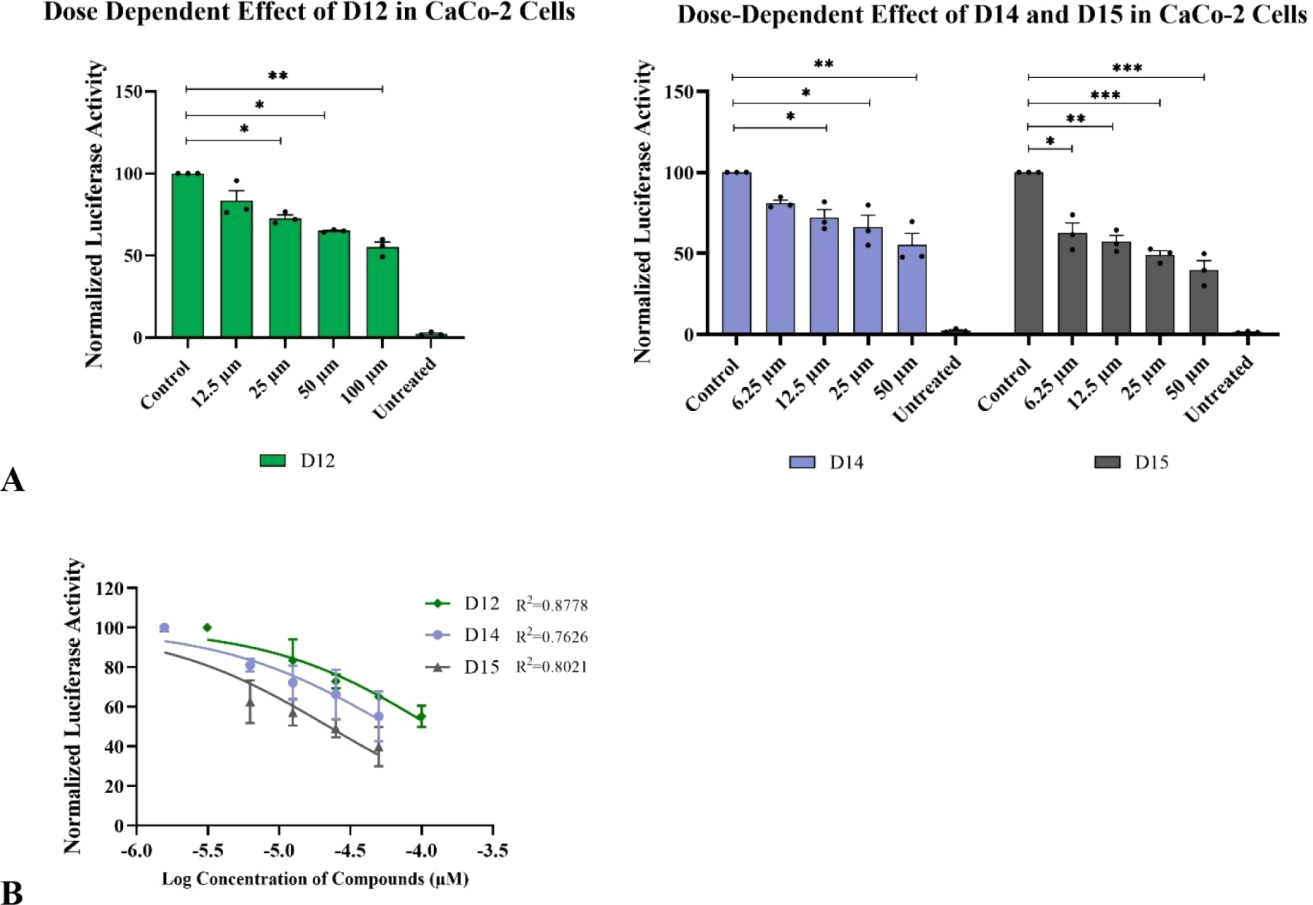
Dose-dependent inhibitory effects of compounds. (A) Compounds exhibiting >50% inhibition of SARS-CoV-2 subgenomic replicon replication in Caco-2 cells were further analyzed in a dose-dependent manner. (B) IC_50_ and 95% CI values were calculated by nonlinear regression analysis with the Hill slope set −1.0. Luciferase readings were normalized to the control group (DMSO) and set as 100. Cells that were not transfected with the SARS-CoV-2 replicon were named as Untreated group. Statistical significance was calculated for each condition compared to the control group using one-way ANOVA (nonparametric) test. Data represent mean and the standard error of the mean ± SEM, n = 4–5 (*p < 0.05; **p < 0.01; ***p < 0.0005; ****p < 0.0001.

When the biological activity profiles of the compounds were evaluated in conjunction with molecular modeling results, the histidine moiety was found to play a critical role by establishing significant interactions within the binding site. Accordingly, compounds (**D12**, **D14**, and **D15**) containing histidine exhibited higher antiviral activity, whereas **D6** and **D7** showed weaker interactions in silico studies, which were consistent with their comparatively lower antiviral experimental activities.

A more detailed comparison among **D12**, **D14**, and **D15** revealed that **D12** displayed the weakest inhibitory effect. The primary structural distinction is that **D12** contains a pyrimidine ring, whereas **D14** and **D15** incorporate more bulky and hydrophobic ring systems, namely benzofuran and methylindole, respectively. These larger ring systems appear to enhance molecular interactions within the binding pocket, thereby contributing to improved inhibitory activity.

Furthermore, the difference observed between **D14** and **D15** can be attributed to the distinct electronic and hydrogen-bonding properties of the benzofuran and methylindole moieties. In particular, the more electron-rich methylindole group, which can also act as a hydrogen-bond donor, likely facilitates stronger binding interactions, resulting in higher biological activity than the benzofuran-containing analogue.^15,26^

## 3 Conclusion

A new series of modified peptide derivatives (**D1–D15**), incorporating hydrophobic (phenylalanine and tryptophan) and positively charged (histidine) amino acid residues, were successfully designed and synthesized.

Molecular modeling studies were performed for all compounds and suggest that compounds **D13**, **D14**, and **D15** may show moderate to high binding affinity toward the Nsp3 (Mac1) active site of these three compounds. Subsequent, bioactivity assays reveal that compound **D15** shows the most potent inhibition of SARS-CoV-2 replicon replication (IC_50_ = 22.2 μM (95% CI: 15.4–35.7 μM)) and compound **D14** the second potent inhibition (IC_50_ = 61.2 μM (95% CI: 39.2–143.9 μM)). Furthermore, these compounds did not exhibit any cytotoxic effects in healthy cell lines. The biological activity results were consistent with the molecular modeling findings, and the compounds selected from the other group (**D6** and **D7**) demonstrated lower activity than the histidine-containing compounds. These observations indicate that compounds containing a histidine side chain exhibit higher biological activity, which can be rationalized with the modeling results as the histidine moiety consistently forms an interaction with the Phe132 side chain.

Altogether, these findings suggest that compounds **D14** and **D15** may be promising lead compounds for the development of peptide-based inhibitors targeting SARS-CoV-2 and potentially other coronaviruses with similar replication mechanisms.

## 4 Experimental Section

### 4.1 Materials

The chemicals and solvents were bought by Sigma-Aldrich and Merck. Melting points were determined on a STUART SMP40. ^1^H and ^13^C NMR spectra were acquired on a Varian spectrometer at 300 and at 75 Hz and Bruker spectrometer at 500 and 125 Hz, respectively. Mass spectra were obtained using Thermo Fisher Scientific LC-HRMS spectrometer. The compounds were purified by Buchi Reveleris-X2 flash column chromatography. Spectrophotometric analyses were performed by a BioTek Synergy H1 (BioTek, USA). The cell line was purchased from American Type Culture Collection (ATCC). Dulbecco’s Modified Eagle’s Medium-F12, fetal calf serum (FBS), and PBS were bought from GIBCO (Thermo Fisher Scientific, USA).

### 4.2 Methods

#### 4.2.1 General Procedures and Spectral Data

##### 4.2.1.1 (*S*)-2-Amino-*N*-methyl-3-phenylpropanamide (**A**)

To an amino ester hydrochloride (1.0 equiv) was added MeNH_2_ (33% w/w solution in EtOH, 5.0 equiv). The mixture was left to stir at room temperature for 48 h before concentrating in vacuo, dissolved in CHCl_3_ and washed with aq. sat. NaHCO_3_. The organic layer was separated, and the aqueous layer extracted 3 times with CHCl_3_. The combined organic layers were dried over MgSO_4_, filtered and concentrated in vacuo. The crude product was purified via column chromatography (gradient elution, 0% → 50% EtOAc in Hexane.^19^ Yield 78%. ^1^H NMR (CDCl_3_, 300 MHz) δ/ppm; 7.16–7.30 (m, 6H), 3.56 (dd, *J* = 9.3, 4.0 Hz, 1H), 3.23 (dd, *J* = 13.7, 4.0 Hz, 1H), 2.77 (d, *J* = 4.9 Hz, 3H), 2.60–2.67 (m, 1H); ^13^C NMR (CDCl_3_, 75 MHz) δ/ppm; 175.1, 138.1, 129.4, 128.8, 126.9, 56.6, 41.2, 26.0.

##### 4.2.1.2 (*S*)-2-Amino-3-(1*H*-indol-3-yl)-*N*-methylpropanamide (**B**)

To an amino ester hydrochloride (1.0 equiv) was added MeNH_2_ (33% w/w solution in EtOH, 5.0 equiv). The mixture was left to stir at room temperature for 48 h before concentrating in vacuo, dissolved in CHCl_3_ and washed with aq. sat. NaHCO_3_. The organic layer was separated, and the aqueous layer extracted 3 times with CHCl_3_. The combined organic layers were dried over MgSO_4_, filtered and concentrated in vacuo. The crude product was purified via column chromatography (gradient elution, 0% → 50% EtOAc in Hexane). Yield 72%. ^1^H NMR (CDCl_3_, 500 MHz) δ/ppm: 8.33 (s, 1H), 7.60 (d, *J =* 7.9 Hz, 1H), 7.31 (d, *J =* 8.1 Hz, 1H), 7.22 (s, 1H), 7.13 (t, *J =* 7.0 Hz, 1H), 7.04 (t, *J =* 7.0 Hz, 1H), 6.99 (d, *J =* 2.2 Hz, 1H), 3.65 (dd, *J =* 9.0, 4.1 Hz, 1H), 3.32 (dd, *J =* 14.5, 4.2 Hz, 1H), 2.84 (dd, *J =* 14.5, 4.2 Hz, 1H), 2.74 (d, *J =* 4.9 Hz, 3H); ^13^C NMR (CDCl_3_, 125 MHz) δ/ppm; 175.5, 136.5, 127.5, 123.0, 122.2, 119.5, 118.9, 111.8, 111.2, 55.6, 30.8, 25.8.

##### 4.2.1.3 (*S*)-2-Amino-3-(1*H*-imidazol-4-yl)-*N*-methylpropanamide (**C**)

To an amino ester hydrochloride (1.0 equiv) was added MeNH_2_ (33% w/w solution in EtOH, 5.0 equiv). The mixture was left to stir at room temperature for 48 h before concentrating in vacuo, dissolved in CHCl_3_ and washed with aq. sat. NaHCO_3_. The organic layer was separated, and the aqueous layer extracted 3 times with CHCl_3_. The combined organic layers were dried over MgSO_4_, filtered and concentrated in vacuo. The crude product was purified via column chromatography (gradient elution, 0% → 100% EtOAc in Hexane). The analytical data were matched with the previously reported data. Yield 68%. ^1^H NMR (CDCl_3_, 500 MHz) δ/ppm; 7.53 (s, 1H), 7.48 (d, *J =* 0.8 Hz, 1H), 3.55–3.52 (m, 1H), 2.97 (dd, *J =* 14.6, 4.2 Hz, 1H), 2.85–2.80 (m, 1H), 2.72 (d, *J =* 4.9 Hz, 3H); ^13^C NMR (DMSO-*d*_6_, 125 MHz) δ/ppm; 175.3, 135.4, 118.1, 118.0, 55.8, 33.1, 26.1.

#### 4.2.2 Synthesis of **D** Series

To a stirred solution of **A–C** (1,5 mmol, 1.0 equiv) in DMF (10 mL) was added carboxylic acid derivatives (0.04 g, 1,8 mmol, 1.2 equiv), DCC (1,65 mmol, 1.1 equiv), HOBt (1,5 mmol, 1 equiv), and DIPEA (3 mmol, 2 equiv) at 25 °C. After completion of the reaction, its solvent was evaporated by rotary evaporation and concentrated under reduced pressure. Then, the reaction mixture was diluted with EtOAc, quenched with saturated NaHCO_3_ solution, and extracted with EtOAc (2 × 100 mL). The combined organic extracts were dried over anhydrous Na_2_SO_4_, filtered, and concentrated under reduced pressure. The crude compound was purified by column chromatography over silica gel (0 → 20% MeOH in EtOAc) to afford the pure product.^20^

##### 4.2.2.1 (*S*)-*N*-(1-(Methylamino)-1-oxo-3-phenylpropan-2-yl)­picolinamide (**D1**)

White powder, yield 70%, 122–124 °C mp.; IR; 3403, 3296, 1683, 1505, 1453, 1324, 1187, 997, 898, 537 cm^–1^; ^1^H NMR (DMSO-*d*_6_, 300 MHz) δ/ppm: 8.72–8.62 (m, 2H), 8.15 (d, *J* = 2.7 Hz, 1H), 8.04–7.95 (m, 2H), 7.66–7.56 (m, 1H), 7.29–7.13 (m, 5H), 4.75–4.72 (m, 1H), 3.12–3.06 (m, 2H), 2.62 (dd, *J* = 4.5, 1.7 Hz, 3H); ^13^C NMR (DMSO-*d*_6_, 75 MHz) δ/ppm; 171.4, 163.7, 149.9, 149.1, 138.5, 138.1, 129.8, 128.7, 127.4, 127.0, 122.4, 54.5, 38.6, 26.2. HRMS (*m*/*z*) [M – H]^+^ calcd. for C_16_H_16_N_3_O_2_, 282.1242, found 282.1251.

##### 4.2.2.2 (*S*)-*N*-(1-(Methylamino)-1-oxo-3-phenylpropan-2-yl)­pyrimidine-2-carboxamide (**D2**)

White powder, yield 60%, 144–146 °C mp.; IR; 3278, 1661, 1510, 1408, 1160, 839, 500 cm^–1^; ^1^H NMR (DMSO-*d*_6_, 300 MHz) δ/ppm: 8.96 (dd, *J* = 4.9, 1.4 Hz, 2H), 8.79 (d, *J* = 8.5 Hz, 1H), 8.13 (d, *J* = 3.8 Hz, 1H), 7.68 (td, *J* = 4.9, 1.5 Hz, 1H), 7.27–7.13 (m, 5H), 4.74–4.67 (m, 1H), 3.18–2.99 (m, 2H), 2.61 (d, *J* = 4.2 Hz, 3H); ^13^C NMR (DMSO-*d*_6_, 75 MHz) δ/ppm; 171.3, 162.2, 158.3, 157.9, 138.2, 129.8, 128.7, 127.4, 127.0, 123.8, 54.9, 38.3, 26.2. HRMS (*m*/*z*) [M – H]^+^ calcd. for C_15_H_15_N_4_O_2_, 283.1195, found 283.1209.

##### 4.2.2.3 (*S*)-4-Methyl-*N*-(1-(methylamino)-1-oxo-3-phenylpropan-2-yl)­thiophene-2-carboxamide (**D3**)

White powder, yield 60%, 182–184 °C mp.; IR; 3286, 2931, 1666, 1529, 1454, 1317, 1228, 862, 694, 476 cm^–1^; ^1^H NMR (CDCl_3_, 500 MHz) δ/ppm: 7.24 (s, 1H), 7.18–7.12 (m, 5H), 7.03 (d, *J* = 7.6 Hz, 1H), 6.97 (s, 1H), 6.43 (s, 1H), 4.78 (q, *J* = 7.5 Hz, 1H), 3.14 (dd, *J* = 13.6, 6.6 Hz, 1H), 3.06 (dd, *J* = 13.6, 7.6 Hz, 1H), 2.64 (d, *J* = 4.8 Hz, 3H), 2.14 (s, 3H); ^13^C NMR (CDCl_3_, 125 MHz) δ/ppm; 171.6, 161.9, 138.5, 137.7, 136.8, 130.6, 129.3, 128.6, 126.9, 126.1, 55.0, 38.6, 26.1, 15.6. HRMS (*m*/*z*) [M – H]^+^ calcd. for C_16_H_17_N_2_O_2_S, 301.1010, found 301.1020.

##### 4.2.2.4 (*S*)-*N*-(1-(Methylamino)-1-oxo-3-phenylpropan-2-yl)­benzofuran-2-carboxamide (**D4**)

White powder, yield 40%, 178–180 °C mp.; IR; 3321, 1638, 1595, 1518, 1447, 1298, 1174, 1075, 840, 528 cm^–1^; ^1^H NMR (CDCl_3_, 300 MHz) δ/ppm: 7.58 (d, *J* = 7.7 Hz, 1H), 7.43 (d, *J* = 8.2 Hz, 1H), 7.40–7.30 (m, 3H), 7.26–7.16 (m, 6H), 5.92 (s, 1H), 4.76 (dd, *J* = 14.4, 7.9 Hz, 1H), 3.18 (dd, *J* = 13.3, 5.9 Hz, 1H), 3.08 (dd, *J* = 13.3, 7.9 Hz, 1H), 2.66 (d, *J* = 4.7 Hz, 3H); ^13^C NMR (CDCl_3_, 75 MHz) δ/ppm; 169.9, 157.6, 153.8, 146.8, 135.4, 128.2, 128.1, 127.6, 126.3, 126.1, 126.0, 122.7, 121.6, 110.9, 109.9, 53.6, 37.6, 25.2. HRMS (*m*/*z*) [M – H]^+^ calcd. for C_19_H_17_N_2_O_3_, 321.1239, found 321.1248.

##### 4.2.2.5 (*S*)-1-Methyl-*N*-(1-(methylamino)-1-oxo-3-phenylpropan-2-yl)-1*H*-indole-3-carboxamide (**D5**)

White powder, yield 77%, 208–210 °C mp.; IR; 3315, 3280, 2926, 2849, 1652, 1557, 1414, 1336, 1185, 1080, 744, 527 cm^–1^; ^1^H NMR (DMSO-*d*_6_, 500 MHz) δ/ppm: 8.10 (s, 1H), 8.03 (d, *J* = 7.9 Hz, 1H), 7.98–7.95 (m, 2H), 7.47 (d, *J* = 8.2 Hz, 1H), 7.32 (d, *J* = 7.3 Hz, 2H), 7.24 (t, *J* = 7.6 Hz, 2H), 7.19 (t, *J* = 7.1 Hz, 1H), 7.14 (t, *J* = 7.3 Hz, 1H), 7.11 (t, *J* = 7.9 Hz, 1H), 4.68–4.64 (m, 1H), 3.82 (s, 3H), 3.08 (dd, *J* = 13.7, 4.5 Hz, 1H), 2.95 (dd, *J* = 13.7, 4.6 Hz, 1H), 2.61 (d, *J* = 4.6 Hz, 3H); ^13^C NMR (DMSO-*d*_6_, 75 MHz) δ/ppm; 172.8, 164.6, 157.2, 139.2, 137.2, 132.5, 129.8, 128.7, 127.1, 126.8, 122.5, 121.2, 110.8, 109.7, 54.7, 34.0, 26.0, 25.2. HRMS (*m*/*z*) [M – H]^+^ calcd. for C_20_H_20_N_3_O_2_, 334.1555, found 334.1564.

##### 4.2.2.6 (*S*)-*N*-(3-(1*H*-Indol-3-yl)-1-(methylamino)-1-oxopropan-2-yl)­picolinamide (**D6**)

Light yellow powder, yield 43%, 155–157 °C mp.; IR; 3262, 1650, 1518, 1461, 1235, 1100, 745, 555, 427 cm^–1^; ^1^H NMR (CDCl_3_, 500 MHz) δ/ppm: 8.61 (d, *J =* 8.1 Hz, 1H), 8.53 (s, 1H), 8.41 (d, *J =* 4.7 Hz, 1H), 8.04 (d, *J =* 7.7 Hz, 1H), 7.72 (dt, *J =* 7.7, 1.5 Hz, 1H), 7.62 (d, *J =* 7.9 Hz, 1H), 7.34–7.28 (m, 1H), 7.25 (d, *J =* 8.1 Hz, 1H), 7.07 (t, *J =* 7.5 Hz, 1H), 7.01–6.98 (m, 2H), 5.94 (d, *J =* 4.6 Hz, 1H), 4.80 (dd, *J =* 13.6, 7.7 Hz, 1H), 3.35 (dd, *J =* 14.5, 5.6 Hz, 1H), 3.22 (dd, *J =* 14.5, 7.6 Hz, 1H), 2.54 (d, *J =* 4.8 Hz, 3H); ^13^C NMR (CDCl_3_, 125 MHz) δ/ppm: 171.8, 164.5, 149.2, 148.3, 137.3, 136.2, 127.3, 126.4, 123.3, 122.1, 122.1, 119.5, 119.0, 111.2, 110.6, 54.1, 28.3, 26.2. HRMS (*m*/*z*) [M – H]^+^ calcd. for C_18_H_18_N_4_O_2_, 321.1351, found 321.1363.

##### 4.2.2.7 (*S*)-*N*-(3-(1*H*-Indol-3-yl)-1-(methylamino)-1-oxopropan-2-yl)­pyrimidine-2-carboxamide (**D7**)

White powder, yield 30%, 233–236 °C mp.; IR; 3291, 1686, 1645, 1512, 1454, 1407, 1092, 724, 632 cm^–1^; ^1^H NMR (DMSO-*d*_6_, 500 MHz) δ/ppm: 10.70 (s, 1H), 8.81 (d, *J* = 4.9 Hz, 2H), 8.58 (d, *J* = 8.3 Hz, 1H), 7.99 (q, *J* = 4.4 Hz, 1H), 7.54 (t, *J* = 4.9 Hz, 1H), 7.42 (d, *J* = 7.9 Hz, 1H), 7.18 (d, *J* = 8.1 Hz, 1H), 6.99 (d, *J* = 2.3 Hz, 1H), 6.90 (t, *J* = 7.9 Hz, 1H), 6.80 (t, *J* = 7.9 Hz, 1H), 4.61–4.57 (m, 1H), 3.10–3.08 (m, 2H), 2.46 (d, *J* = 4.6 Hz, 3H); ^13^C NMR (DMSO-*d*_6_, 75 MHz) δ/ppm; 171.7, 162.1, 158.3, 157.9, 136.7, 128.0, 124.2, 121.5, 119.1, 118.8, 111.9, 110.2, 54.3, 28.6, 26.3. HRMS (*m*/*z*) [M – H]^+^ calcd. for C_17_H_16_N_5_O_2_, 322.1304, found 322.1313.

##### 4.2.2.8 (*S*)-*N*-(3-(1*H*-Indol-3-yl)-1-(methylamino)-1-oxopropan-2-yl)-4-methylthiophene-2-carboxamide (**D8**)

Yellow powder, yield 27%, 248–250 °C mp.; IR; 3282, 3082, 2921, 1617, 1553, 1506, 1226, 739, 424 cm^–1^; ^1^H NMR (CDCl_3_, 500 MHz) δ/ppm: 8.46 (s, 1H), 7.60 (d, *J* = 7.9 Hz, 1H), 7.25 (d, *J* = 8.1 Hz, 1H), 7.13–7.05 (m, 2H), 7.04–6.96 (m, 2H), 6.94 (s, 2H), 6.17 (q, *J* = 4.5 Hz, 1H), 4.80–4.76 (m, 1H), 3.32 (dd, *J* = 14.5, 5.6 Hz, 1H), 3.15 (dd, *J* = 14.5, 7.7 Hz, 1H), 2.55 (d, *J* = 4.8 Hz, 3H), 2.10 (s, 3H); ^13^C NMR (CDCl_3_, 125 MHz) δ/ppm; 172.1, 162.0, 138.5, 137.7, 136.2, 130.6, 127.4, 126.2, 123.5, 122.1, 119.6, 118.8, 111.4, 110.5, 54.4, 28.3, 26.2, 21.1. HRMS (*m*/*z*) [M – H]^+^ calcd. for C_18_H_18_N_3_O_2_S, 340.1119, found 340.1130.

##### 4.2.2.9 (*S*)-*N*-(3-(1*H*-Indol-3-yl)-1-(methylamino)-1-oxopropan-2-yl)­benzofuran-2-carboxamide (**D9**)

White powder, yield 44%, 192–194 °C mp.; IR; 3291, 1651, 1597, 1504, 1445, 1299, 1255, 735, 424 cm^–1^; ^1^H NMR (CDCl_3_, 300 MHz) δ/ppm: 9.65 (s, 1H), 7.58–7.40 (m, 3H), 7.32–7.16 (m, 4H), 7.11 (t, *J* = 7.4 Hz, 1H), 7.01–6.86 (m, 3H), 6.81–6.76 (m, 1H), 4.83–4.68 (m, 1H), 3.25 (dd, *J* = 14.5, 5.8 Hz, 1H), 3.15 (dd, *J* = 14.5, 7.1 Hz, 1H), 2.51 (d, *J* = 4.7 Hz, 3H); ^13^C NMR (CDCl_3_+DMSO-*d*_6_, 75 MHz) δ/ppm; 171.7, 158.6, 154.8, 148.5, 136.5, 127.6, 127.4, 127.0, 123.8, 123.6, 122.7, 121.7, 119.2, 118.8, 111.9, 111.6, 110.5, 110.0, 54.0, 28.8, 26.3. HRMS (*m*/*z*) [M – H]^+^ calcd. for C_21_H_18_N_3_O_3_, 360.1348, found 360.1358.

##### 4.2.2.10 (*S*)-*N*-(3-(1*H*-Indol-3-yl)-1-(methylamino)-1-oxopropan-2-yl)-1-methyl-1*H*-indole-3-carboxamide (**D10**)

White powder, yield 56%, 214–216 °C mp.; IR; 3397, 3300, 3273, 1651, 1556, 1459, 1222, 766, 423 cm^–1^; ^1^H NMR (DMSO-*d*_6_, 500 MHz) δ/ppm: 10.76 (s, 1H), 8.09 (s, 1H), 8.03 (d, *J* = 7.9 Hz, 1H), 7.97 (q, *J* = 4.5 Hz, 1H), 7.83 (d, *J* = 8.2 Hz, 1H), 7.65 (d, *J* = 7.9 Hz, 1H), 7.46 (d, *J* = 8.2 Hz, 1H), 7.30 (d, *J* = 8.0 Hz, 1H), 7.22–7.16 (m, 2H), 7.10 (t, *J* = 7.9 Hz, 1H), 7.04 (t, *J* = 7.1 Hz, 1H), 6.97 (t, *J* = 7.9 Hz, 1H), 4.73–4.69 (m, 1H), 3.81 (s, 3H), 3.21 (dd, *J* = 14.6, 4.8 Hz, 1H), 3.09 (dd, *J* = 14.6, 9.3 Hz, 1H), 2.61 (d, *J* = 4.6 Hz, 3H); ^13^C NMR (DMSO-*d*_6_, 125 MHz) δ/ppm; 173.0, 164.4, 137.1, 136.5, 132.7, 127.8, 126.8, 123.9, 122.3, 121.4, 121.3, 121.0, 118.9, 118.6, 111.7, 111.1, 110.6, 109.6, 53.7, 33.4, 28.3, 26.1. HRMS (*m*/*z*) [M – H]^+^ calcd. for C_22_H_21_N_4_O_2_, 373.1664, found 373.1674.

##### 4.2.2.11 (*S*)-*N*-(3-(1*H*-Imidazol-4-yl)-1-(methylamino)-1-oxopropan-2-yl)­picolinamide (**D11**)

Yellow powder, yield 52%, 176–178 °C mp.; IR; 3362, 2901, 1644, 1510, 1276, 1158, 822, 604, 540 cm^–1^; ^1^H NMR (CDCl_3_, 500 MHz) δ/ppm: 8.82 (s, 1H), 8.51 (d, *J* = 4.1 Hz, 1H), 8.07 (d, *J* = 7.8 Hz, 1H), 7.78 (td, *J* = 7.7, 1.7 Hz, 1H), 7.54 (s, 1H), 7.39–7.36 (m, 1H), 6.93 (s, 1H), 6.86 (s, 1H), 4.84–4.80 (m, 1H), 3.26 (dd, *J* = 14.9, 4.4 Hz, 1H), 3.04 (dd, *J* = 14.9, 6.6 Hz, 1H), 2.71 (d, *J* = 4.8 Hz, 3H); ^13^C NMR (CDCl_3_, 125 MHz) δ/ppm; 171.9, 164.9, 149.1, 148.5, 137.3, 135.2, 126.6, 122.3, 52.7, 28.3, 26.3. HRMS (*m*/*z*) [M – H]^+^ calcd. for C_13_H_14_N_5_O_2_, 272.1147, found 272.1178.

##### 4.2.2.12 (*S*)-*N*-(3-(1*H*-Imidazol-4-yl)-1-(methylamino)-1-oxopropan-2-yl)­pyrimidine-2-carboxamide (**D12**)

White powder, yield 60%, 196–198 °C mp.; IR; 3249, 3159, 3132, 3046, 2962, 1682, 1651, 1568, 1408, 1154, 832, 675, 480 cm^–1^; ^1^H NMR (CD_3_OD, 500 MHz) δ/ppm: 8.91 (d, *J =* 4.9 Hz, 2H), 7.61 (t, *J =* 4.9 Hz, 1H), 7.58 (d, *J =* 1.0 Hz, 1H), 6.88 (s, 1H), 4.82 (dd, *J =* 7.7, 5.6 Hz, 1H), 3.20 (dd, *J =* 14.7, 5.5 Hz, 1H), 3.13 (dd, *J =* 14.8, 7.7 Hz, 1H), 2.72 (d, *J =* 4.0 Hz, 3H); ^13^C NMR (CD_3_OD, 125 MHz) δ/ppm: 172.0, 162.8, 157.5, 156.8, 135.0, 123.1, 53.8, 29.4, 25.1. HRMS (*m*/*z*) [M + H]^+^ calcd. for C_12_H_15_N_6_O_2_, 275.1256, found 275.1178.

##### 4.2.2.13 (*S*)-*N*-(3-(1*H*-Imidazol-4-yl)-1-(methylamino)-1-oxopropan-2-yl)-4-methylthiophene-2-carboxamide (**D13**)

White powder, yield 40%, 187–189 °C mp.; IR; 3285, 1657, 1628, 1555, 1429, 1317, 1222, 1083, 741, 615, 422 cm^–1^; ^1^H NMR (CD_3_OD, 500 MHz) δ/ppm: 7.86 (s, 1H), 7.53 (s, 1H), 7.22 (s, 1H), 6.96 (s, 1H), 4.74 (dd, *J =* 8.0, 6.1 Hz, 1H), 3.20 (dd, *J =* 14.9, 5.6 Hz, 1H), 3.06 (dd, *J =* 14.9, 8.8 Hz, 1H), 2.24 (s, 3H), 2.71 (s, 3H); ^13^C NMR (CD_3_OD, 125 MHz) δ/ppm: 172.4, 162.9, 138.3, 137.5, 134.6, 132.8, 130.8, 126.3, 116.6, 53.6, 28.5, 25.0, 14.1. HRMS (*m*/*z*) [M + H]^+^ calcd. for C_13_H_17_N_4_O_2_S, 293.1072, found 293.1062.

##### 4.2.2.14 (*S*)-*N*-(3-(1*H*-Imidazol-4-yl)-1-(methylamino)-1-oxopropan-2-yl)­benzofuran-2-carboxamide (**D14**)

White powder, yield 32%, 219–221 °C mp.; IR; 3296, 3237, 2931, 1638, 1598, 1535, 1410, 1257, 1184, 1086, 743, 616, 417 cm^–1^; ^1^H NMR (DMSO-*d*_6_, 300 MHz) δ/ppm: 8.92 (s, 1H), 8.13 (s, 1H), 7.75 (d, *J* = 7.8 Hz, 1H), 7.65 (d, *J* = 8.3 Hz, 1H), 7.58 (s, 1H), 7.52 (s, 1H), 7.44 (t, *J* = 7.3 Hz, 1H), 7.30 (t, *J* = 7.7 Hz, 1H), 6.77 (s, 1H), 4.68–4.52 (m, 1H), 3.00 (d, *J* = 6.4 Hz, 2H), 2.55 (d, *J* = 3.2 Hz, 3H); ^13^C NMR (DMSO-*d*_6_, 75 MHz) δ/ppm; 171.7, 158.6, 154.8, 149.4, 135.4, 127.7, 127.5, 124.3, 123.4, 112.5, 110.4, 52.9, 31.0, 26.3. HRMS (*m*/*z*) [M + H]^+^ calcd. for C_16_H_17_N_4_O_3_, 313.1300, found 313.1288.

##### 4.2.2.15 (*S*)-*N*-(3-(1*H*-Imidazol-4-yl)-1-(methylamino)-1-oxopropan-2-yl)-1-methyl-1*H*-indole-3-carboxamide (**D15**)

White powder, yield 54%, 224–226 °C mp.; IR; 3327, 3183, 2922, 1660, 1625, 1570, 1428, 1321, 1183, 776, 643, 408 cm^–1^; ^1^H NMR (CD_3_OD, 500 MHz) δ/ppm: 8.01 (d, *J =* 7.9 Hz, 1H), 7.84 (s, 1H), 7.59 (d, *J =* 1.0 Hz, 1H), 7.39 (d, *J =* 8.2 Hz, 1H), 7.25–7.20 (m, 1H), 7.18–7.13 (m, 1H), 6.90 (s, 1H), 4.81–4.76 (m, 1H), 3.80 (s, 3H), 3.18 (dd, *J =* 14.8, 5.5 Hz, 1H), 3.08 (dd, *J =* 14.8, 8.3 Hz, 1H), 2.72 (s, 3H); ^13^C NMR (CD_3_OD, 125 MHz) δ/ppm: 173.3, 166.2, 137.3, 134.9, 132.2, 126.2, 122.2, 120.9, 120.4, 109.5, 108.9, 53.5, 32.0, 29.3, 25.0. HRMS (*m*/*z*) [M + H]^+^ calcd. for C_17_H_20_N_5_O_2_, 326.1607, found 326.1606.

### 4.3 Molecular Docking Studies

All molecular modeling calculations were carried out using the Schrödinger software package (v2021-1, Schrödinger, LLC, New York, NY).

#### 4.3.1 Ligand Preparation

The three-dimensional geometries of the compounds in our in-house library were generated with the *3D Builder* module. All molecules were built as S-enantiomers. Possible ionization and tautomeric states at physiological pH (7.0 ± 2.0) were enumerated with the *LigPrep* module, and the resulting structures were energy-minimized using the OPLS4 force field.

#### 4.3.2 Protein Preparation

The crystal structure of SARS-CoV-2 Nsp3 (PDB ID: 7KQP) was retrieved from the RCSB Protein Data Bank and prepared for subsequent docking studies using the *Protein Preparation Workflow* module.^27–29^ In this step, crystallographic water molecules and ions were removed. Only the primary protein chain was kept. Hydrogen atoms were added and N- and C-terminals were capped with N-acetyl and *N*-methylamide groups, respectively, and the structure was energy minimized and the OPLS4 force field.^30^

#### 4.3.3 Molecular Docking

A grid box was generated at the centroid of the cocrystallized ligand. Ligands with lengths of ≤20 Å were allowed to dock. Free rotation was allowed for hydroxyl groups within 5 Å of the cocrystallized ligands that were not involved in the protein hydrogen bonding network. Docking studies were conducted using the Glide module with Standard Precision (SP) settings.^31^ Each ligand was docked 25 times to produce the three best poses. Retrospective docking studies on the cocrystallized ligand were performed to validate the procedure. For each ligand, the top three ranked poses were retained for visual inspection and further analysis.

#### 4.3.4 Molecular Dynamics Simulations

Molecular dynamics (MD) simulations were carried out using the *Desmond* module.^32^ Each protein–ligand complex was placed in an orthorhombic simulation box with periodic boundary conditions, solvated with TIP5P water molecules, and neutralized with Na+ or Cl– counterions. In addition, 0.15 M NaCl was added to mimic physiological conditions. Initial minimization was performed under positional restrains on protein and ligand for 100 ps, followed by unrestrained production runs of 250 ns a t 300 K and 1 bar using the Nosé–Hoover chain thermostat and Martyna–Tobias–Klein barostat, respectively. Trajectory analyses included calculation of protein Cα and ligand RMSD binding interactions and MM/GBSA binding free energies.

### 4.4 SARS-CoV-2 Inhibitory Activity

Experimental protocols are similar to previous studies.^22,23^

#### 4.4.1 Cytotoxicity Assay

Cell viability in Caco-2 (**D6–D7**, **D12**, **D14–D15**) and CCD1079Sk (**D1–D15**) were determined by MTT assay. 8,000 cells were seeded per well in a 96-well plate and allowed to adhere overnight. The following day, cells were treated with the compounds at the indicated concentrations for 24 h. Following treatment with the indicated compounds, MTT reagent (3-(4,5-dimethylthiazol-2-yl)-2,5-diphenyltetrazolium bromide) was added (1 mg/mL) and incubated for 4 h at 37 °C. Formazan crystals were dissolved in DMSO:ethanol (1:1, v/v) mixture, and absorbance was measured at 600 nm. Cells treated with 5% DMSO served as negative controls. All experiments were performed with biological triplicates and repeated independently at least three times (*n* > 3). Statistical analysis was conducted using a two-tailed Student’s *t*-test to compare compound-treated groups with vehicle controls.

Cell lines: Caco-2 (human colorectal adenocarcinoma; ATCC HTB-37); Huh7 (human hepatocellular; kindly provided by Ege University, Department of Bioengineering, Animal Cell Culture Collection).

#### 4.4.2 SARS-CoV-2 Subgenomic Replicon Assay

The single-cycle infectious viral replicon particle encodes dual reporters—*Gaussia* luciferase (Luc) and green fluorescent protein (GFP)—which enable quantitative and visual assessment of viral replication under BSL-2 conditions.^22,23^

Infectious viral replicon particles [ΔS-VRP­(G)] were produced by cotransfecting the ΔS-Luc-GFP bacmid and VSV-G expression plasmid into Huh7 cells. Large-scale purification of the ΔS-Luc-GFP bacmid DNA was performed using the QIAGEN Large-Construct Kit (Cat. No. 12462) according to the manufacturer’s instructions.

#### 4.4.3 Rescue and Amplification of ΔS-VRP­(G) in Mammalian Cells

Huh-7 cells were seeded in 6-well plates (4 × 10^5^ cells/well) 24 h prior to transfection. At 70–80% confluency, cells were cotransfected with 3.5 μg ΔS-Luc-GFP bacmid and 0.5 μg VSV-G plasmid using Lipofectamine 3000 according to the manufacturer’s protocol. Transfection efficiency was monitored using pcDNA (3.0 μg) and eGFP (0.5 μg) as positive controls. After 5-h incubation, the transfection medium was replaced with DMEM containing 2% heat-inactivated FBS. GFP expression and luciferase activity were monitored to assess viral spread. At 48–72 h post-transfection, cells underwent a second transfection with 2 μg VSV-G plasmid.

For antiviral assays, Caco-2 (1.2 × 10^5^ cells/well in 12-well) cells were seeded 24 h before infection. Cells were infected with ΔS-VRP­(G) (500 μL/well) for 2–3 h, washed twice with PBS, and treated with test compounds diluted in DMEM (2% FBS). At 16–18 h postinfection, Nano-Luc luciferase activity was measured using the Biotek Cytation 5 system following addition of Coelenterazine h substrate (1:400 dilution in Renilla Salt solution; 50 μL/well; NanoLight Technology, Cat. #301–500). Data were normalized to DMSO-treated controls and analyzed using GraphPad Prism software.

## Supplementary Material



## Notes

^1^H, ^13^C NMR and MS spectra of synthesized compounds; viability (%)–log concentration (μM) curves for the cytotoxic effects (PDF)
