# Supplementary material for: Synthesis and Biological Evaluation of Modified Peptide Derivatives Targeting the SARS-CoV‑2 Nsp3 Macrodomain (Mac1) Replication Domain
Source: ACS Omega. 2026 Apr 24;11(18):27468–80. doi: 10.1021/acsomega.6c02033 (PMC13177230; doi:10.1021/acsomega.6c02033)
Supplement: Supplementary file 1 [file ao6c02033_si_001.pdf]

Supplementary Information of Paper

Synthesis, and Biological Evaluation of Modified Peptide Derivatives Targeting the SARS-CoV-2 Nsp3 macrodomain (Mac1) Replication Domain

Özge Özmen[1,2], Betül Oruçoğlu[3], Serap İpek Dingiş Birgül[4], Dilek Öztürk Civelek[5,6], Şeref Gül[3], Atilla Akdemir[7], Fatih Sönmez[8], Belma Zengin Kurt[4,*]

[1]*Bezmialem Vakif University, Faculty of Pharmacy, 34093, Istanbul, Türkiye*

[2]*Kartal Dr. Lutfi Kirdar City Hospital, 34865, Istanbul, Türkiye*

[3]*Institute of Life Sciences and Biotechnology, Bezmialem Vakif University, 34820, Istanbul, Türkiye*

[4]*Department of Pharmaceutical Chemistry, Faculty of Pharmacy, Bezmialem Vakif University, 34093, Istanbul, Türkiye*

[5]*Department of Pharmacology, Faculty of Pharmacy, Istanbul University -Cerrahpaşa, 34500, Istanbul, Türkiye*

[6]*Department of Pharmacology, Faculty of Pharmacy, Bezmialem Vakif University, 34093, Istanbul, Türkiye*

[7]*Department of Pharmacology, Faculty of Pharmacy, Istanbul Kent University, 34406, Istanbul, Türkiye*

[8]*Pamukova Vocational School, Sakarya University of Applied Sciences, 54055, Sakarya, Türkiye*

*Corresponding author: Belma Zengin Kurt; e-mail: bzengin@bezmialem.edu.tr

Table of Content

IC_{50} curves of Cytotoxicity of CCD cell line S3

Toxicity Analysis of Compounds in Caco-2 Cells S2

^{1}H NMR, ^{13}C NMR, and MS spectrums of compounds S4

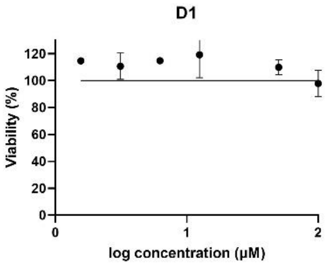
D1
120
100
80
60
40
20
0
Viability (%)
0
1
2
log concentration (μM)

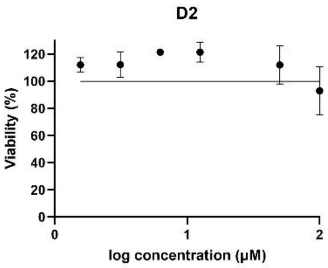
D2
120
100
80
60
40
20
0
Viability (%)
0
1
2
log concentration (μM)

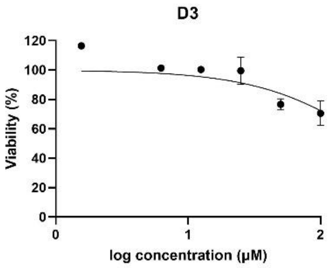
D3
120
100
80
60
40
20
0
Viability (%)
0
1
2
log concentration (μM)

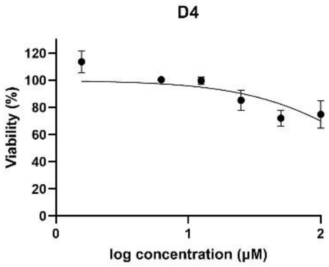
D4
120
100
80
60
40
20
0
Viability (%)
0
1
2
log concentration (μM)

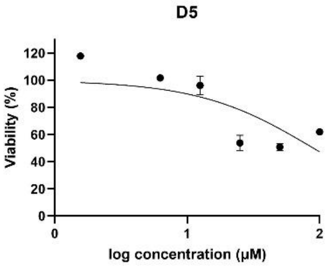
D5
120
100
80
60
40
20
0
Viability (%)
0
1
2
log concentration (μM)

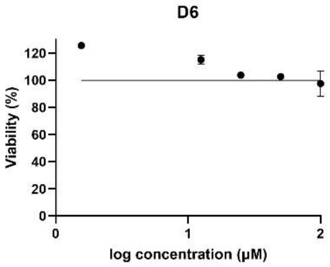
D6
120
100
80
60
40
20
0
Viability (%)
0
1
2
log concentration (μM)

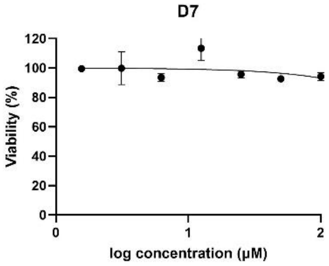
D7
120
100
80
60
40
20
0
Viability (%)
0
1
2
log concentration (μM)

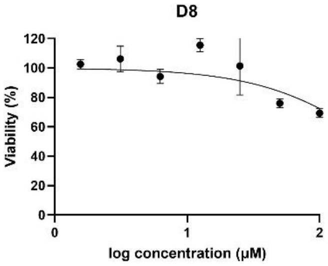
D8
120
100
80
60
40
20
0
Viability (%)
0
1
2
log concentration (μM)

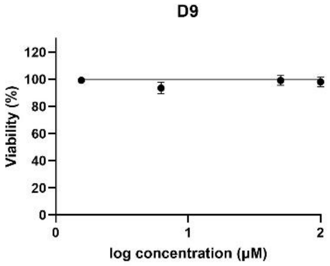
D9
120
100
80
60
40
20
0
Viability (%)
0
1
2
log concentration (μM)

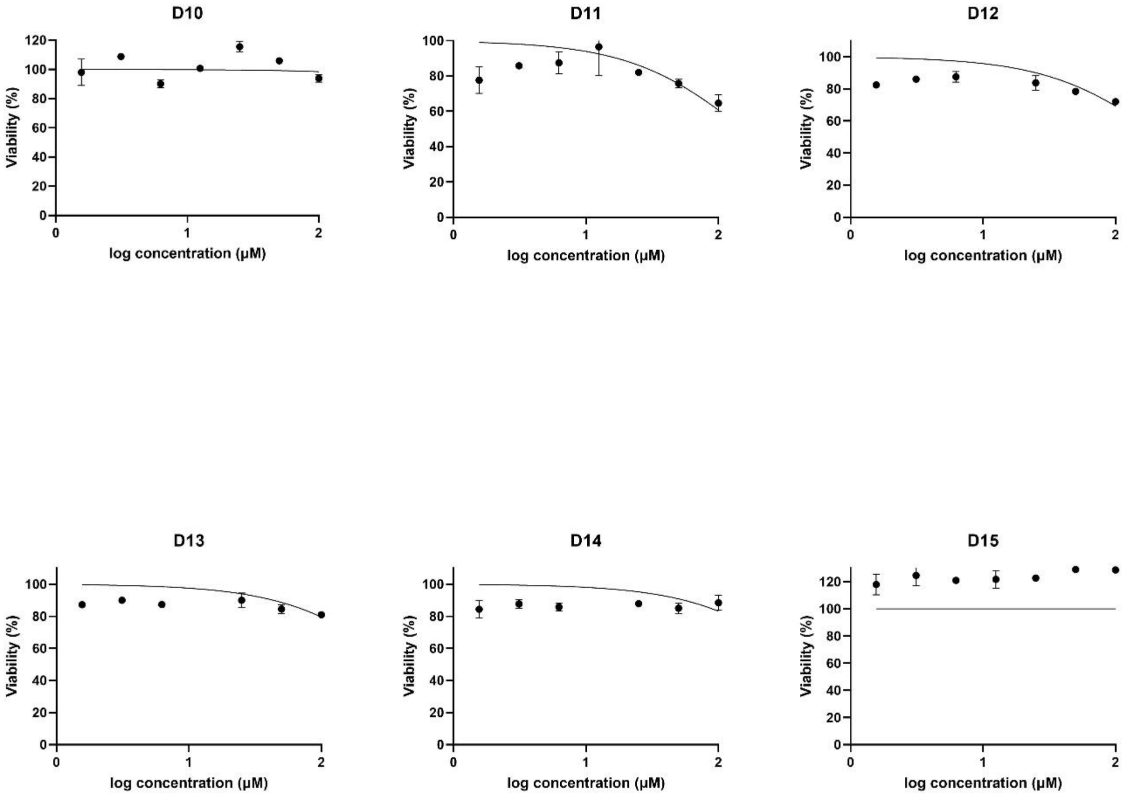

Figure S1. IC_{50} curves of **D1-D15** on CCD

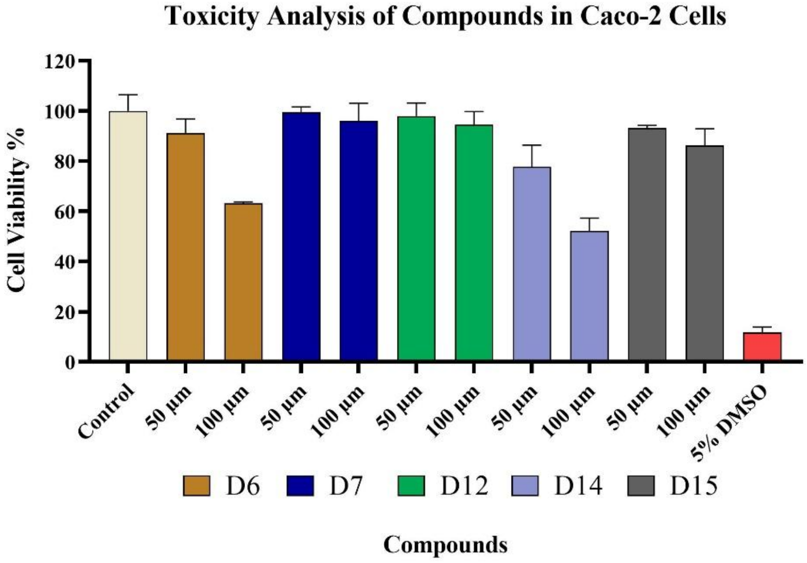

Figure S2. The cytotoxic effects of **D6, D7, D12, D14, D15** compounds on Caco-2 cells were evaluated at concentrations of 50 µM and 100 µM. *Cell viability was measured and normalized to control values (set as 100%). Data are presented as mean ± SEM of three independent biological replicates.*

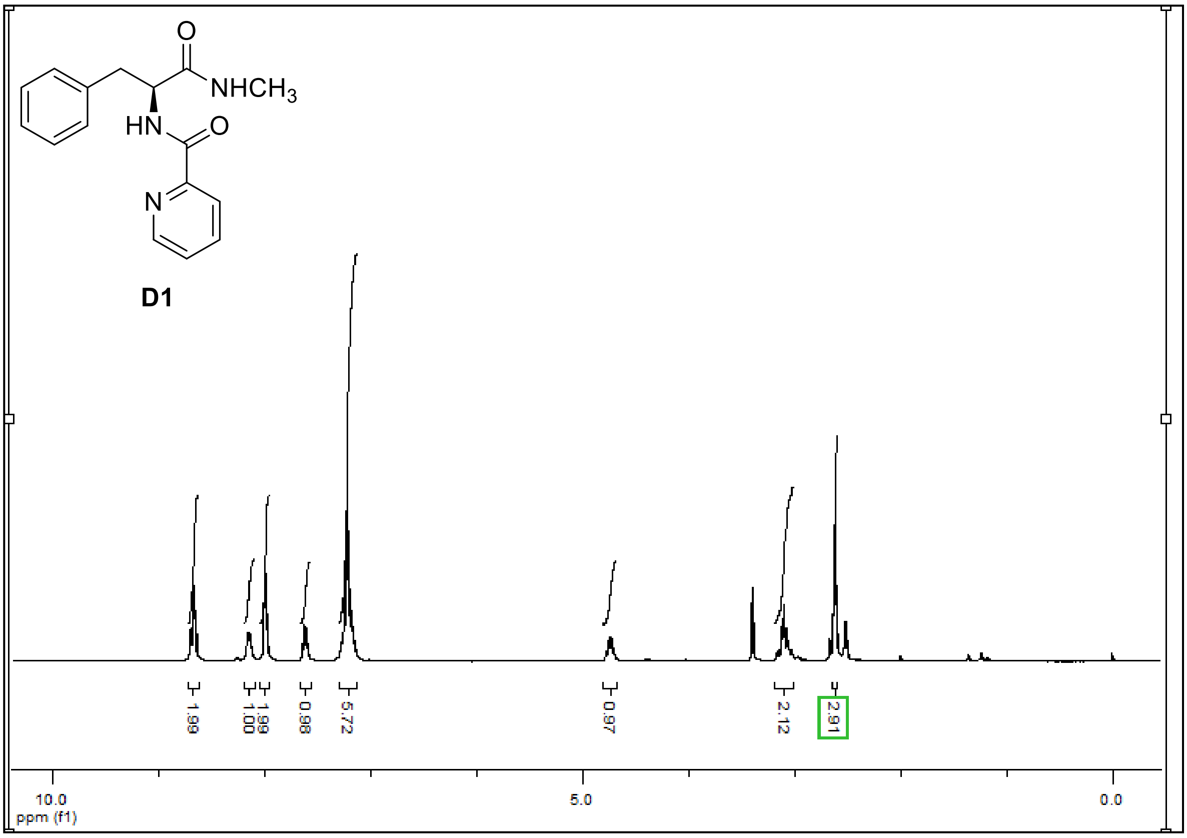

Figure S3. ^{1}H NMR (300 MHz, DMSO-d_6) spectrum of compound **D1.**

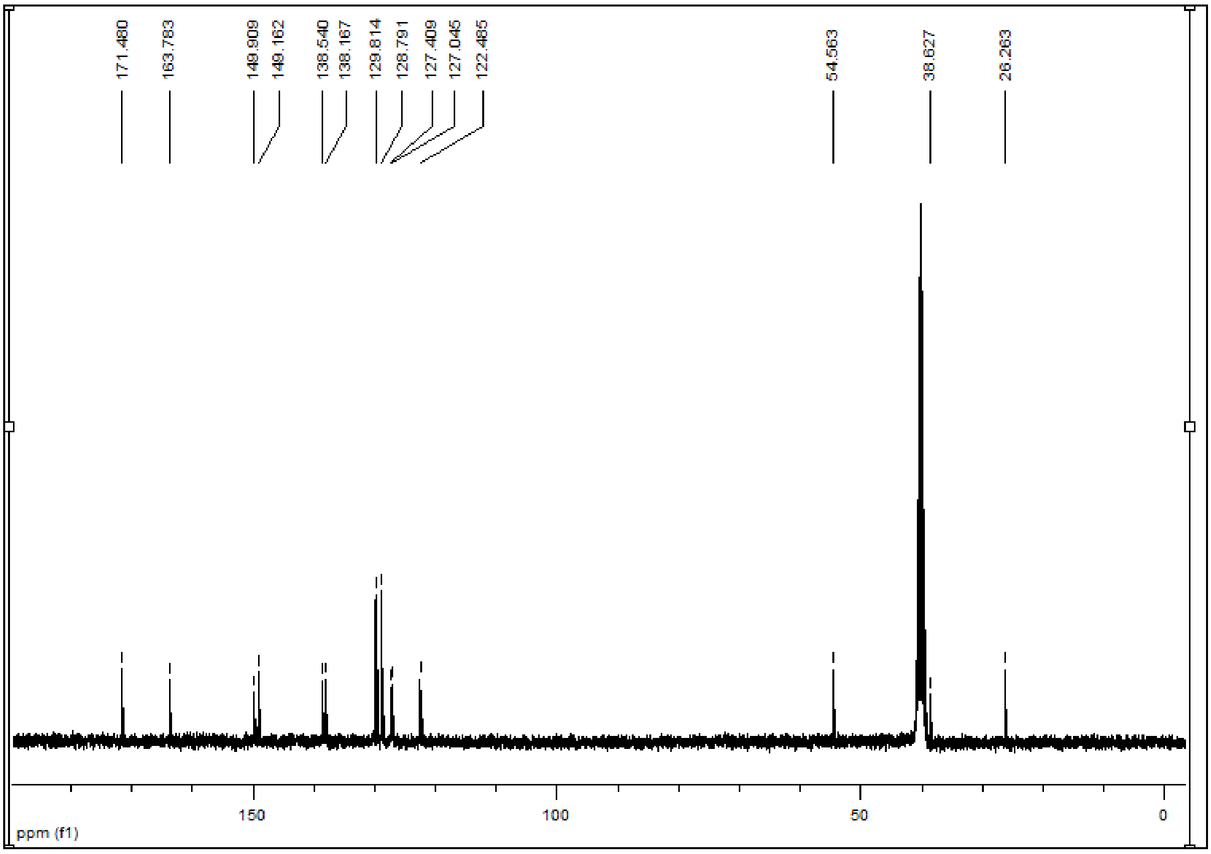

Figure S4. ^{13}C NMR (75 MHz, DMSO-d_6) spectrum of compound **D1.**

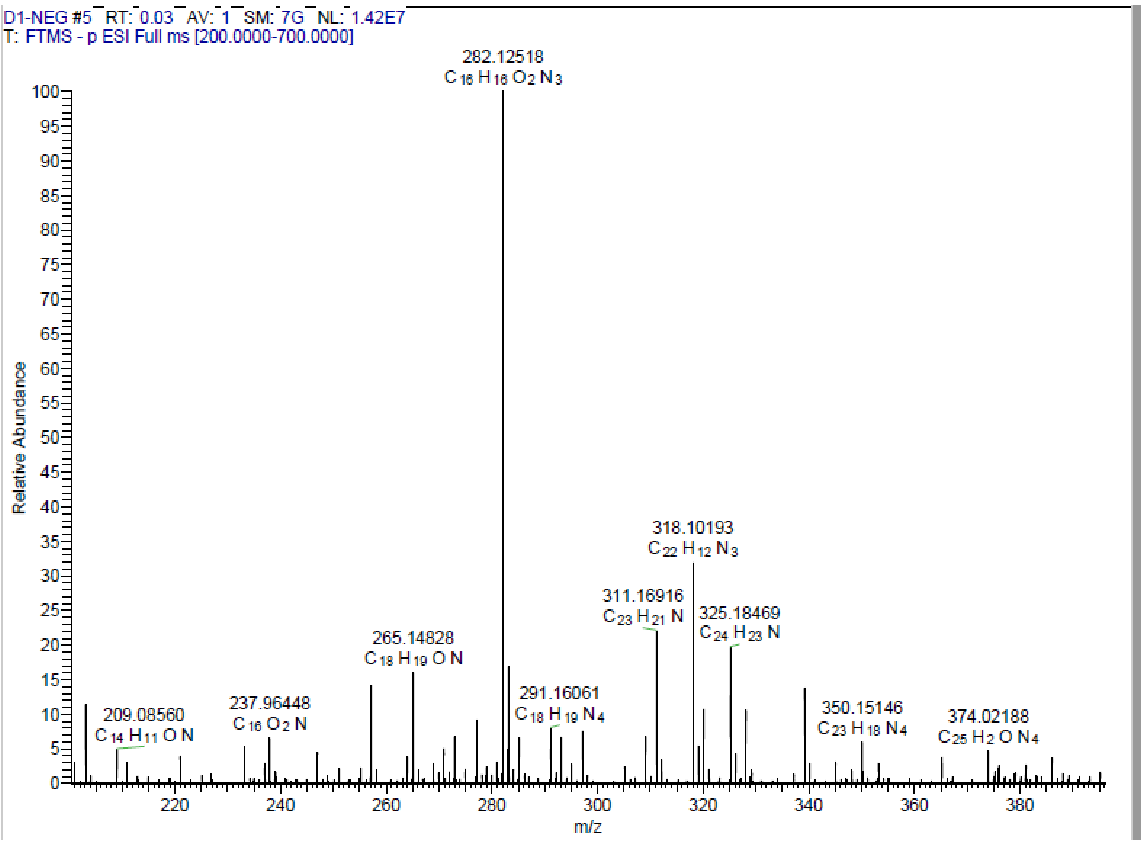

Figure S5. MS spectrum of compound **D1**

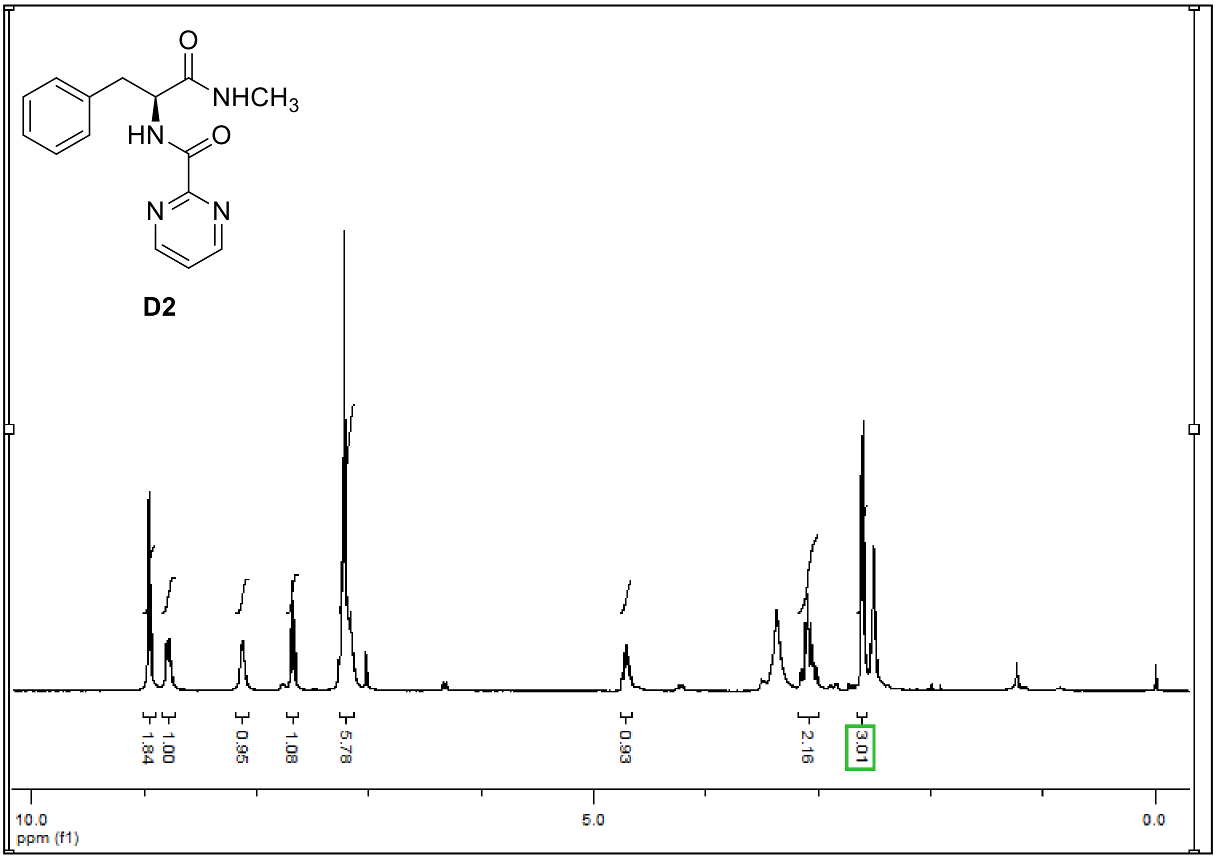

Figure S6. ^{1}H NMR (300 MHz, DMSO-d_6) spectrum of compound **D2**

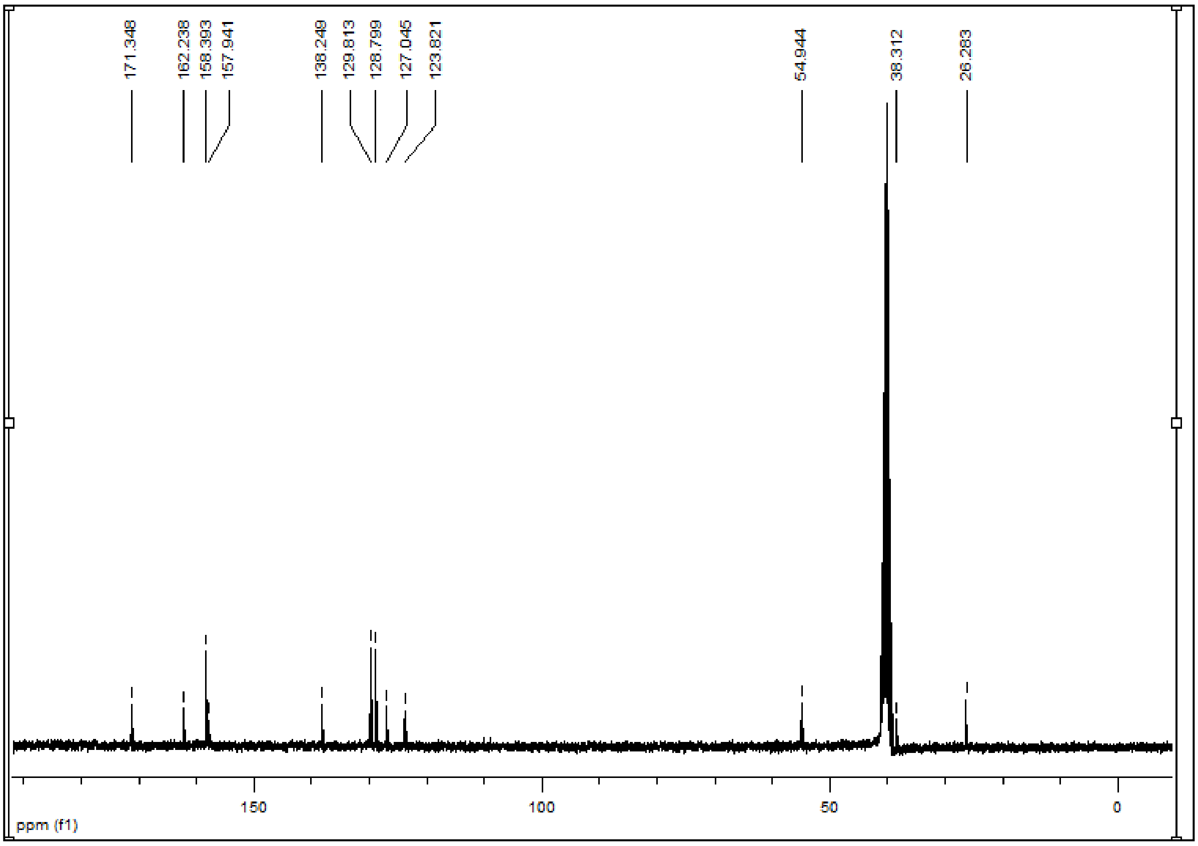

Figure S7. ^{13}C NMR (75 MHz, DMSO-d_6) spectrum of compound **D2**

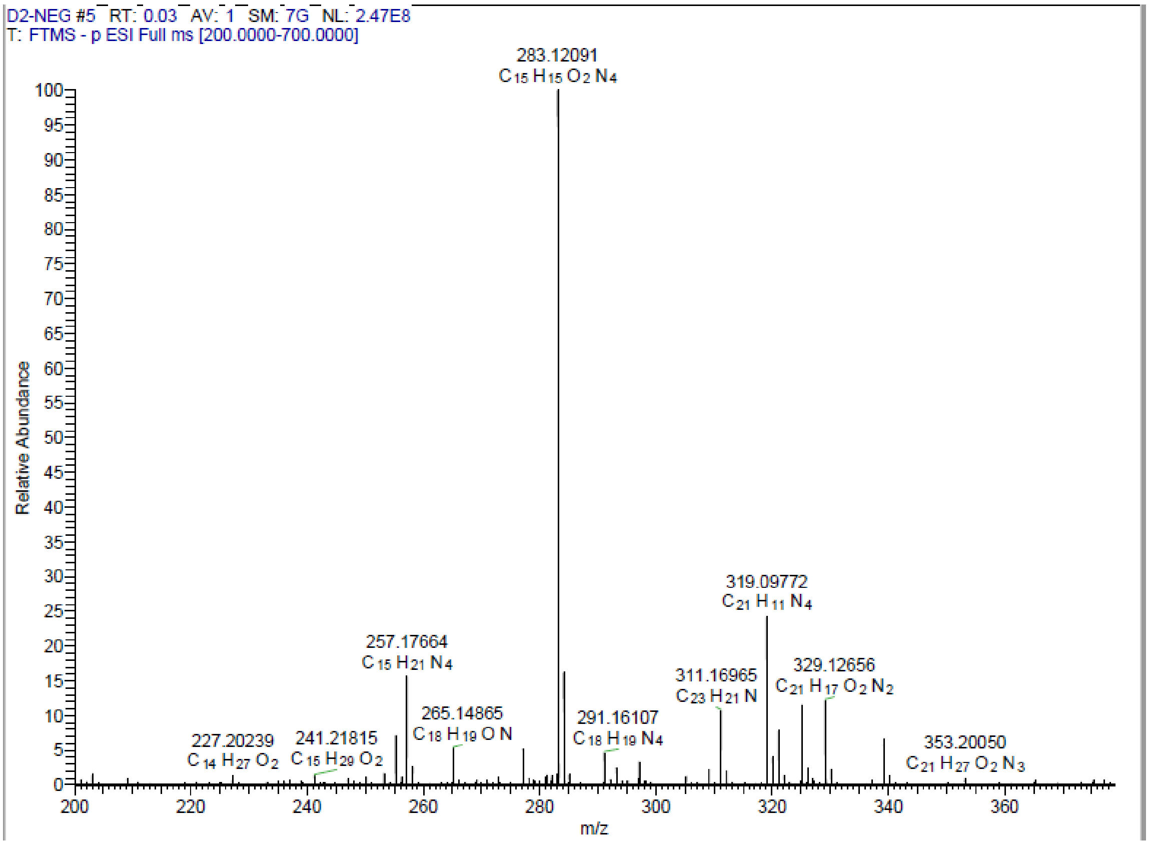

Figure S8. MS spectrum of compound **D2**

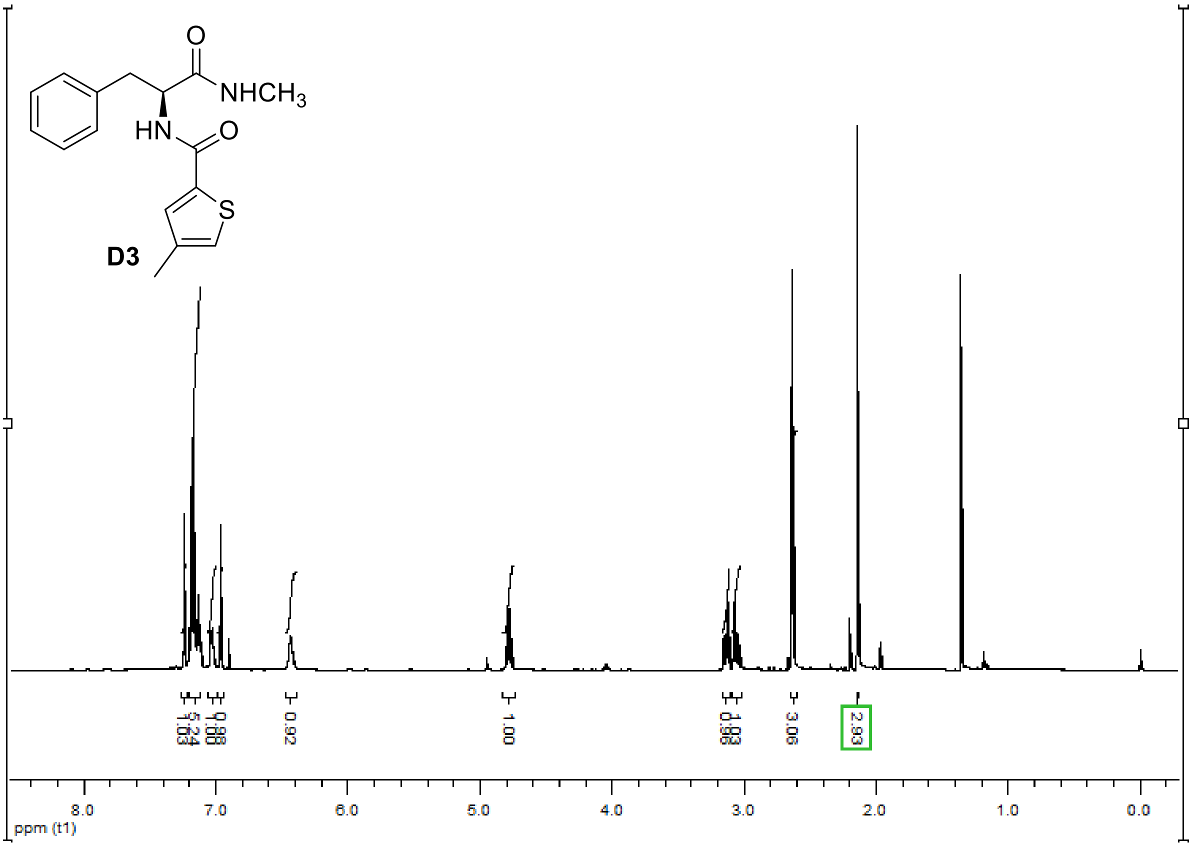

Figure S9. ^{1}H NMR (500 MHz, $CDCl_3$) spectrum of compound **D3.**

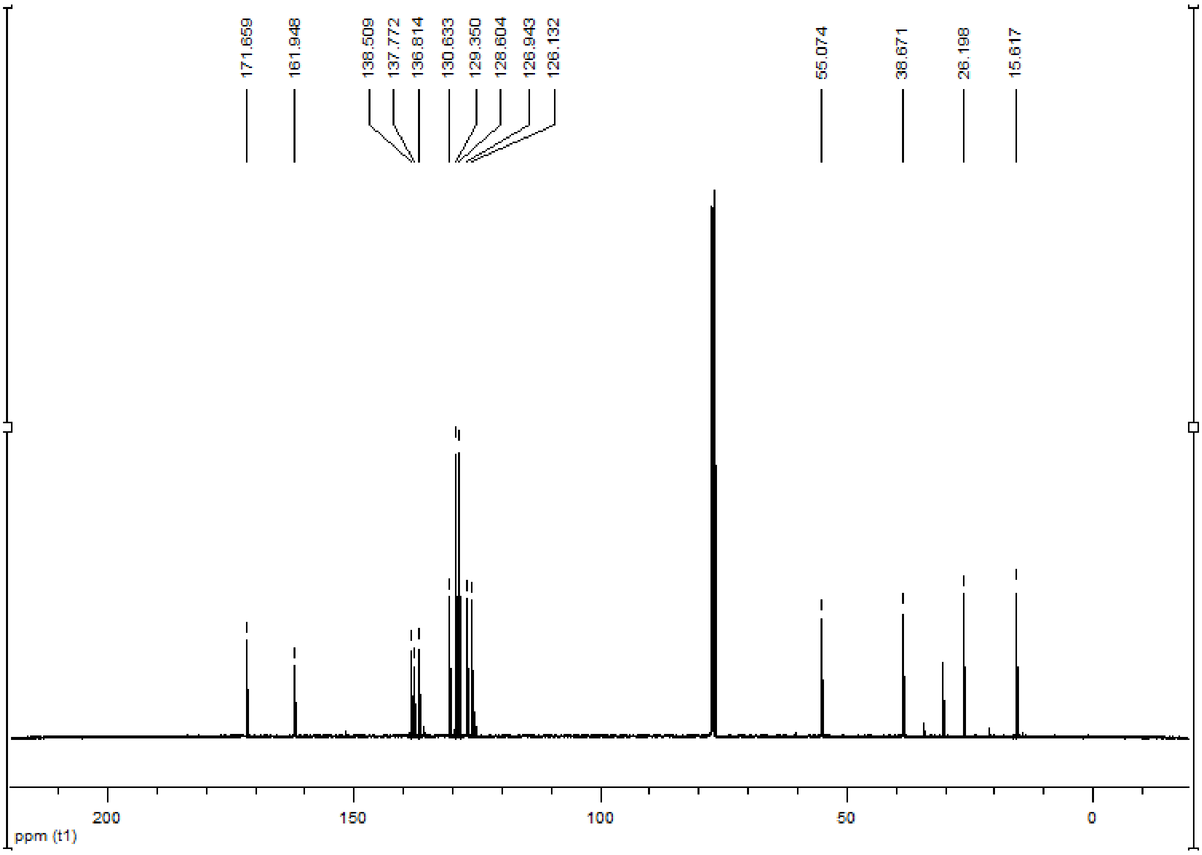

Figure S10. ^{13}C NMR (126 MHz, $CDCl_3$) spectrum of compound **D3.**

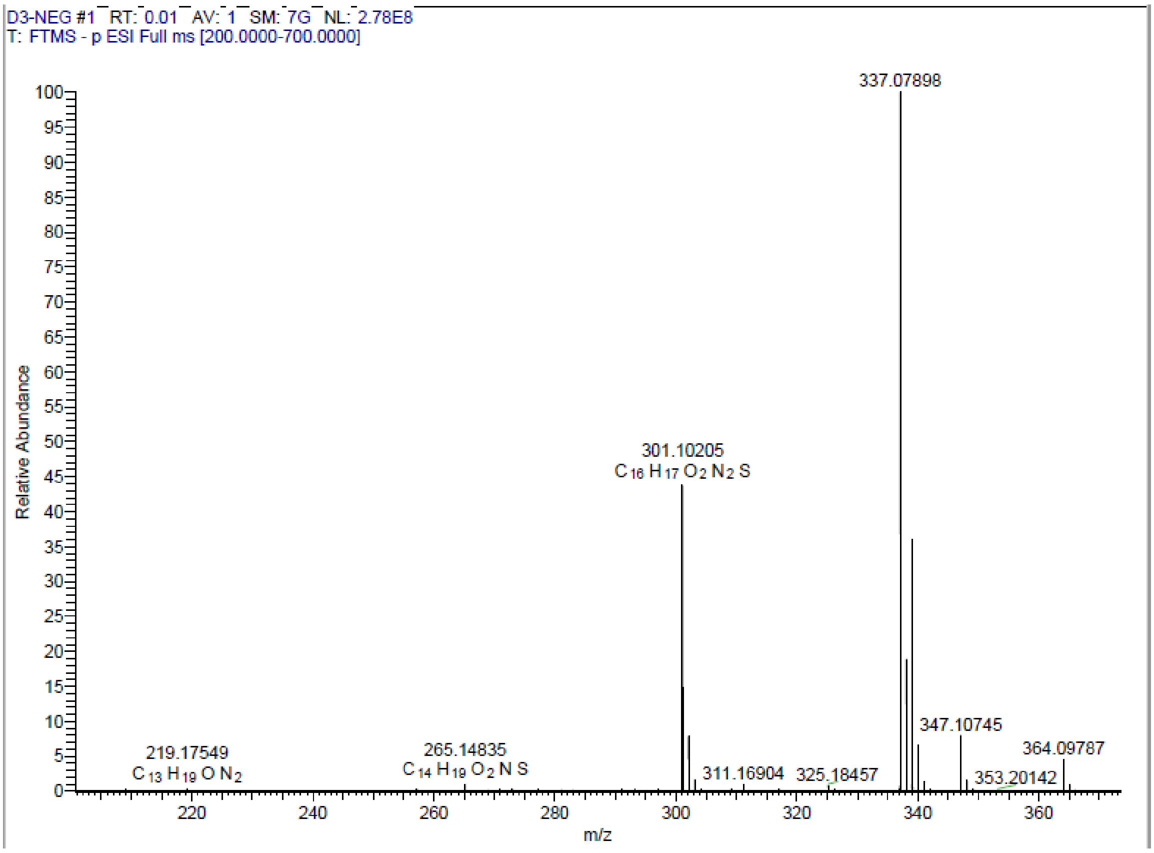

Figure S11. MS spectrum of compound **D3.**

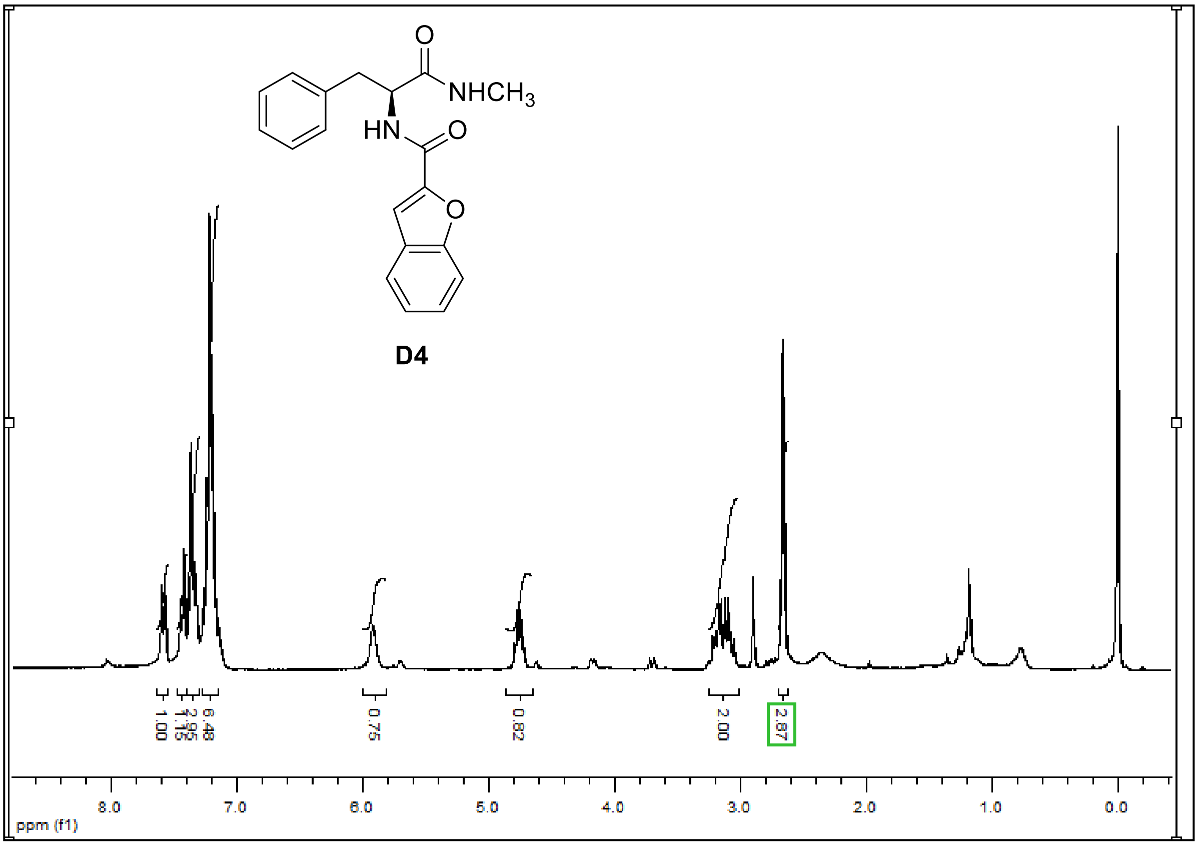

Figure S12. ^{1}H NMR (300 MHz, $CDCl_3$) spectrum of compound **D4.**

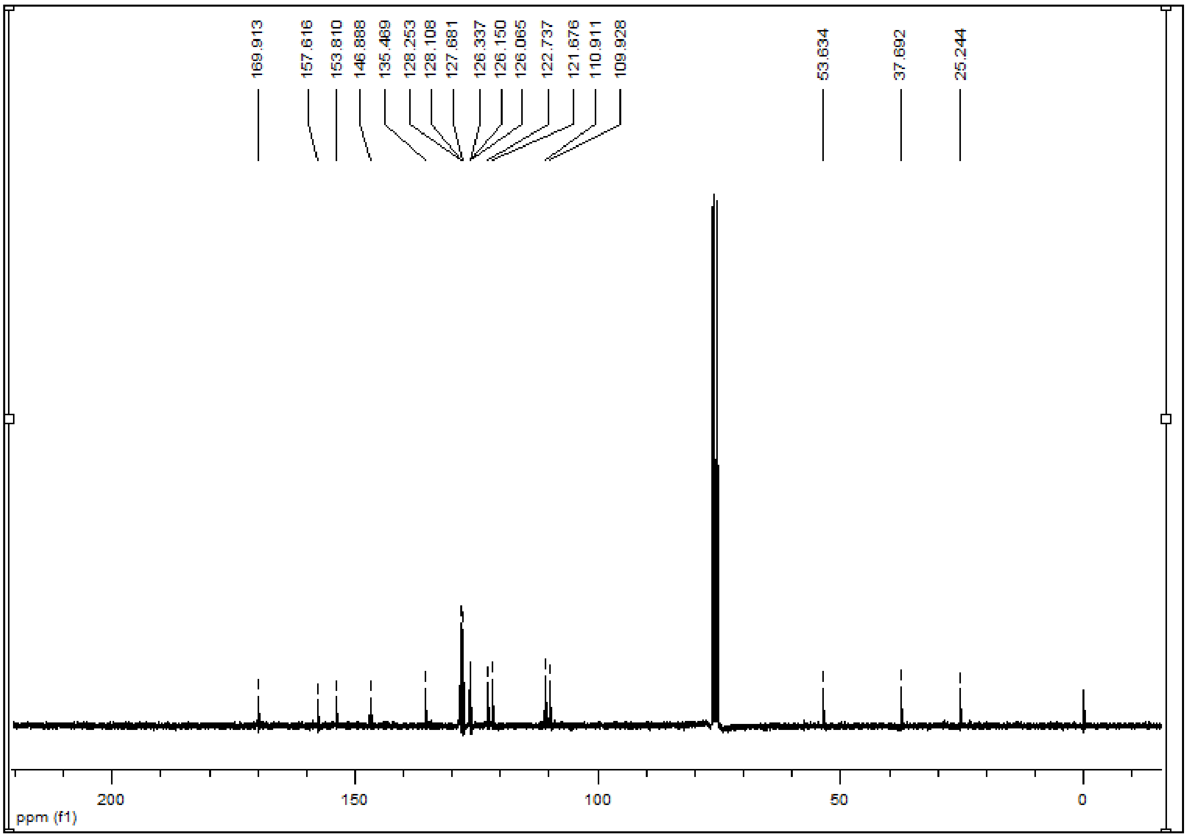

Figure S13. ^{13}C NMR (75 MHz, $CDCl_3$) spectrum of compound **D4.**

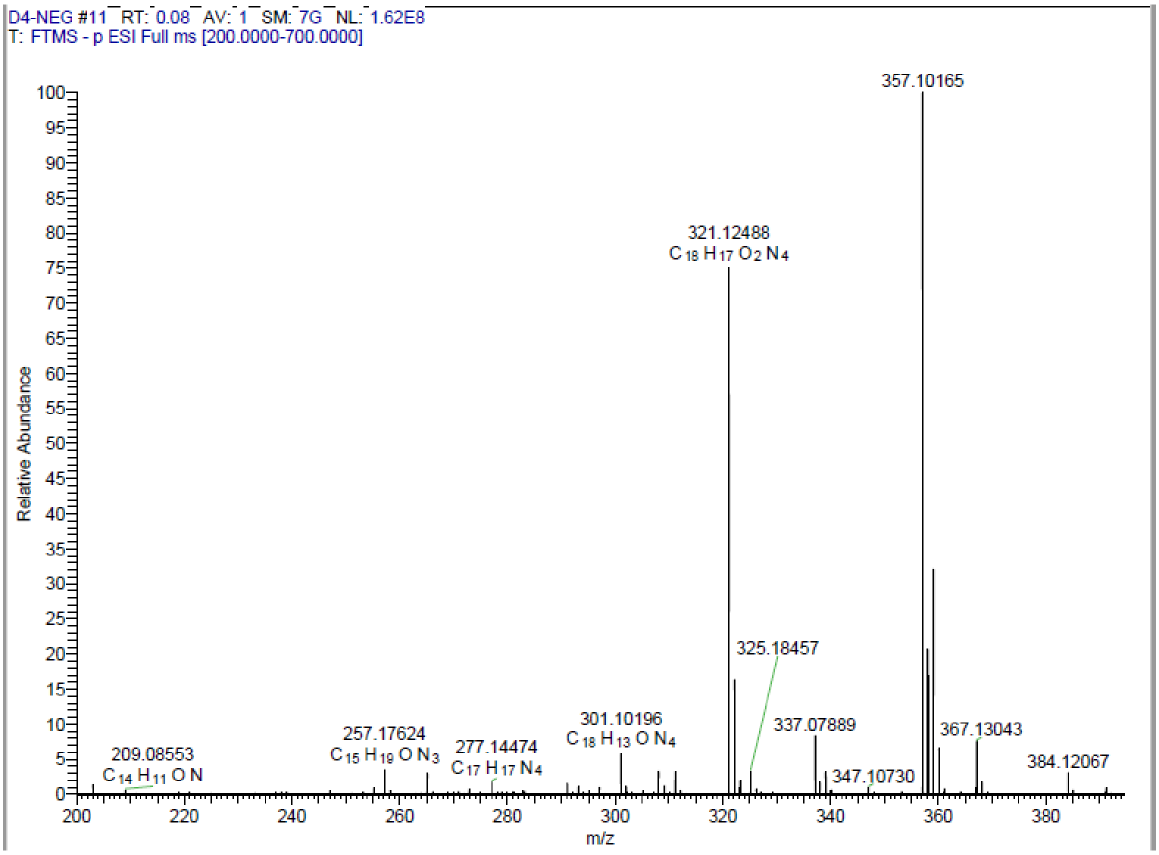

Figure S14. MS spectrum of compound **D4.**

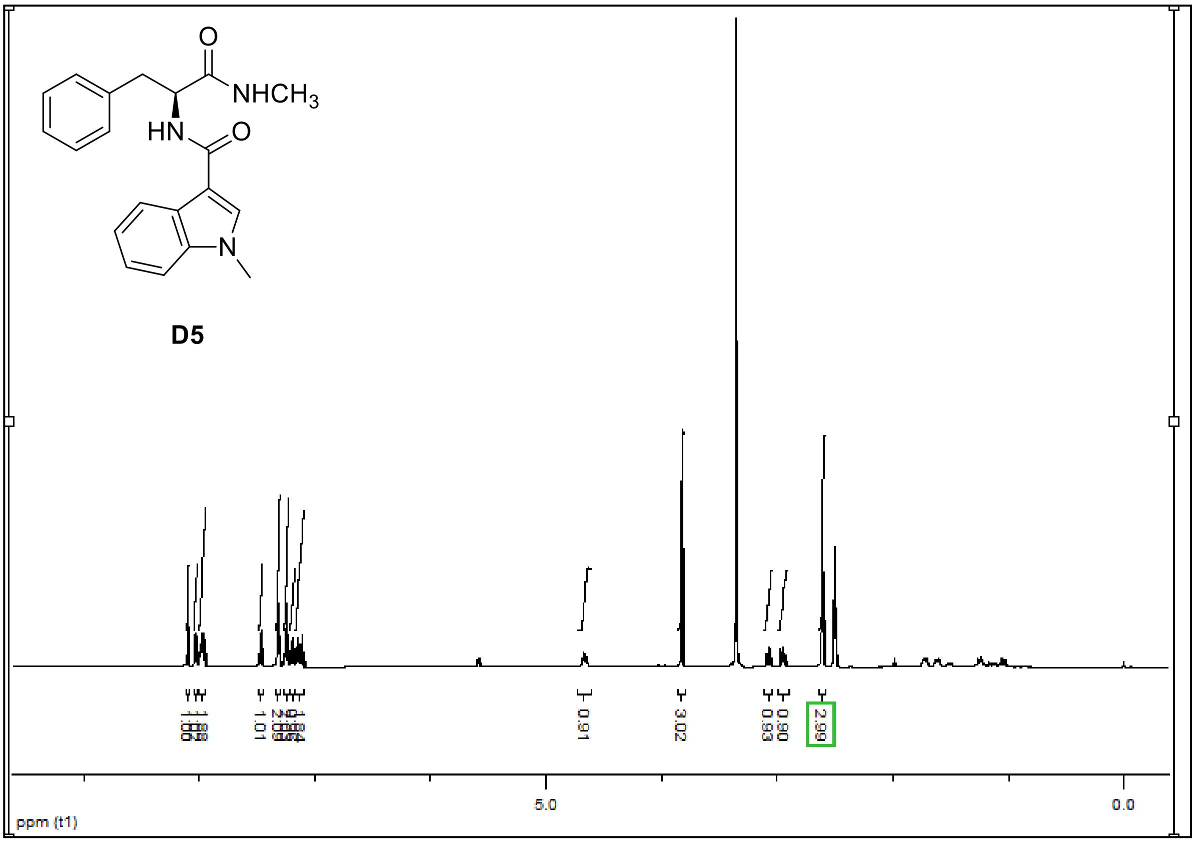

Figure S15. ^{1}H NMR (500 MHz, DMSO-d_6) spectrum of compound **D5.**

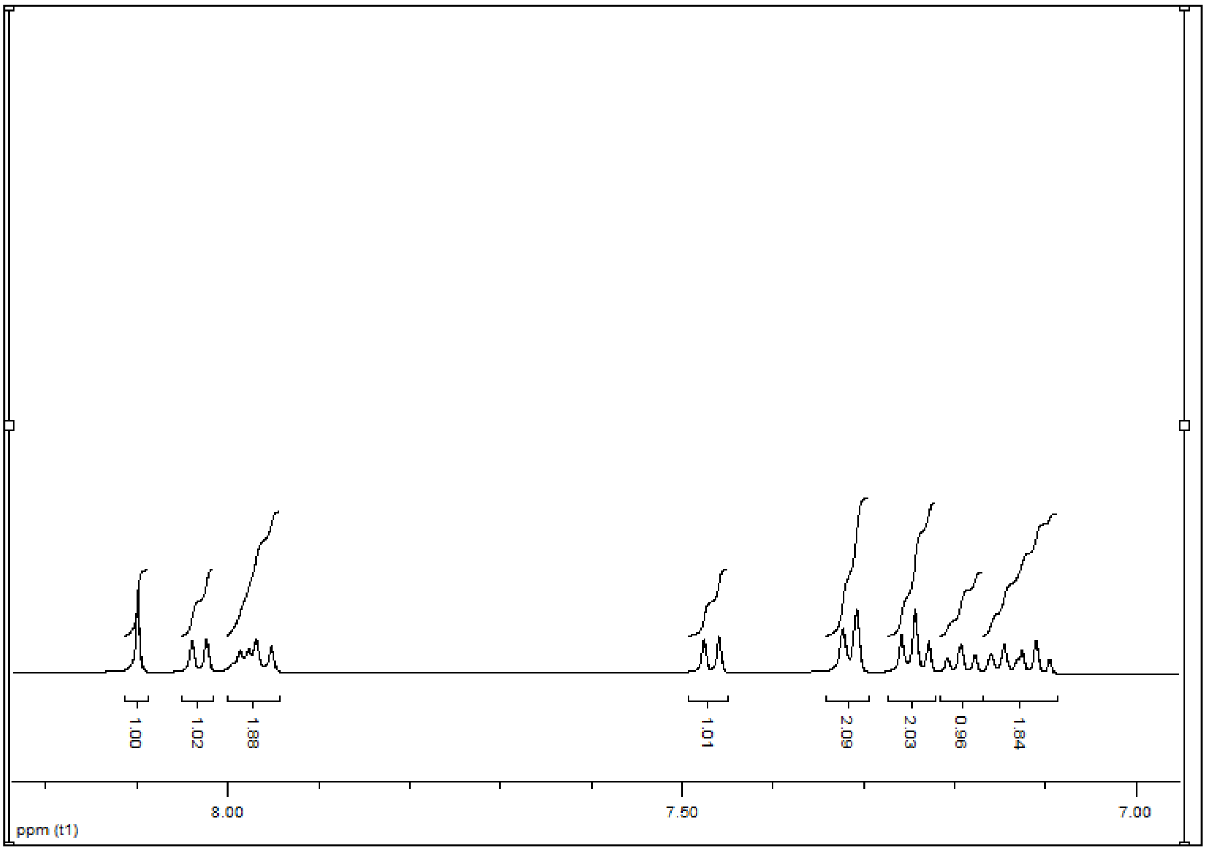

Figure S16. Extended aromatic region ^{1}H NMR (500 MHz, DMSO-d_6) spectrum of compound **D5**

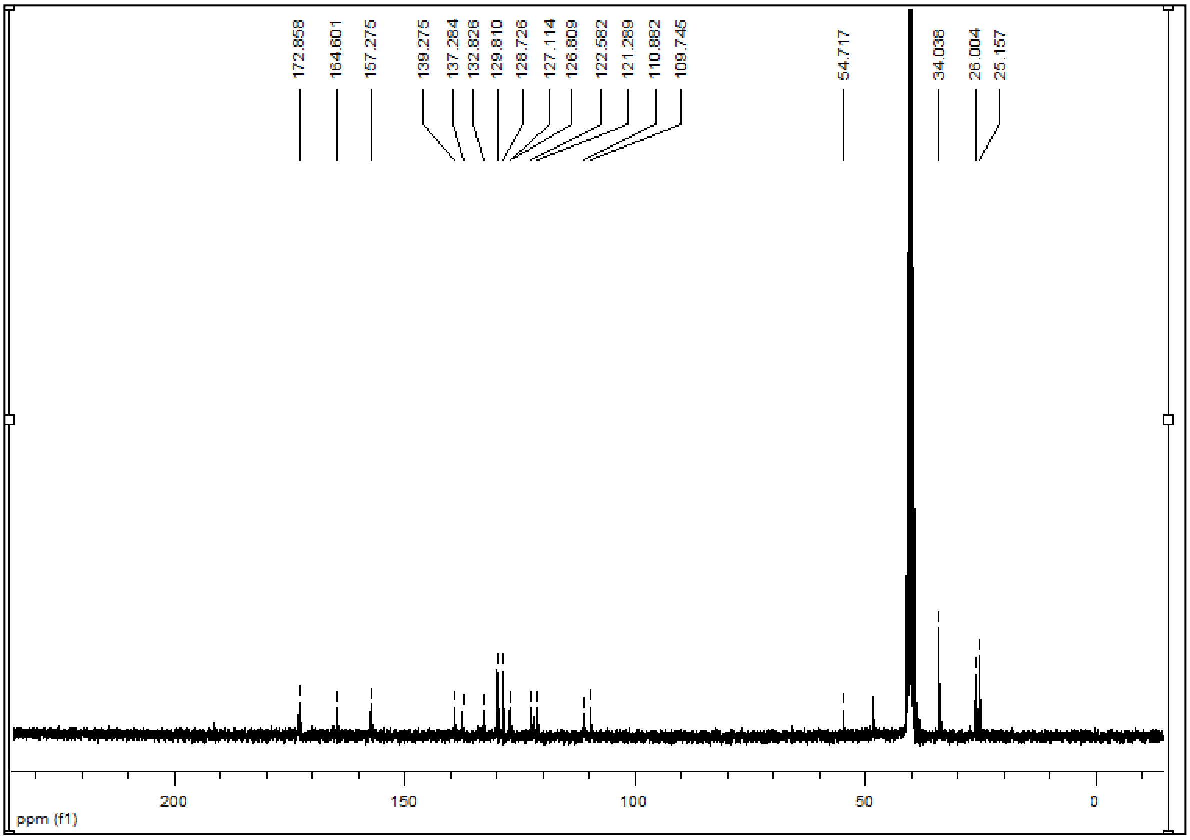

Figure S17. ^{13}C NMR (126 MHz, DMSO-d_6) spectrum of compound **D5.**

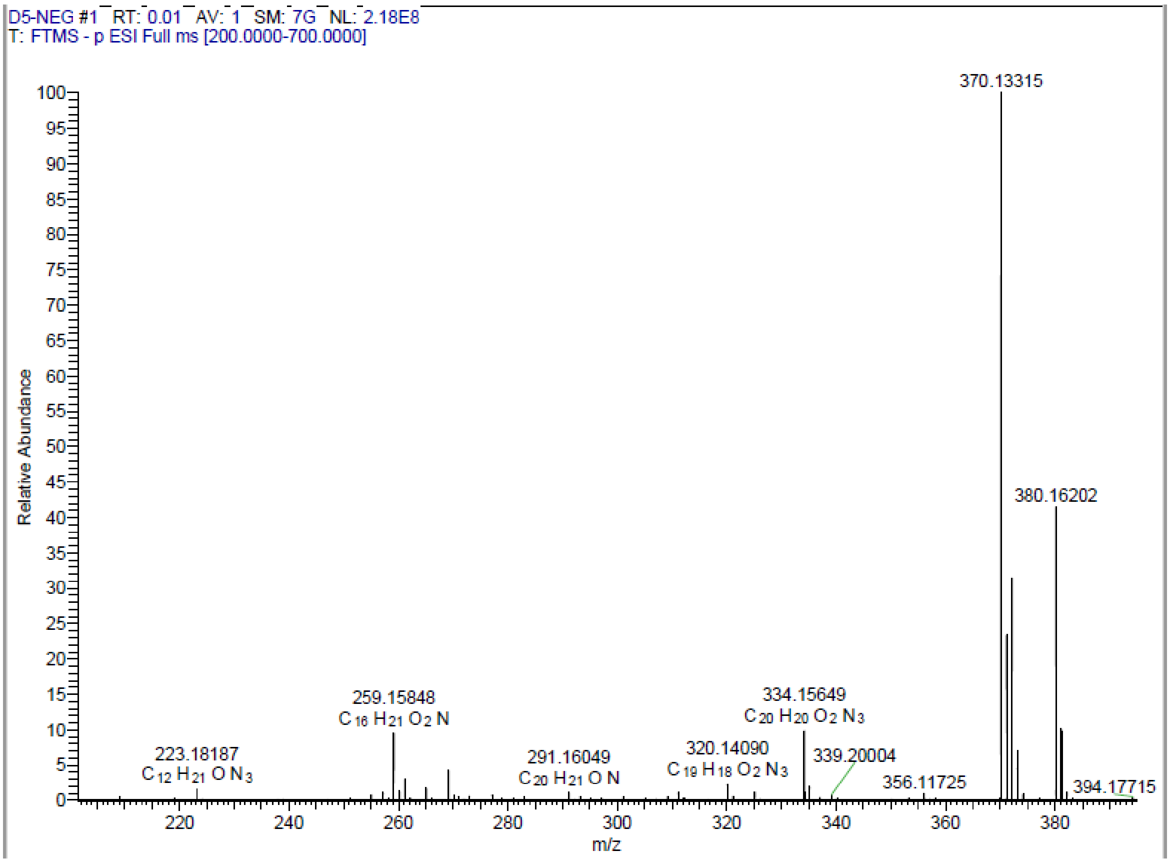

Figure S18. MS spectrum of compound **D5.**

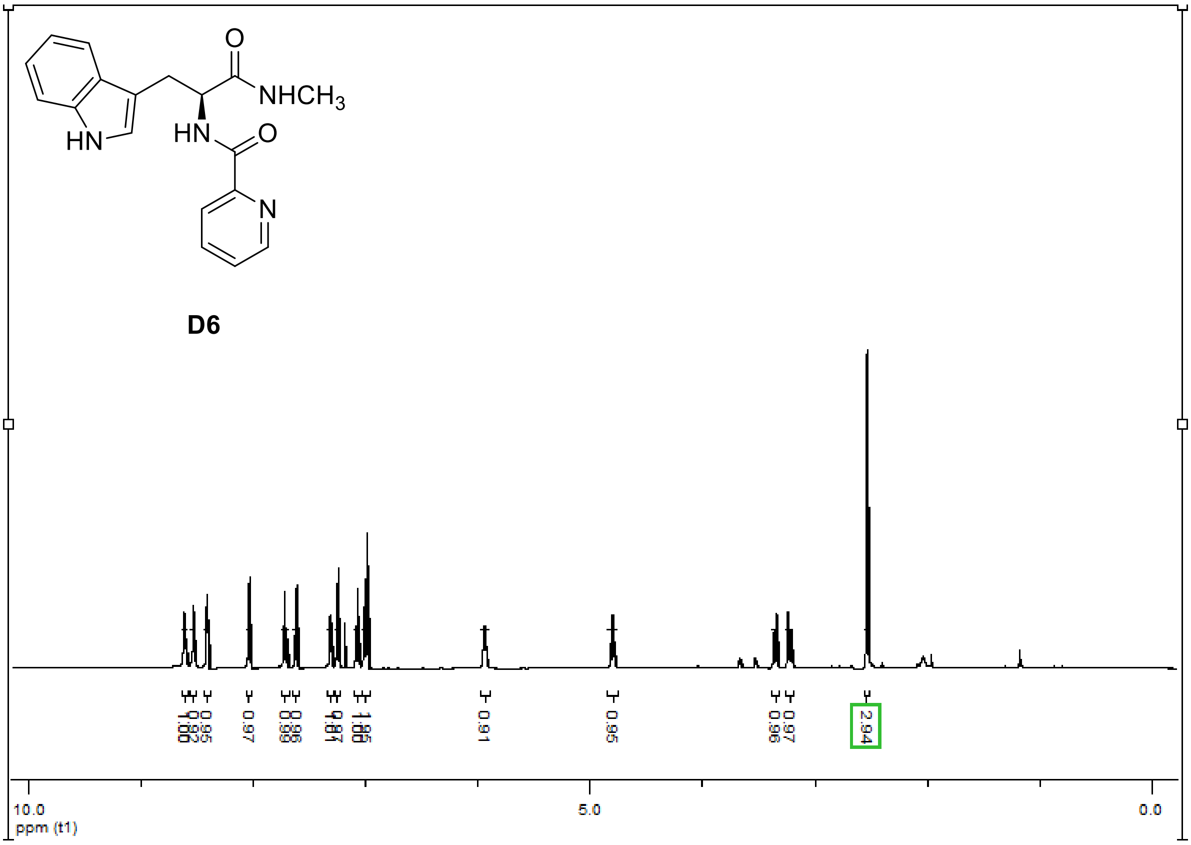

Figure S19. ^{1}H NMR (500 MHz, $CDCl_3$) spectrum of compound **D6.**

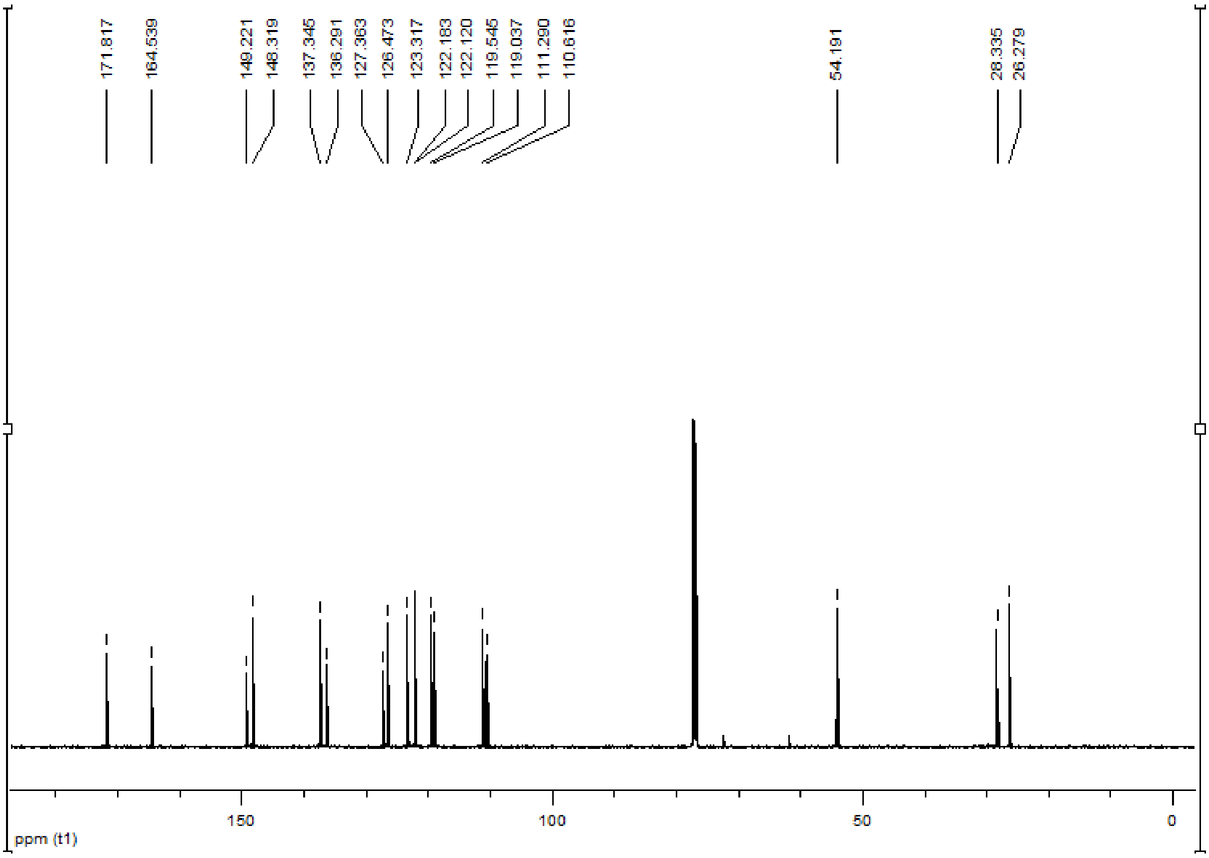

Figure S20. ^{13}C NMR (126 MHz, $CDCl_3$) spectrum of compound **D6.**

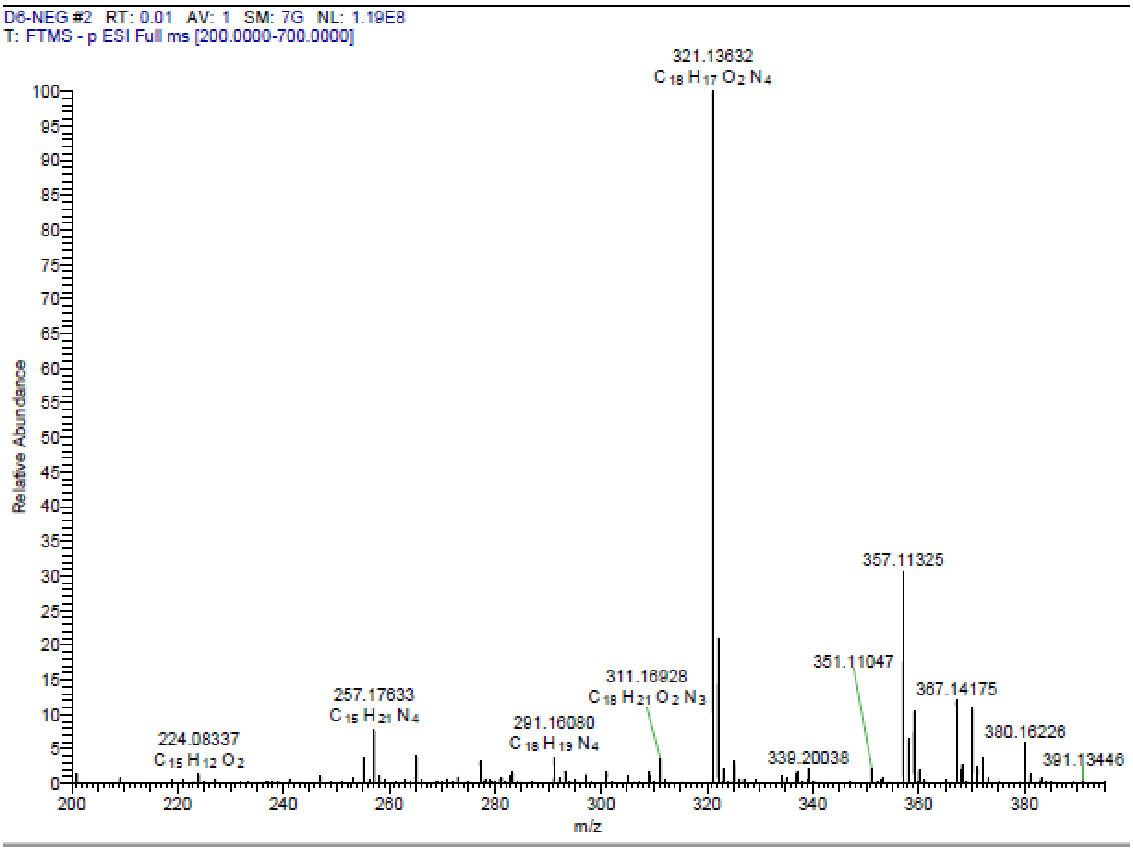

Figure S21. MS spectrum of compound **D6.**

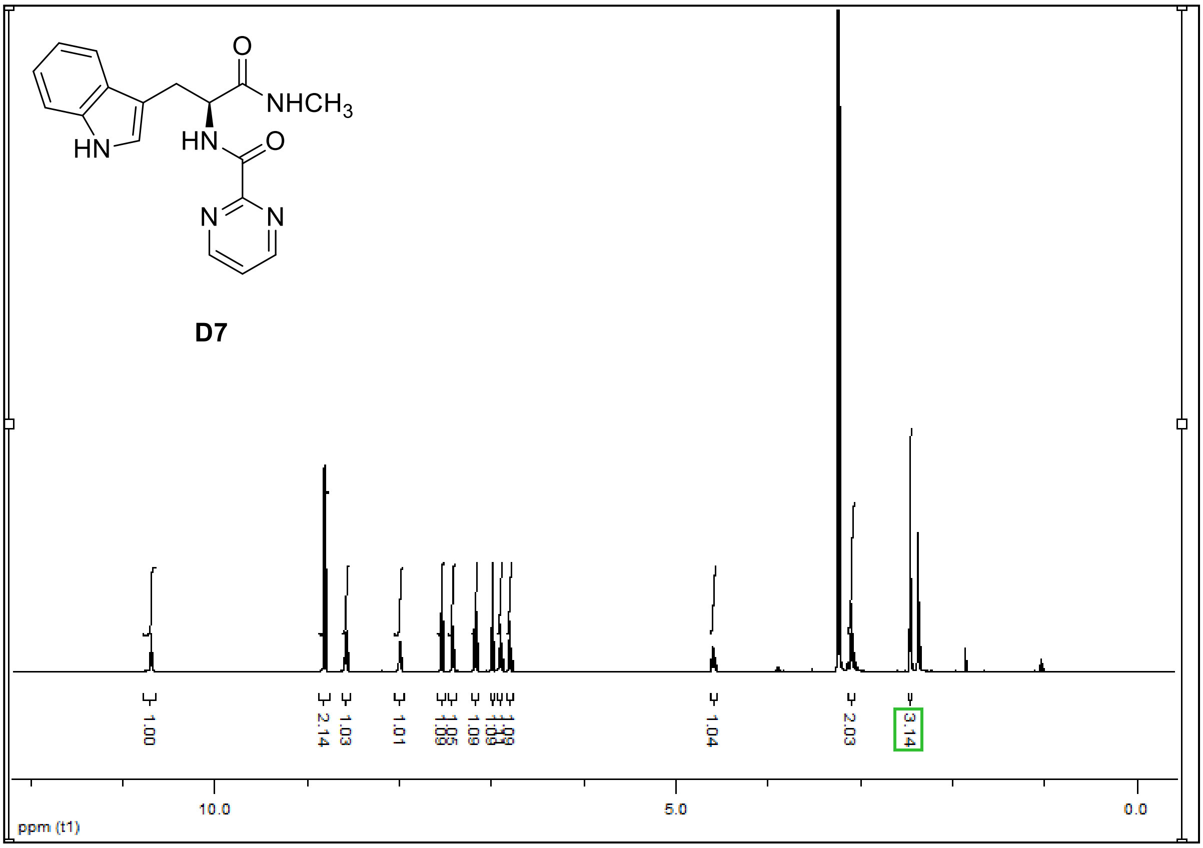

Figure S22. ^{1}H NMR (500 MHz, DMSO-d_6) spectrum of compound **D7.**

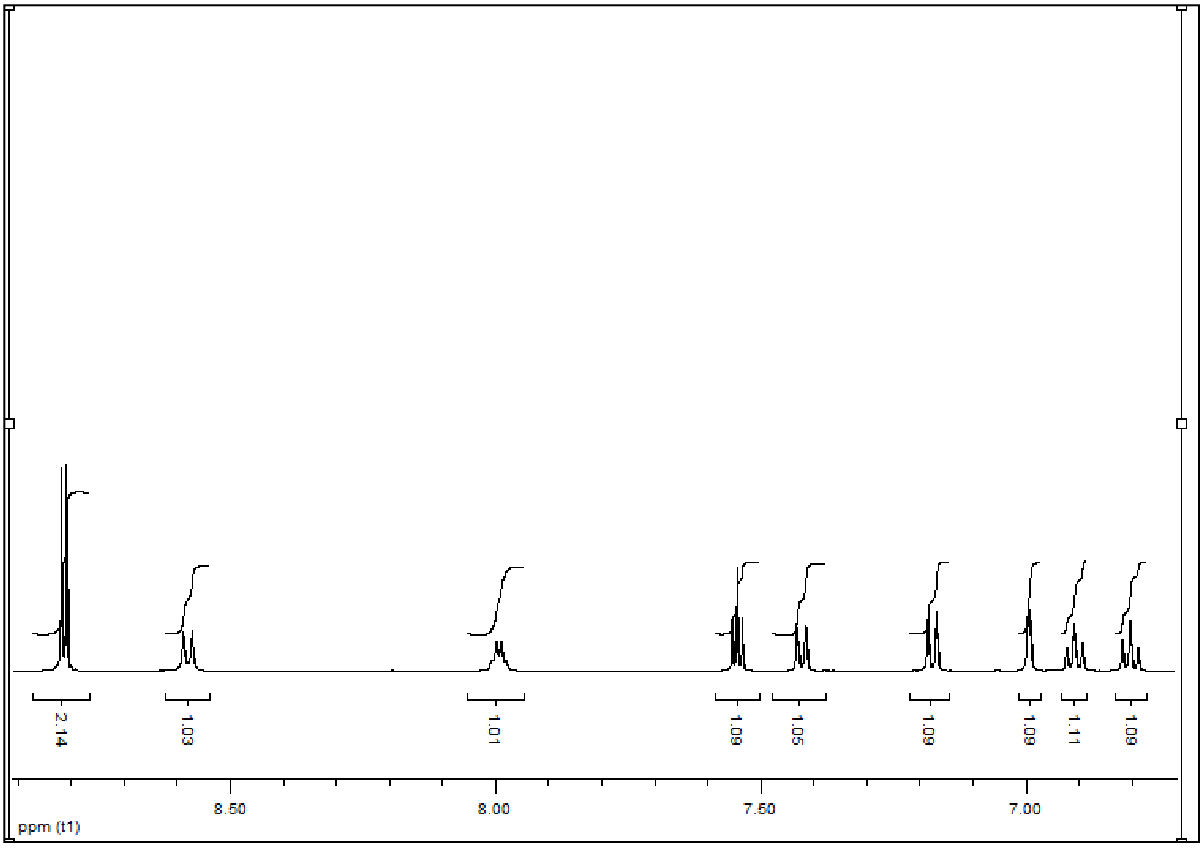

Figure S23. Extended aromatic region ^{1}H NMR (500 MHz, DMSO-d_6) spectrum of compound **D7.**

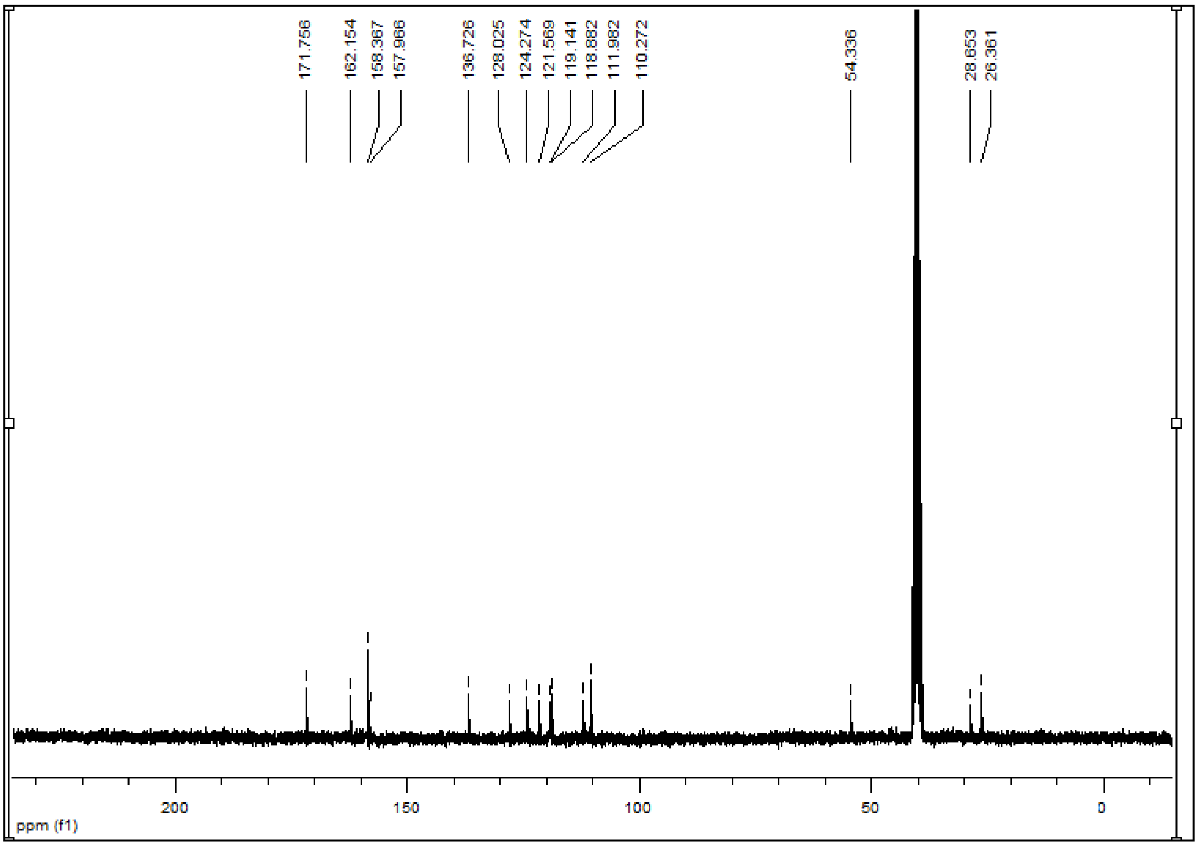

Figure S24. ^{13}C NMR (126 MHz, DMSO-d_6) spectrum of compound **D7.**

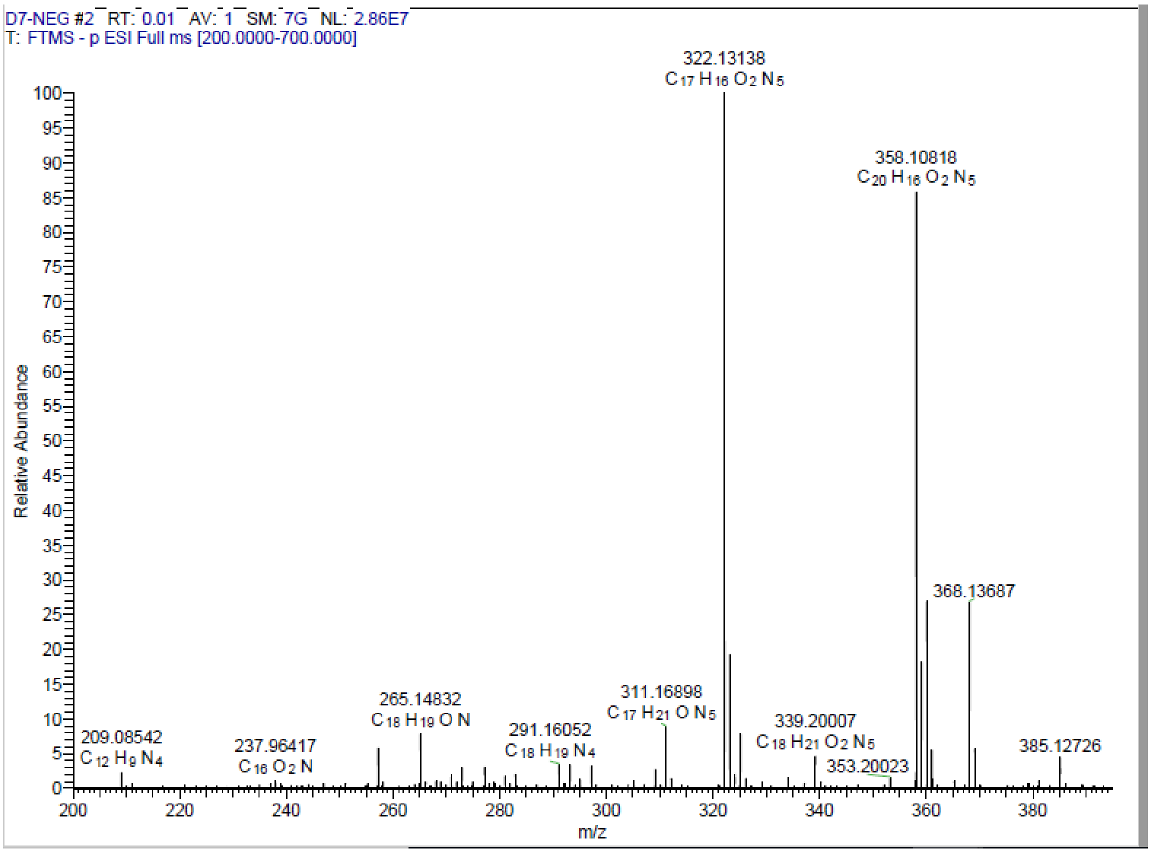

Figure S25. MS spectrum of compound **D7.**

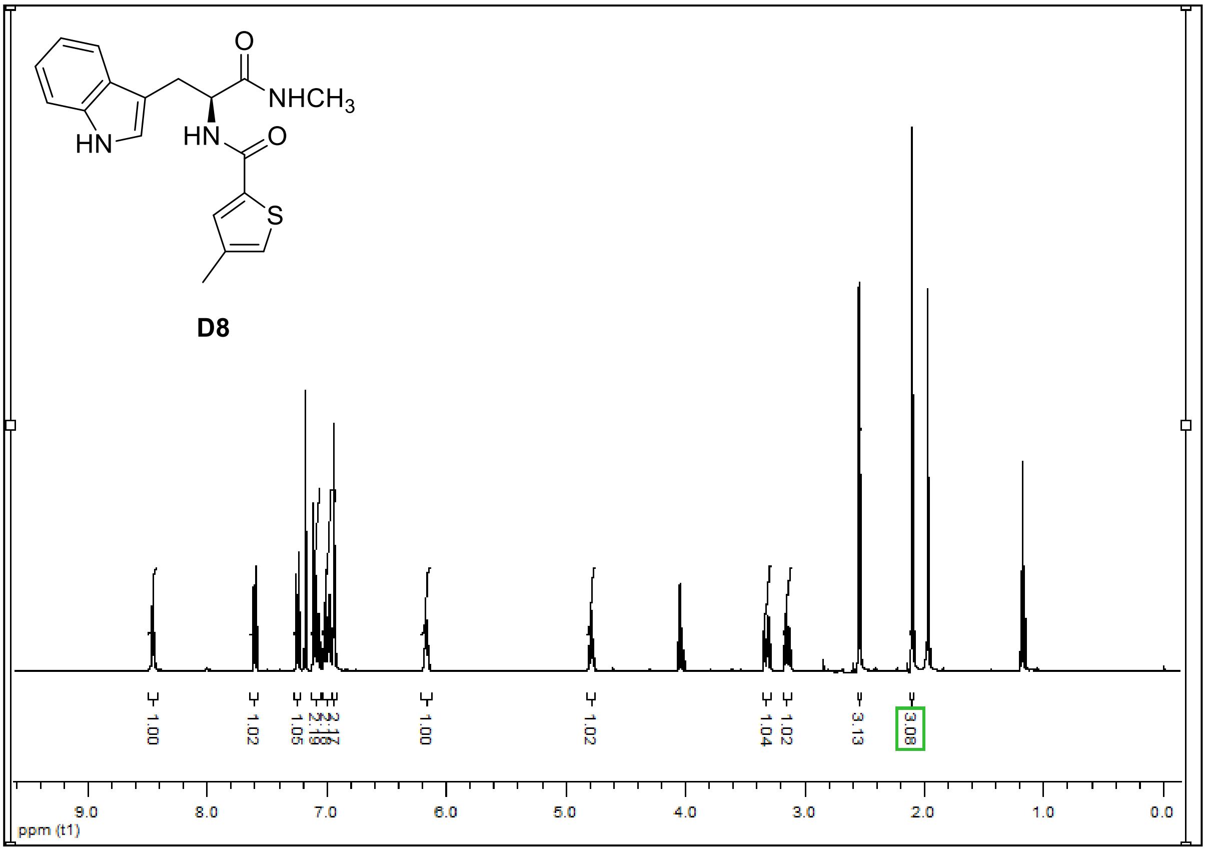

Figure S26. ^{1}H NMR (500 MHz, $CDCl_3$) spectrum of compound **D8.**

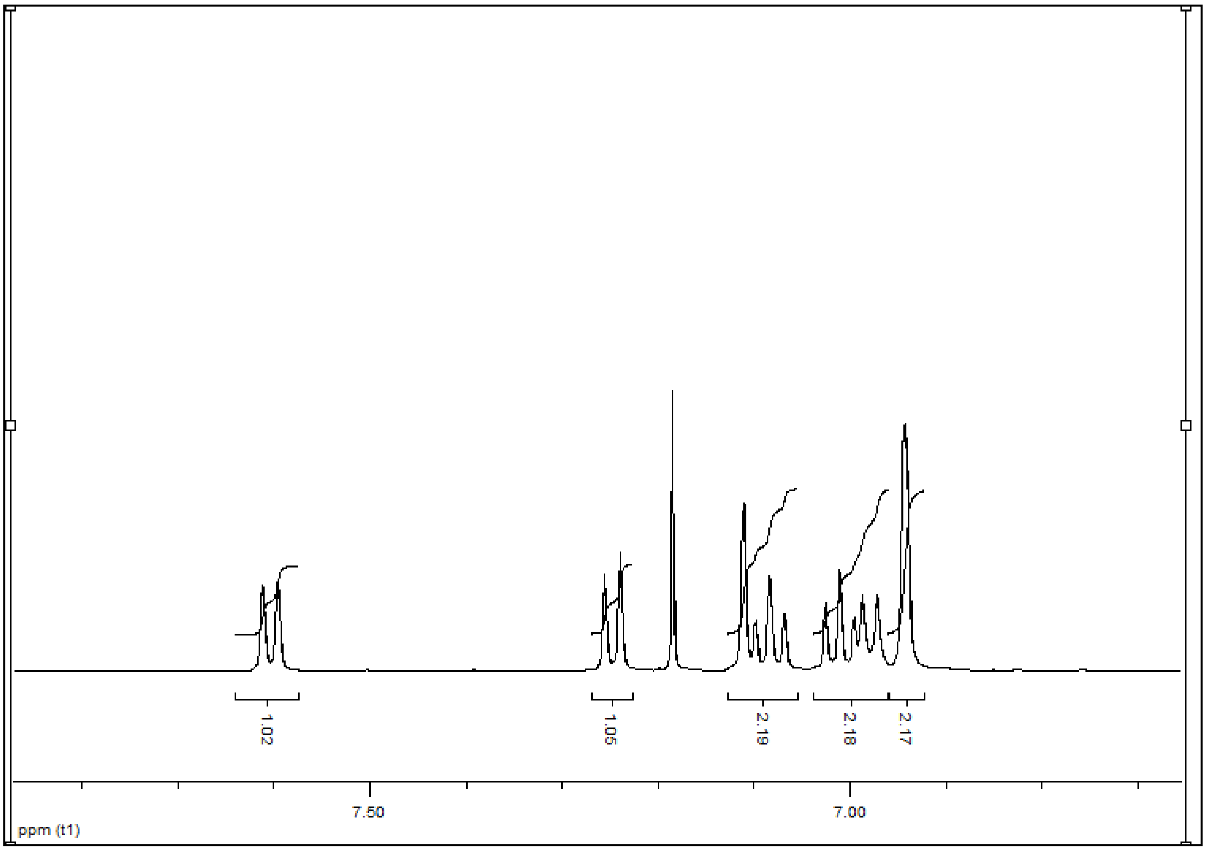

Figure S27. Extended aromatic region ^{1}H NMR (500 MHz, $CDCl_3$) spectrum of compound **D8.**

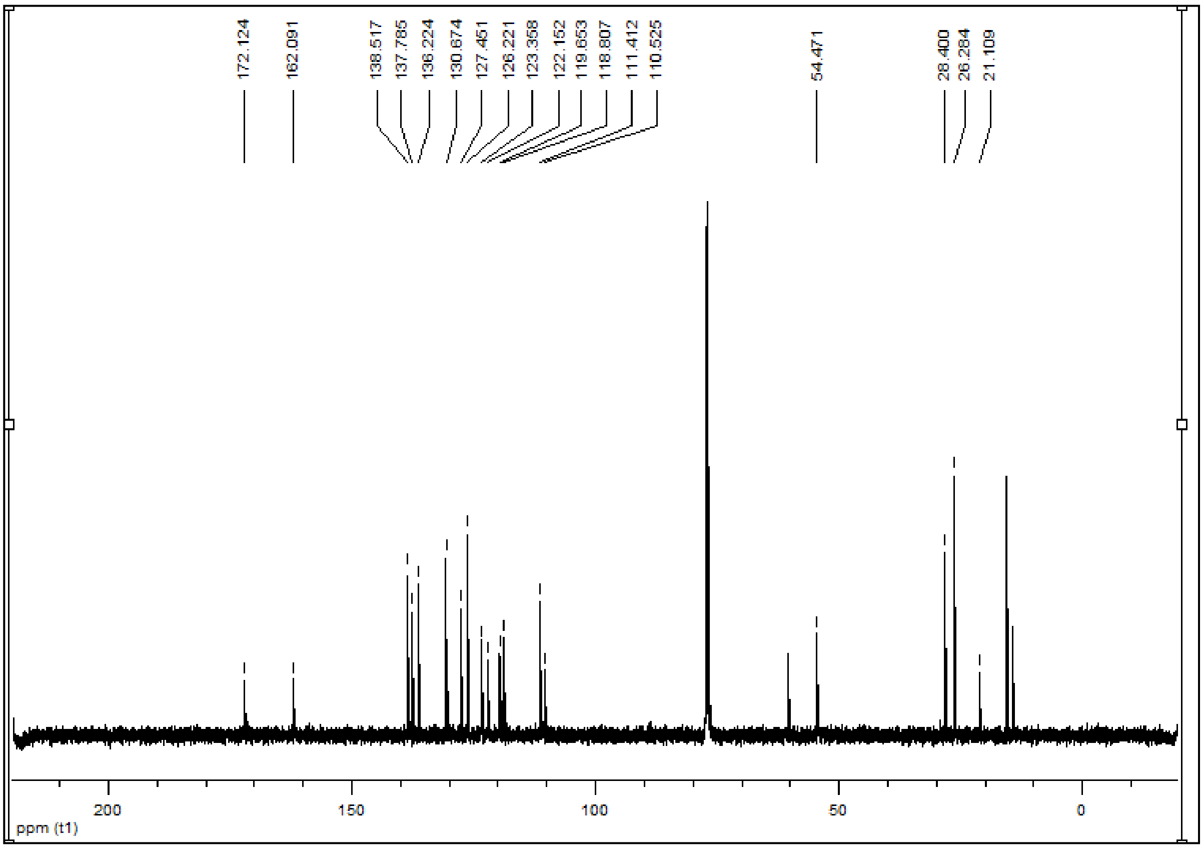

Figure S28. ^{13}HC NMR (126 MHz, $CDCl_3$) spectrum of compound **D8.**

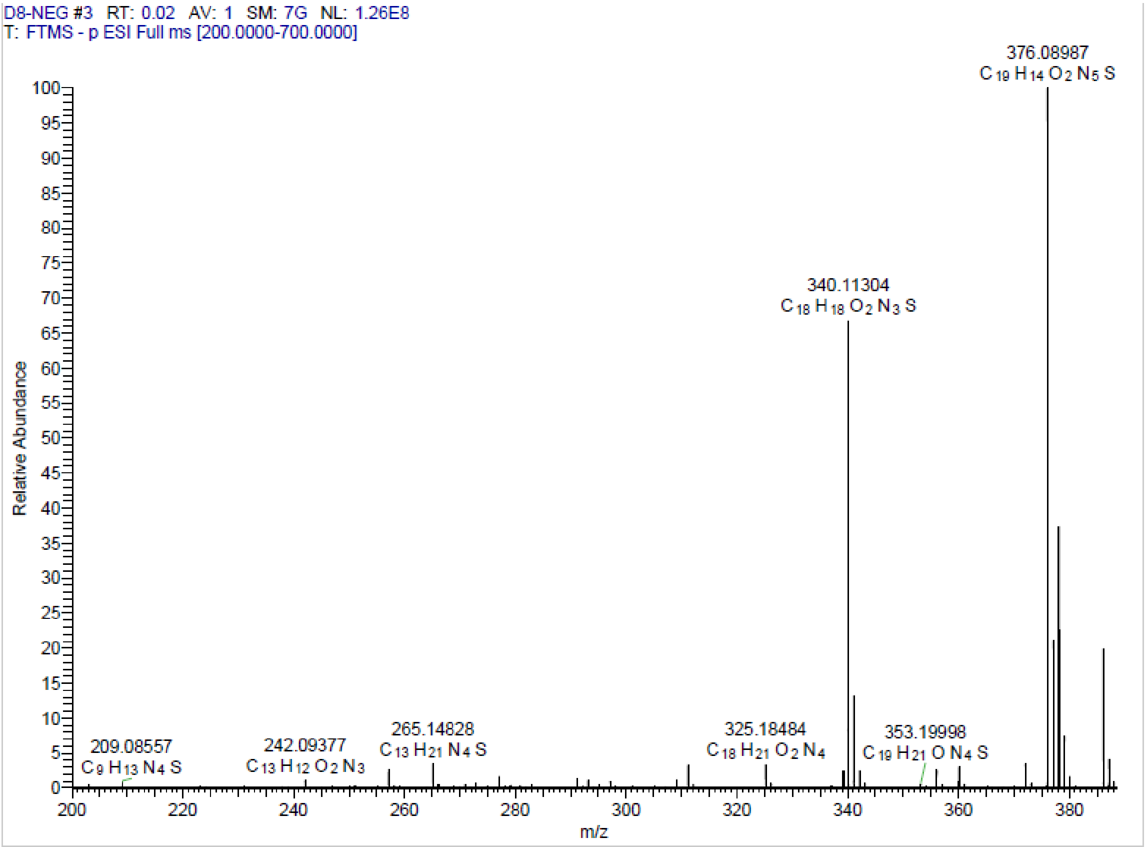

Figure S29. MS spectrum of compound **D8.**

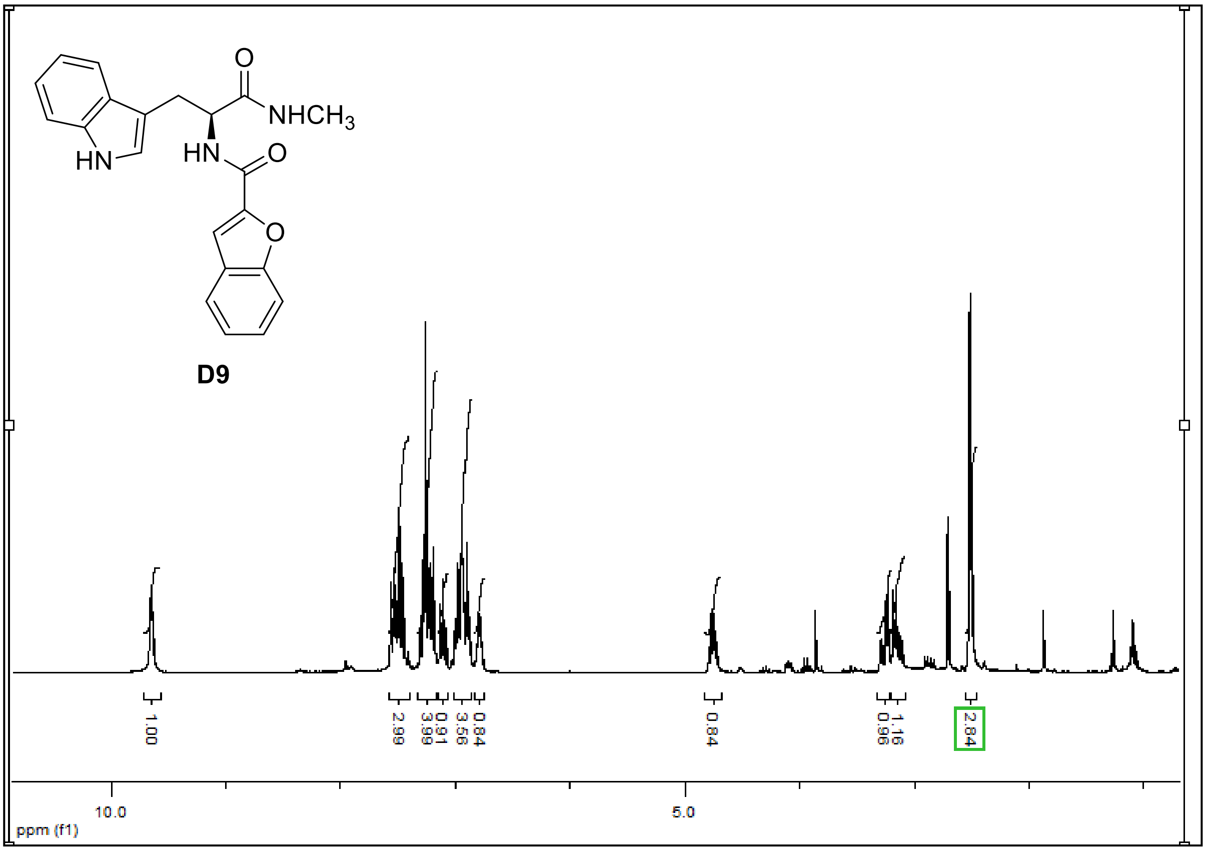

Figure S30. ^{1}H NMR (300 MHz, $CDCl_3$) spectrum of compound **D9.**

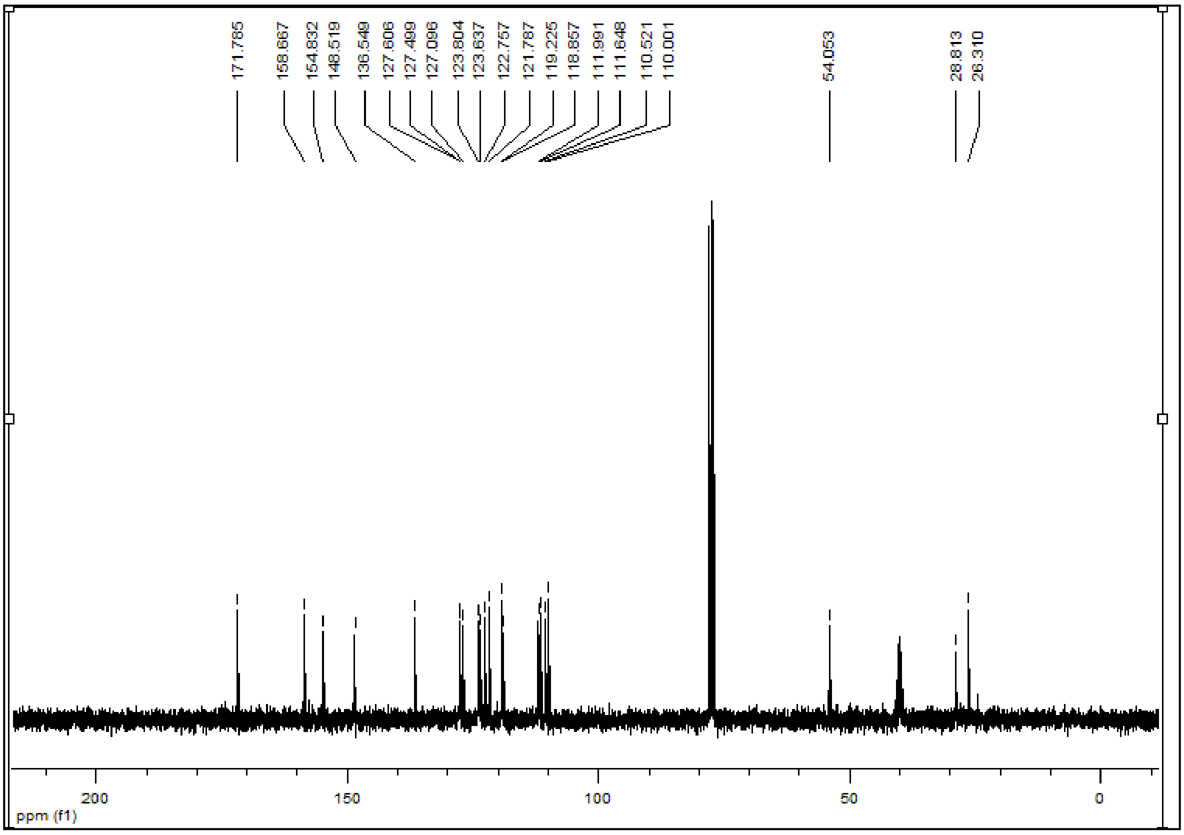

Figure S31. ^{13}C NMR (75 MHz, $CDCl_3$) spectrum of compound **D9.**

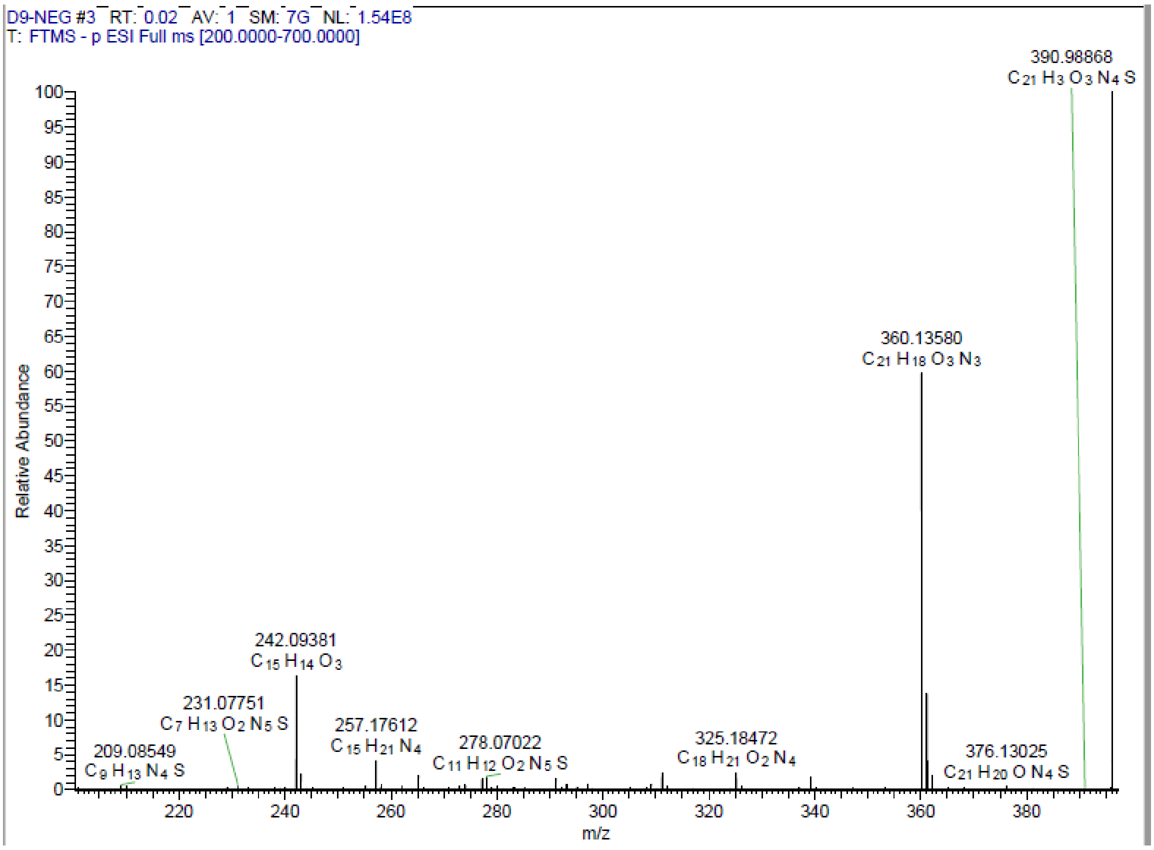

Figure S32. MS spectrum of compound **D9.**

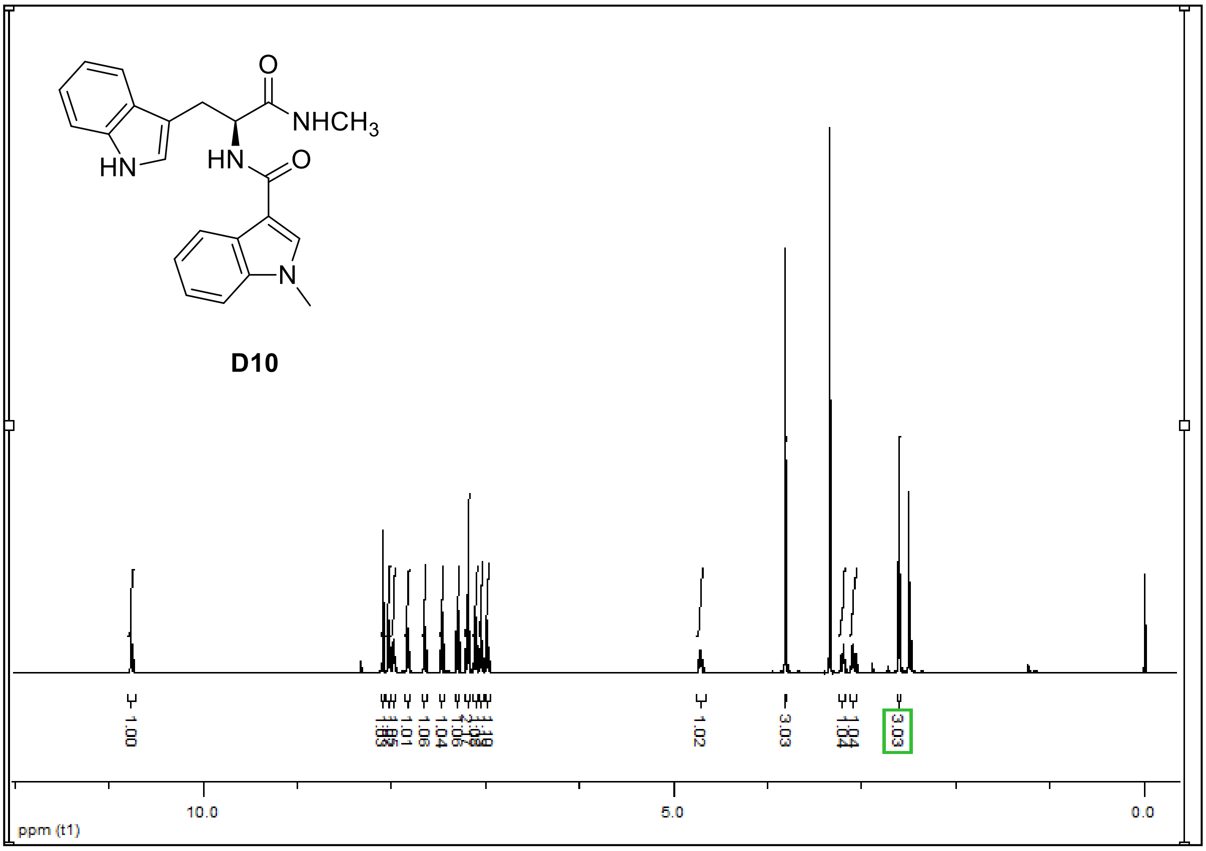

Figure S33. ^{1}H NMR (500 MHz, DMSO-d_6) spectrum of compound **D10.**

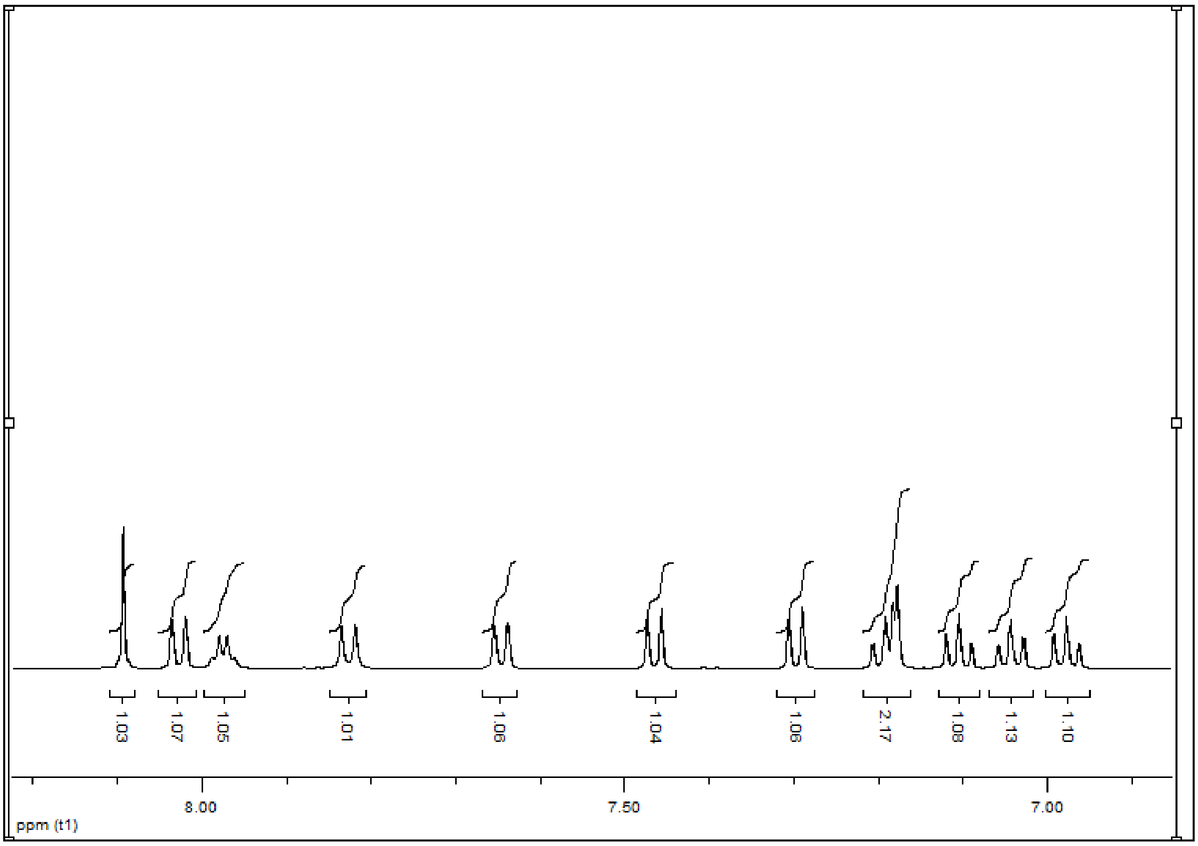

Figure S34. Extended aromatic region ^{1}H NMR (500 MHz, DMSO-d_6) spectrum of compound **D10.**

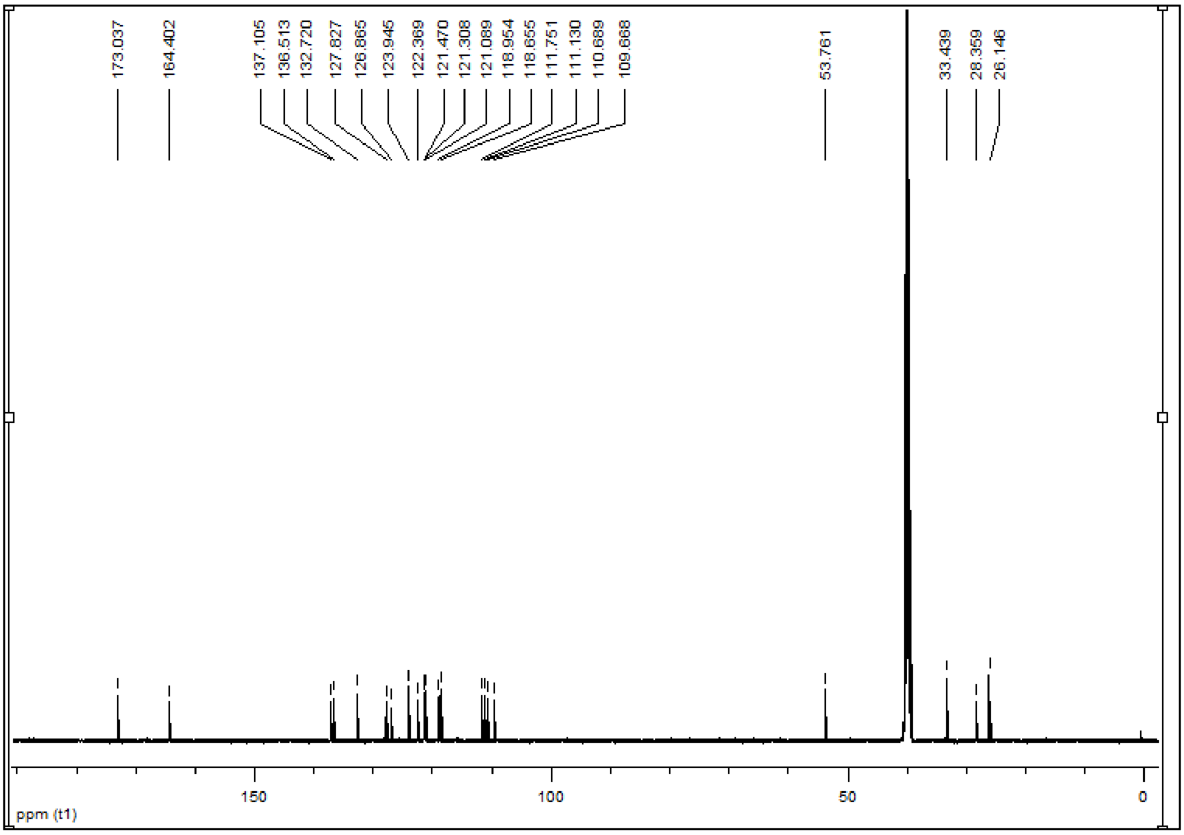

Figure S35. ^{13}C NMR (126 MHz, DMSO-d_6) spectrum of compound **D10.**

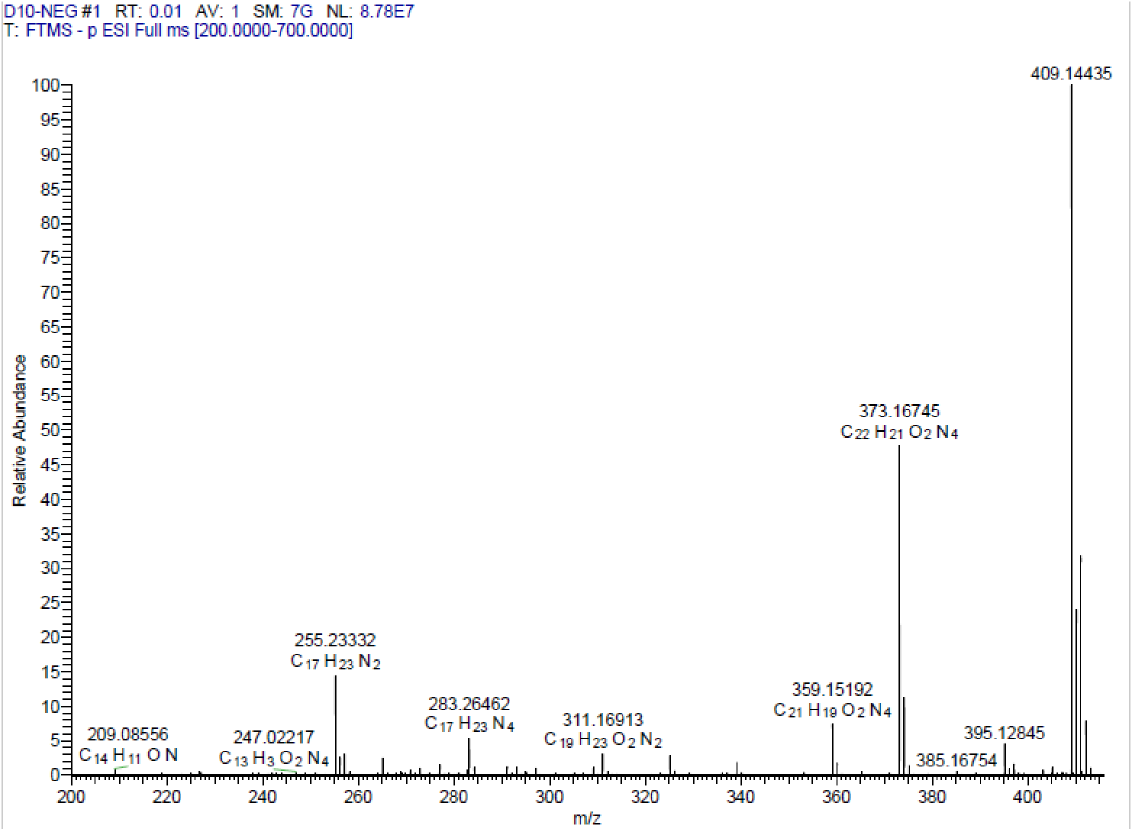

Figure S36. MS spectrum of compound **D10.**

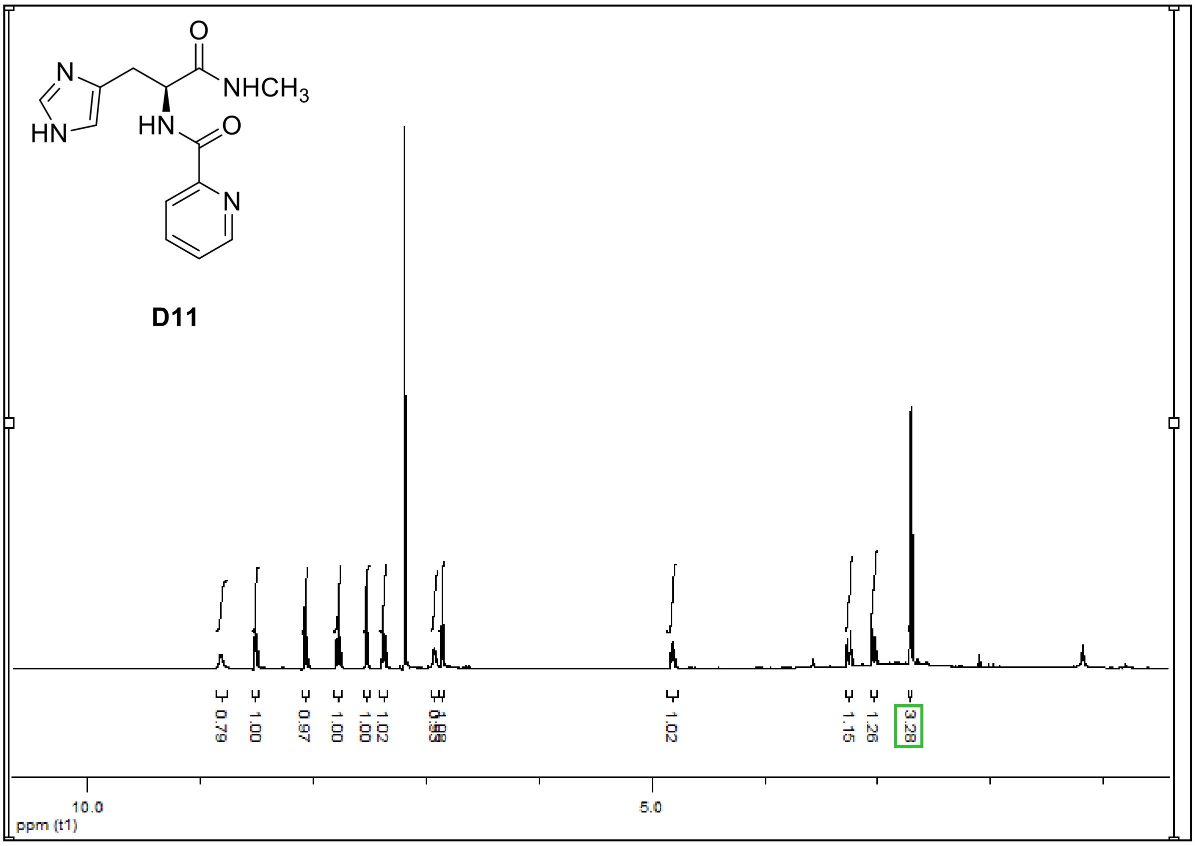

Figure S37. ^{1}H NMR (500 MHz, $CDCl_3$) spectrum of compound **D11.**

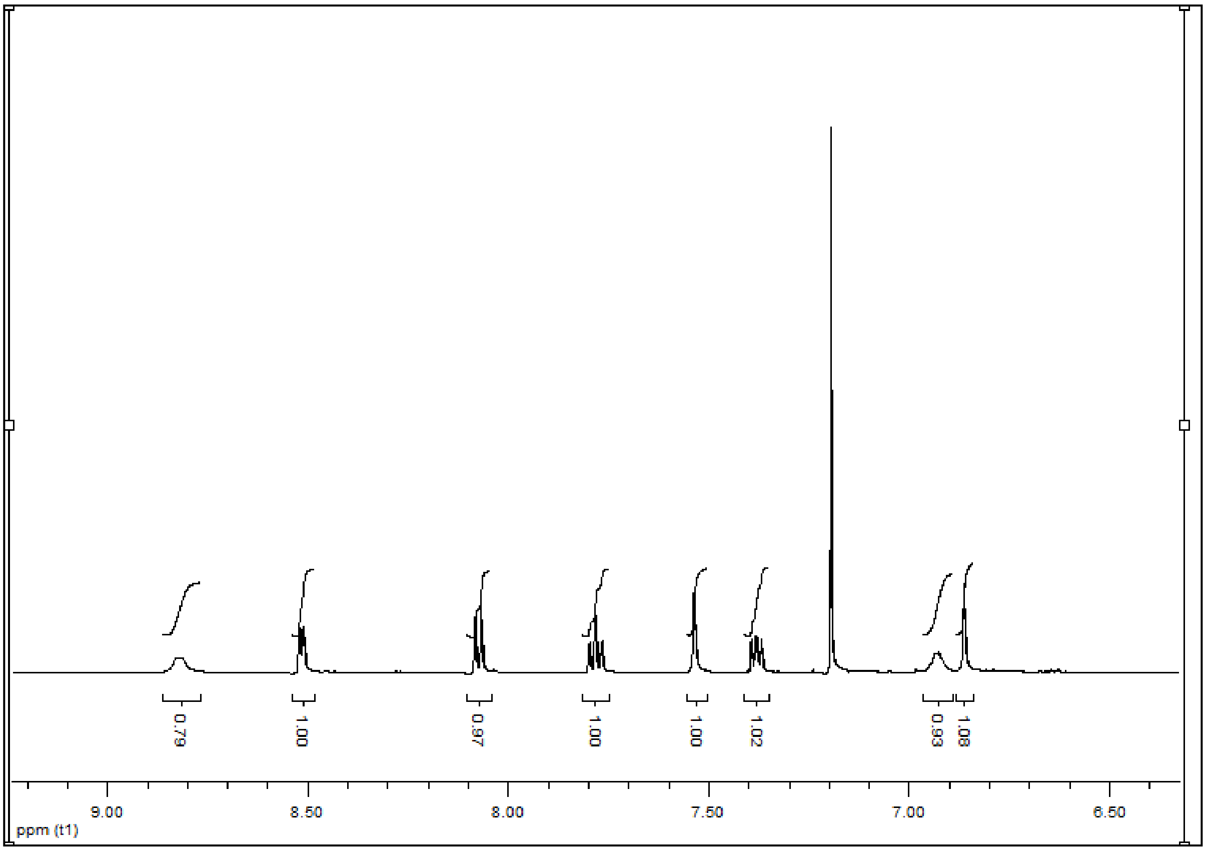

Figure S38. Extended aromatic region ^{1}H NMR (500 MHz, DMSO-d_6) spectrum of compound **D11.**

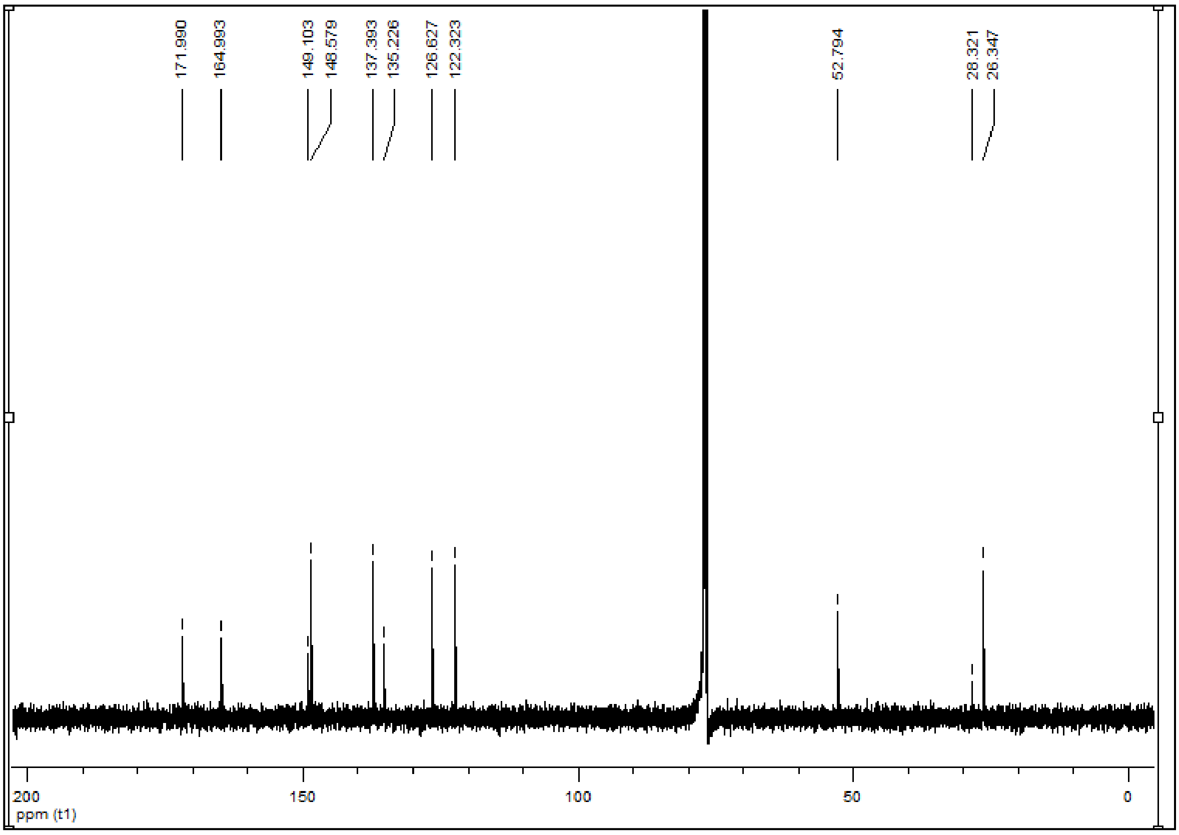

Figure S39. ^{13}C NMR (126 MHz, $CDCl_3$) spectrum of compound **D11.**

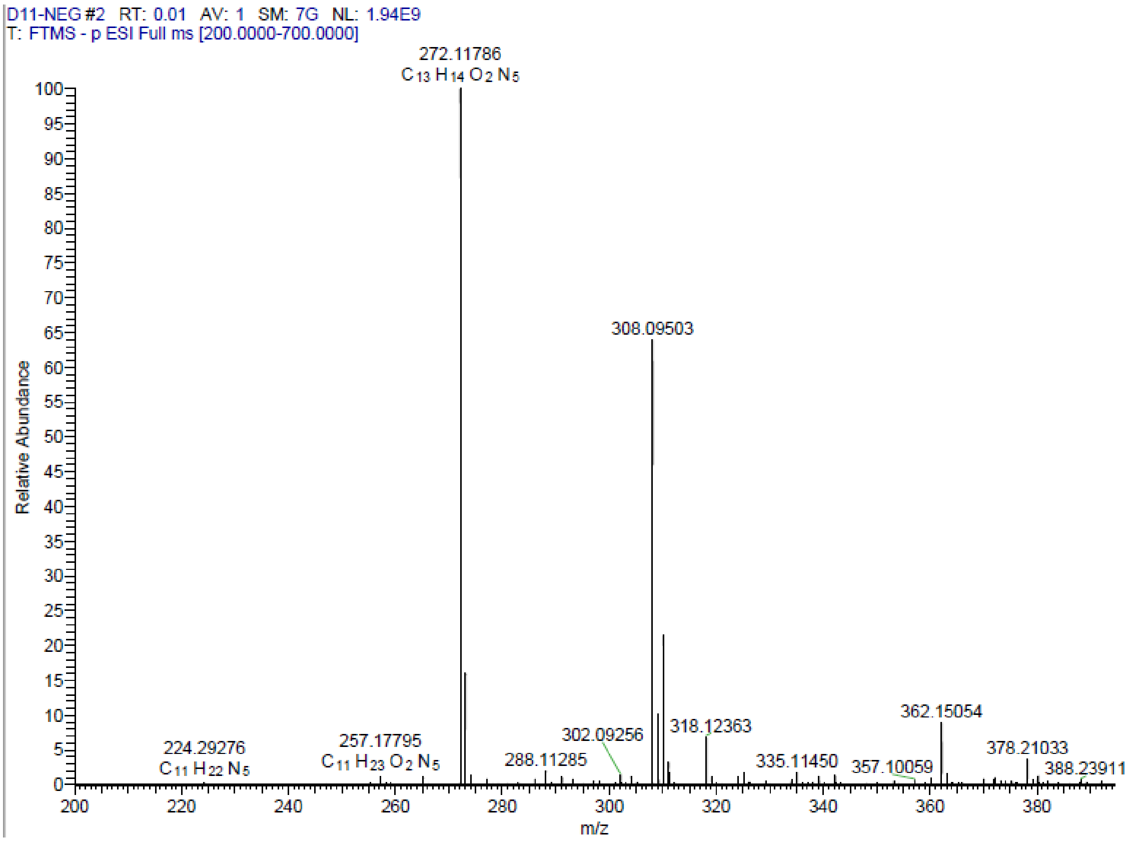

Figure S40. MS spectrum of compound **D11.**

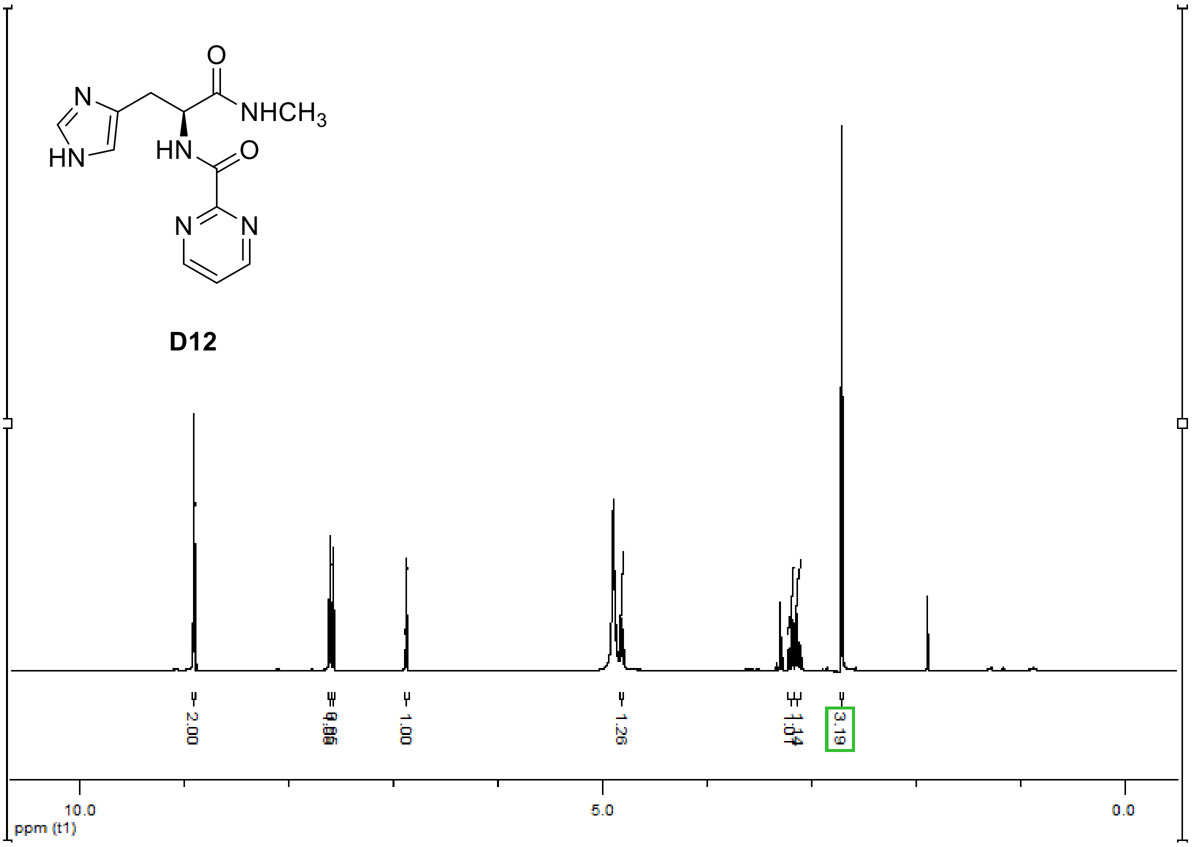

Figure S41. ^{1}H NMR (500 MHz, CD_3OD) spectrum of compound **D12.**

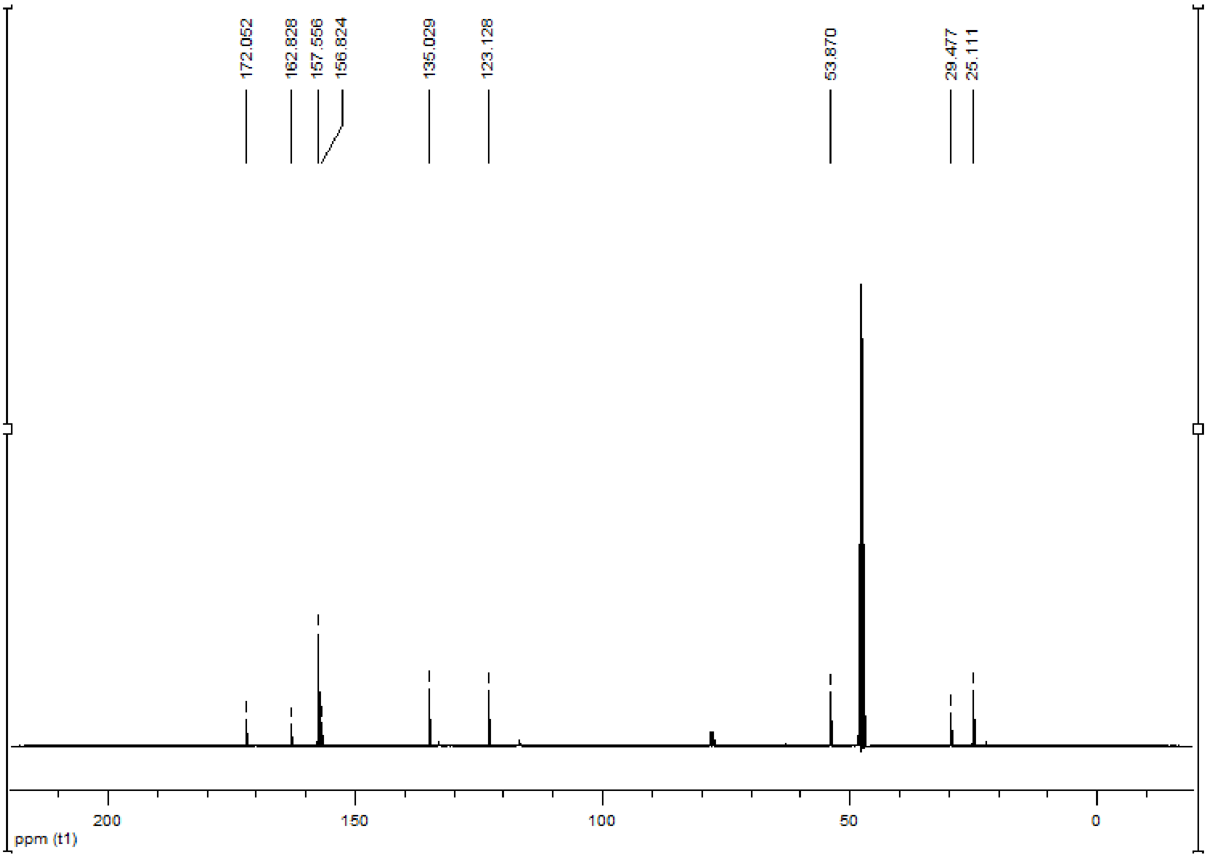

Figure S42. ^{13}C NMR (126 MHz, CD_3OD) spectrum of compound **D12.**

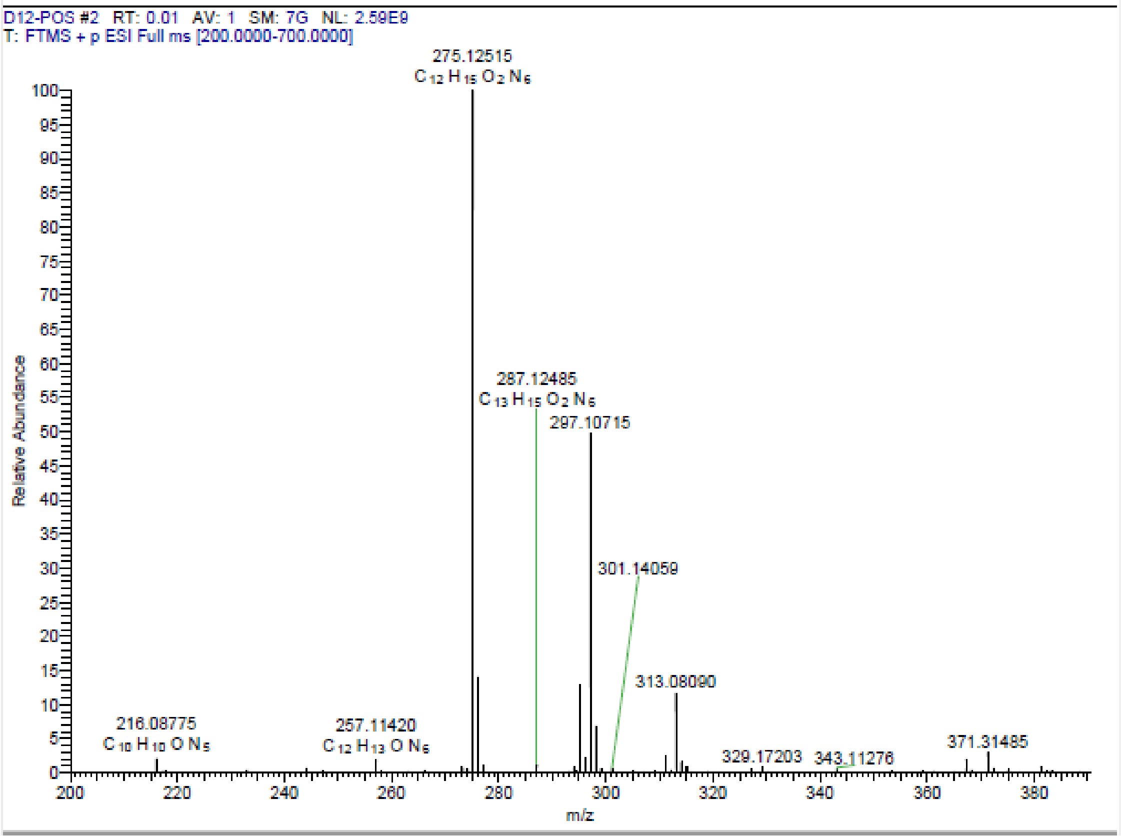

Figure S43. MS spectrum of compound **D12.**

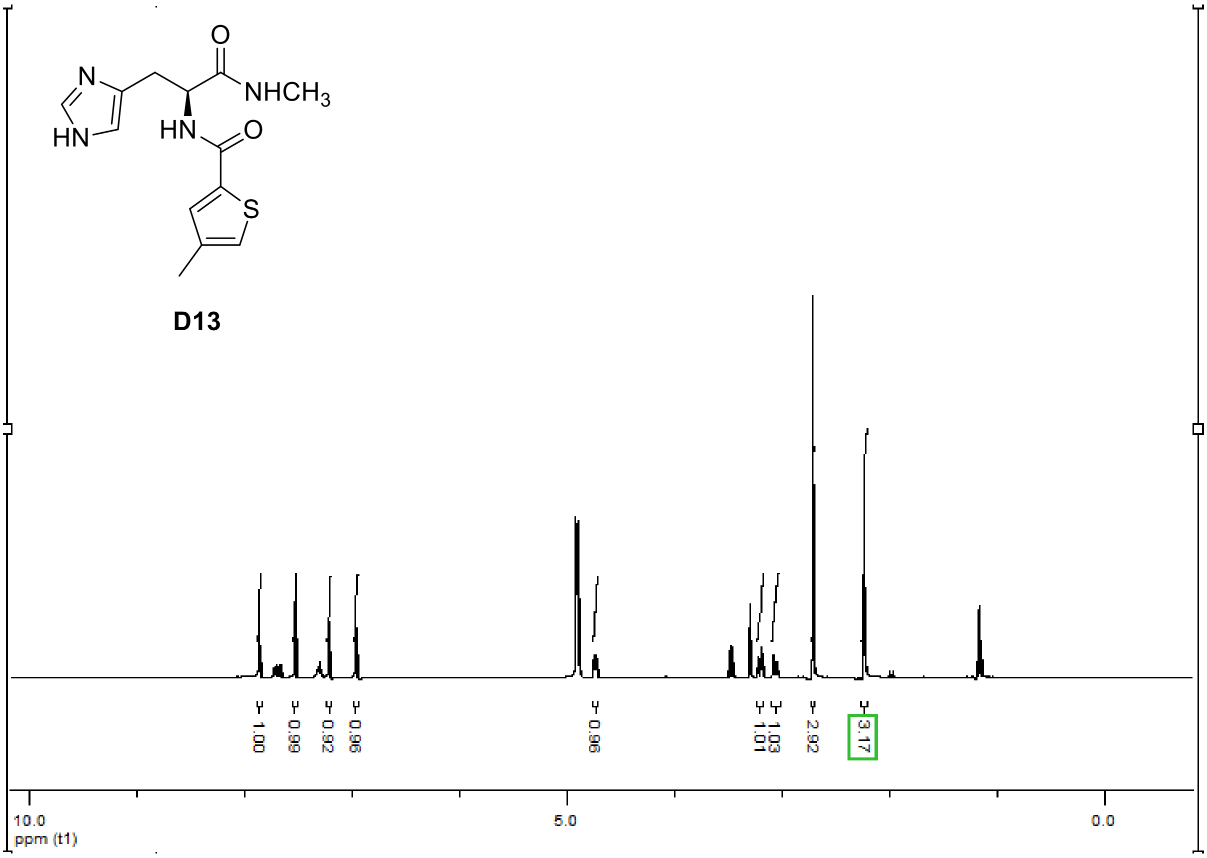

Figure S44. ^{1}H NMR (500 MHz, CD_3OD) spectrum of compound **D13.**

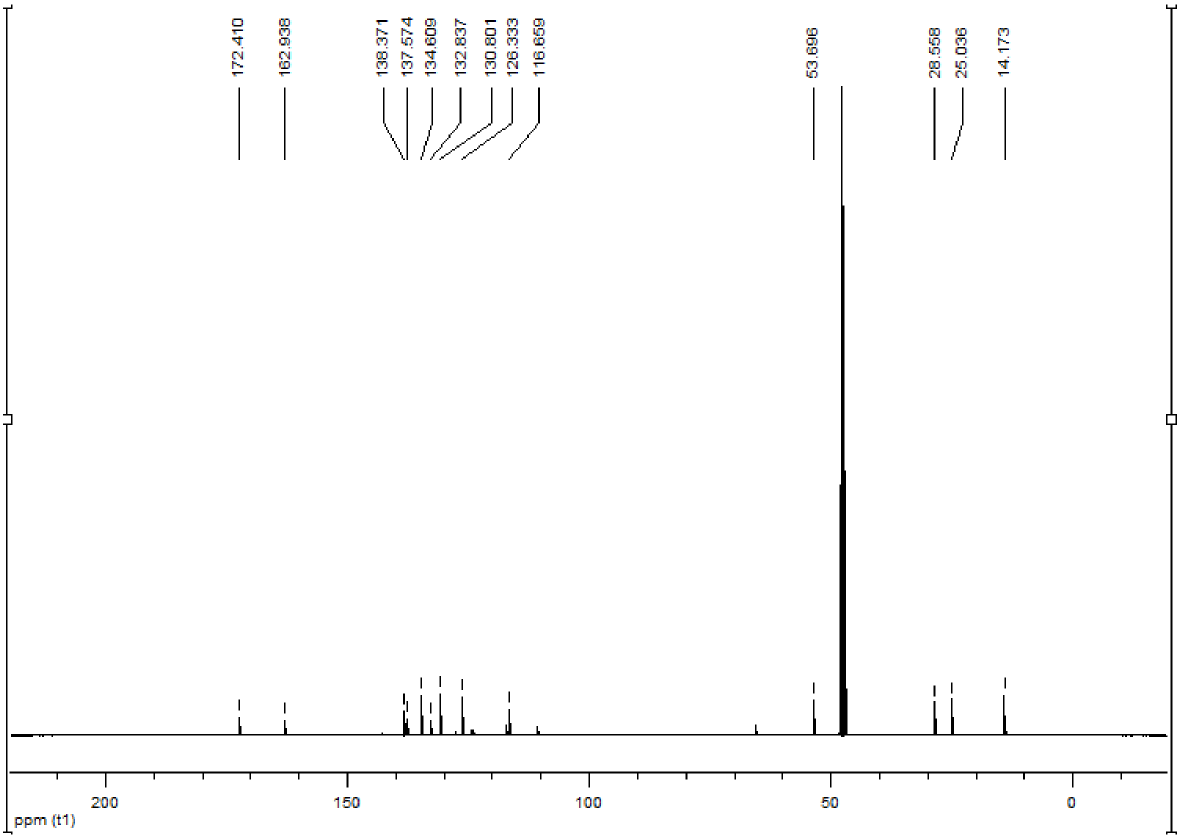

Figure S45. ^{13}C NMR (126 MHz, CD_3OD) spectrum of compound **D13.**

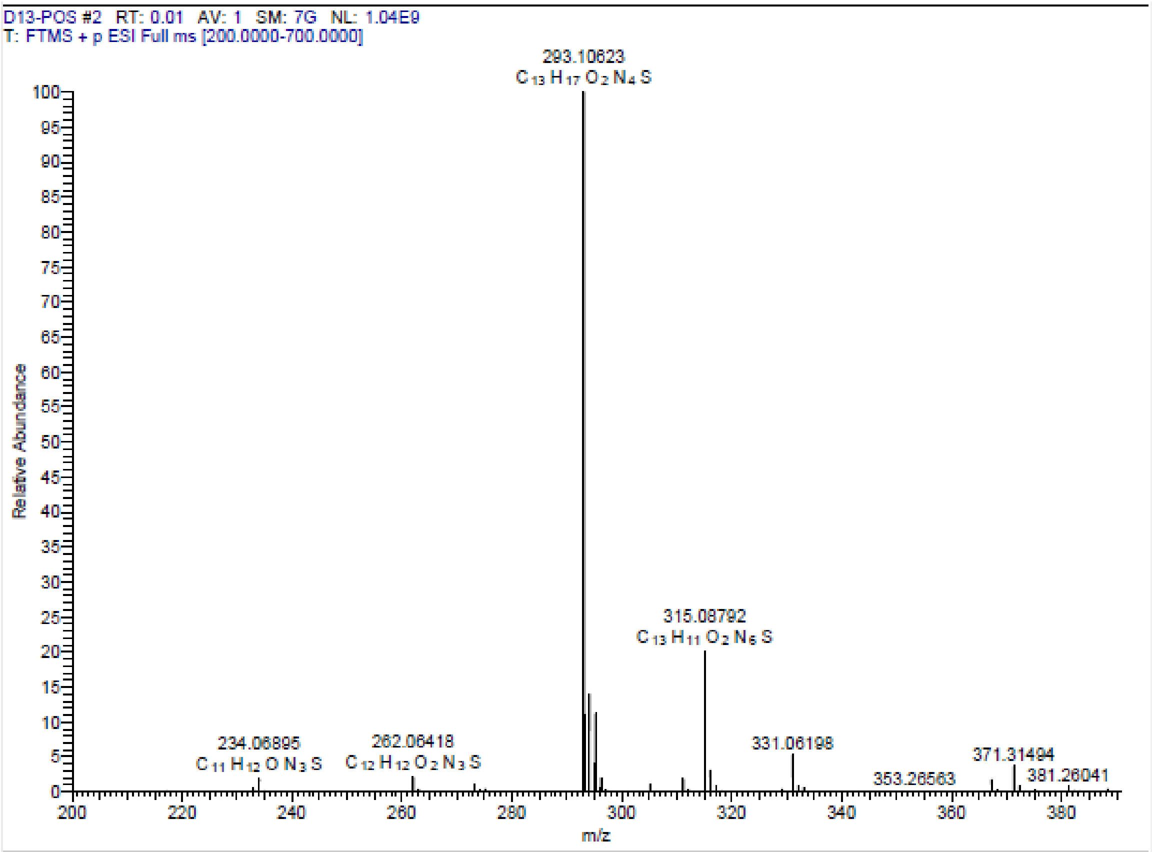

Figure S46. MS spectrum of compound **D13.**

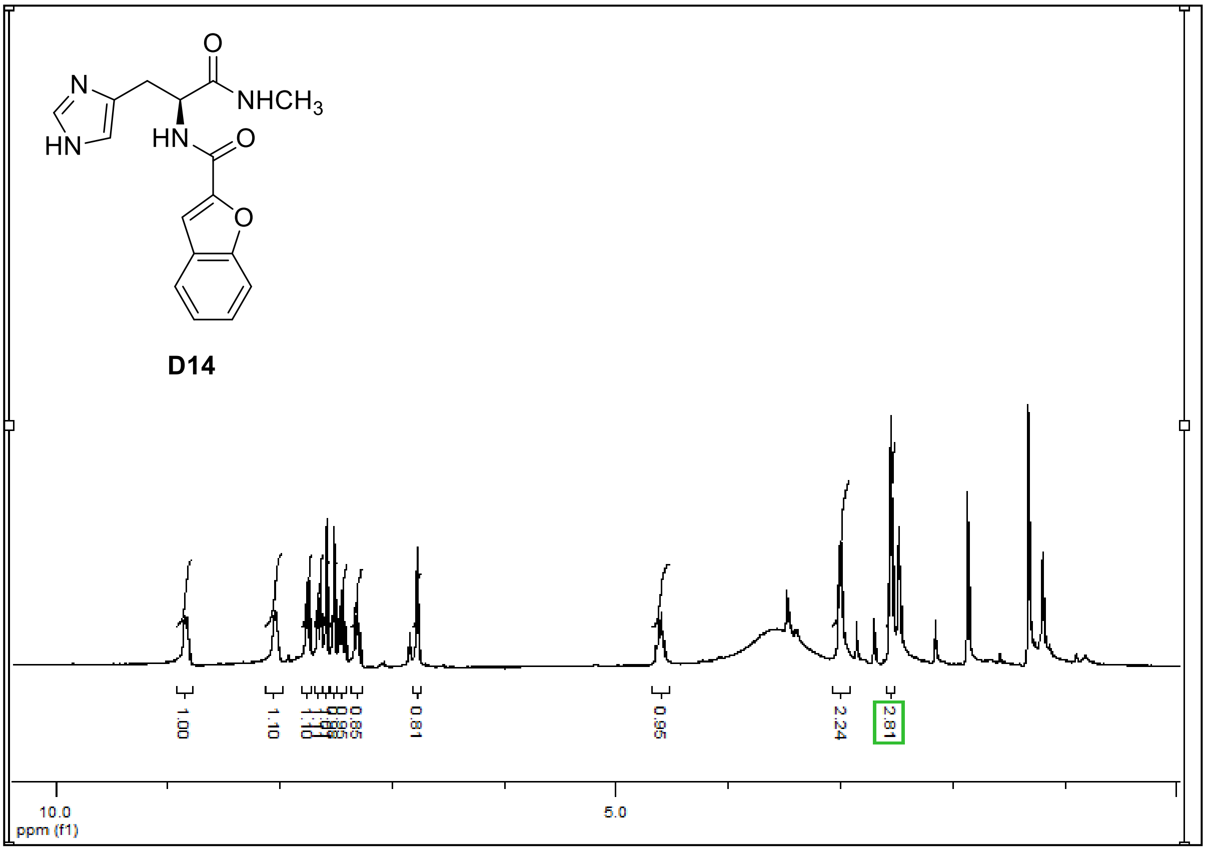

Figure S47. ^{1}H NMR (300 MHz, DMSO-d_6) spectrum of compound **D14.**

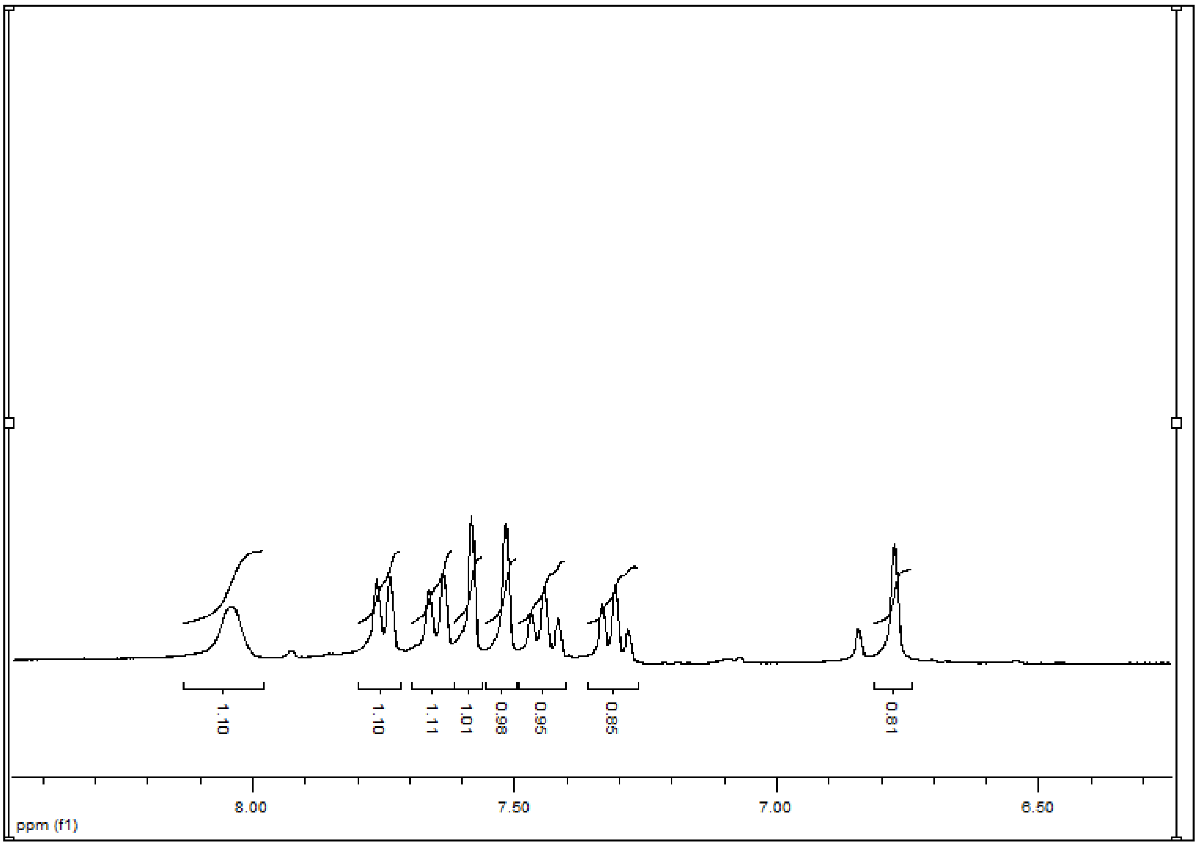

Figure S48. Extended aromatic region ^{1}H NMR (500 MHz, DMSO-d_6) spectrum of compound **D14**.

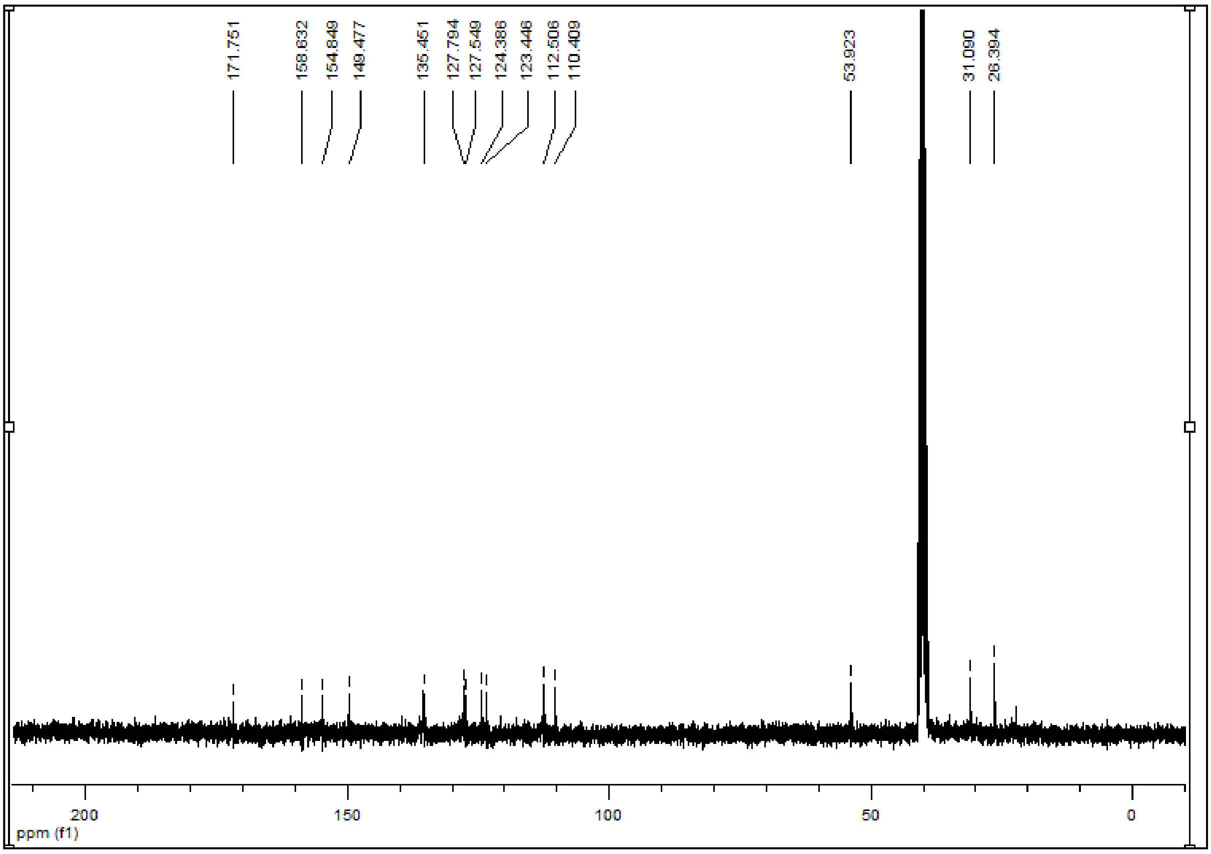

Figure S49. ^{13}C NMR (126 MHz, DMSO-d_6) spectrum of compound **D14.**

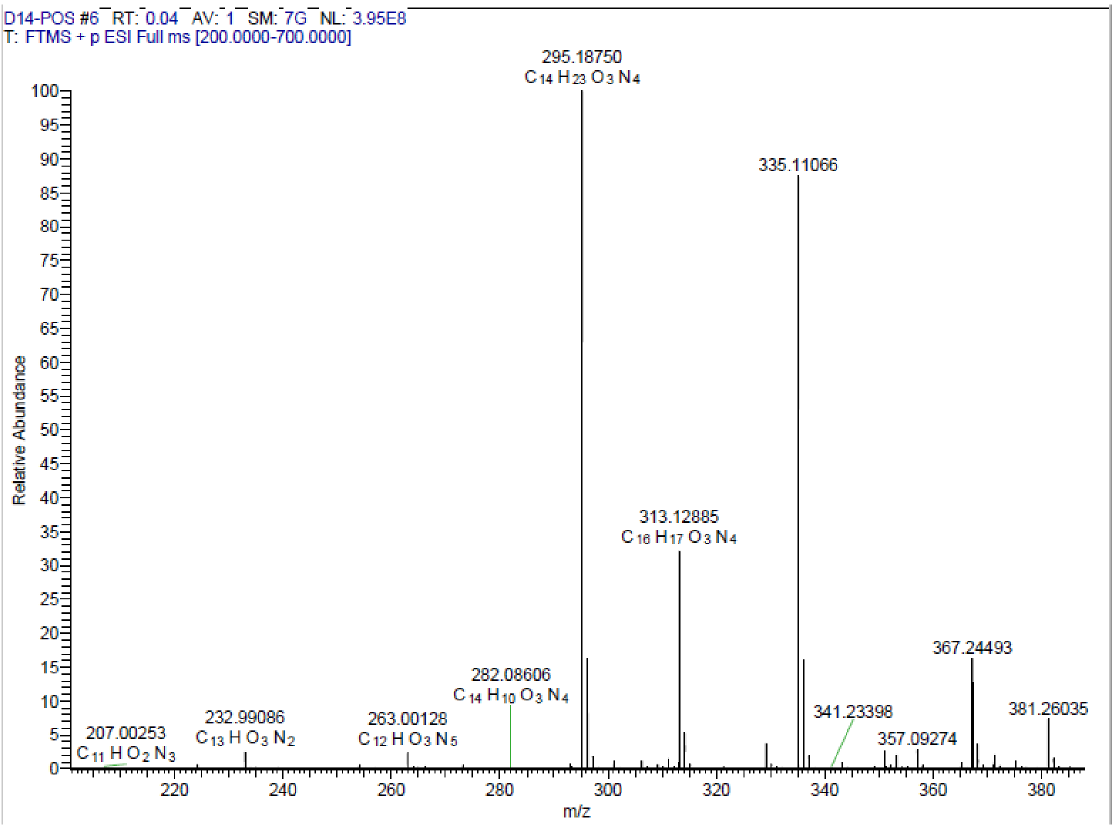

Figure S50. ^{13}C NMR (75MHz, DMSO-d_6) spectrum of compound **D14.**

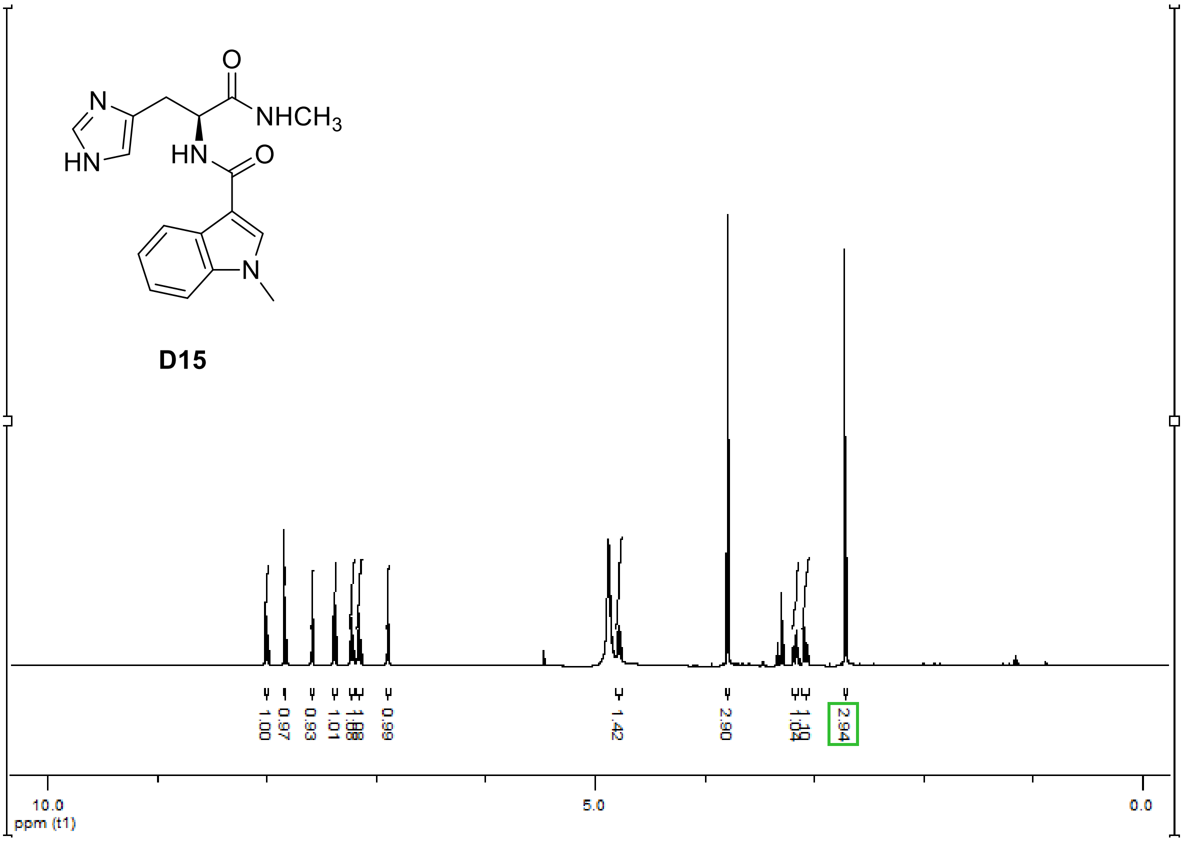

Figure S51. ^{1}H NMR (500 MHz, CD_3OD) spectrum of compound **D15.**

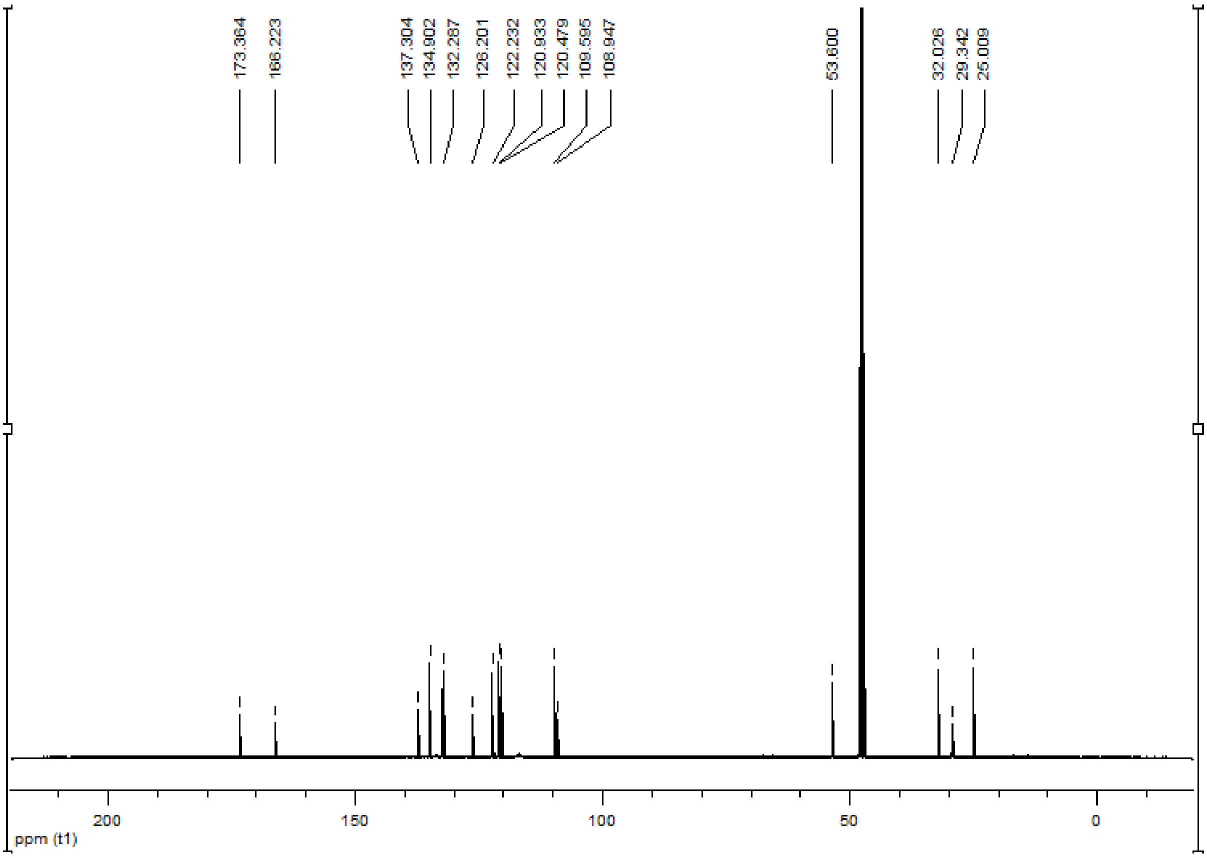

Figure S52. ^{13}C NMR (126 MHz, CD_3OD) spectrum of compound **D15.**

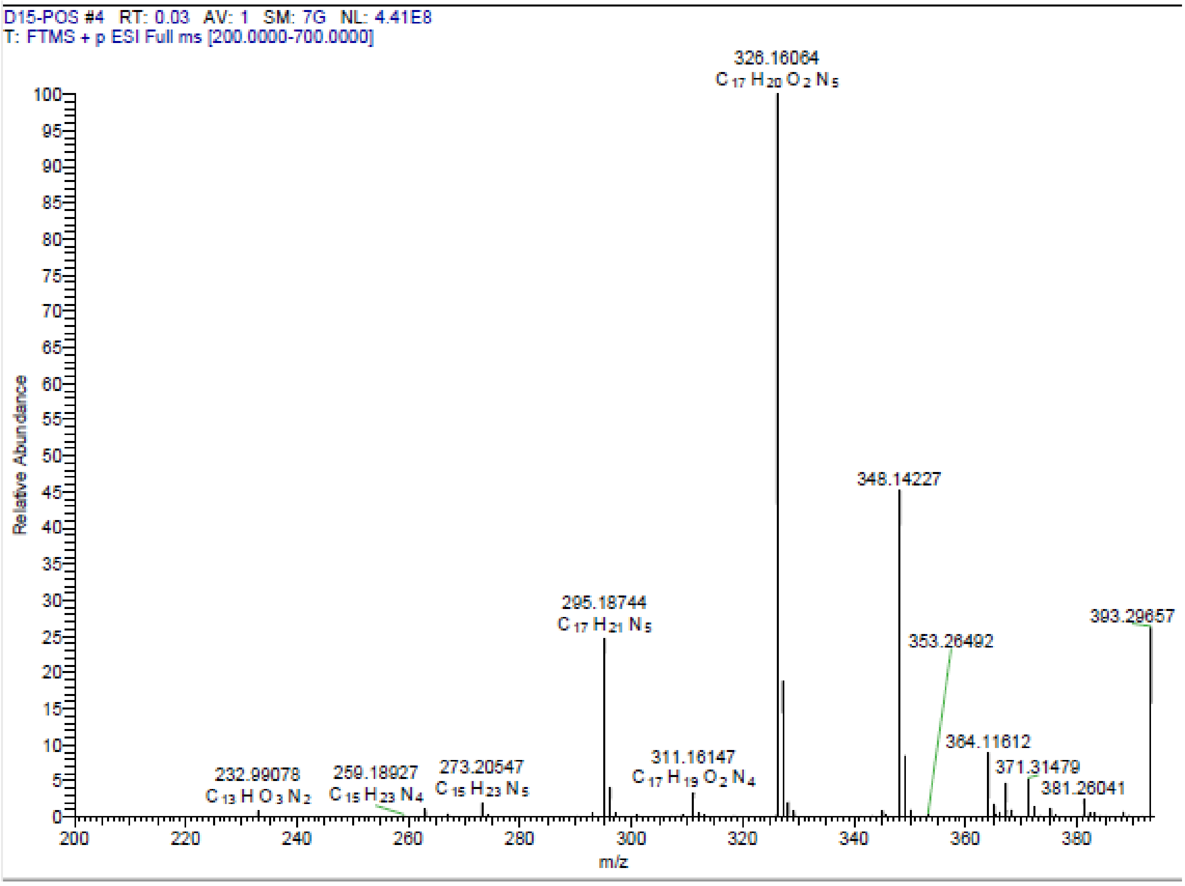

Figure S53. MS spectrum of compound **D15.**
